# Synthesis of the Spirotetracyclic Core of the Ginkgolides via a Malonyl Radical Cascade

**DOI:** 10.1021/acs.orglett.5c02247

**Published:** 2025-07-14

**Authors:** Pol Hernández-Lladó, Kirsten E. Christensen, Jonathan W. Burton

**Affiliations:** ∇ Department of Chemistry, Chemistry Research Laboratory, 6396 University of Oxford, Mansfield Road, Oxford OX1 3TA, U.K.

## Abstract

The ginkgolides are a family of terpene trilactone natural products exclusive to the *Ginkgo biloba* tree. Here, we present a concise synthesis of their spirotetracyclic core via a manganese­(III)-mediated oxidative radical cascade. Beginning from six simple starting materials, this route enables the diastereoselective synthesis of rings A, B, D and E of the natural product in nine steps, laying the foundations for their total synthesis.

## Graphical abstract

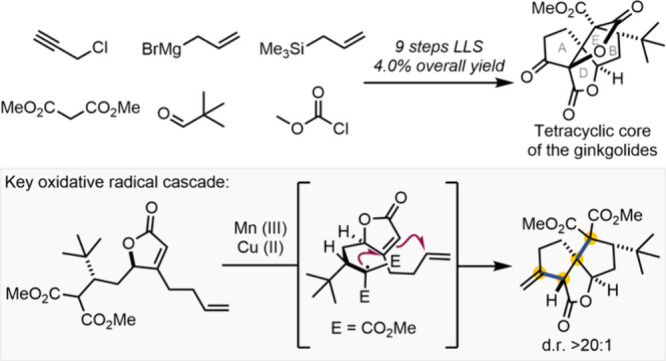

The *Ginkgo biloba* tree, endemic to China and Korea, has been used in traditional medicine for millennia for the treatment of age-related memory loss, inflammation and other ailments.^1^ The extracts from this tree contain several terpene trilactones exclusive to this species namely the ginkgolides (Figure 1A). The structures of the ginkgolides were elucidated by Nakanishi et al. in 1967. This herculean achievement was documented in a series of back-to-back publications, which detailed their isolation, characterization, stereochemistry, and derivatization.^2–6^ The structures were confirmed the same year by X-ray crystallographic analysis by Okabe of the *para*-bromobenzoate of ginkgolide A.^7^ The ginkgolides are potent and selective platelet-activating factor (PAF) receptor antagonists,^8^ yet the potential link between this antagonism and their biological effects remains unknown.^1,9^

**Figure 1:**
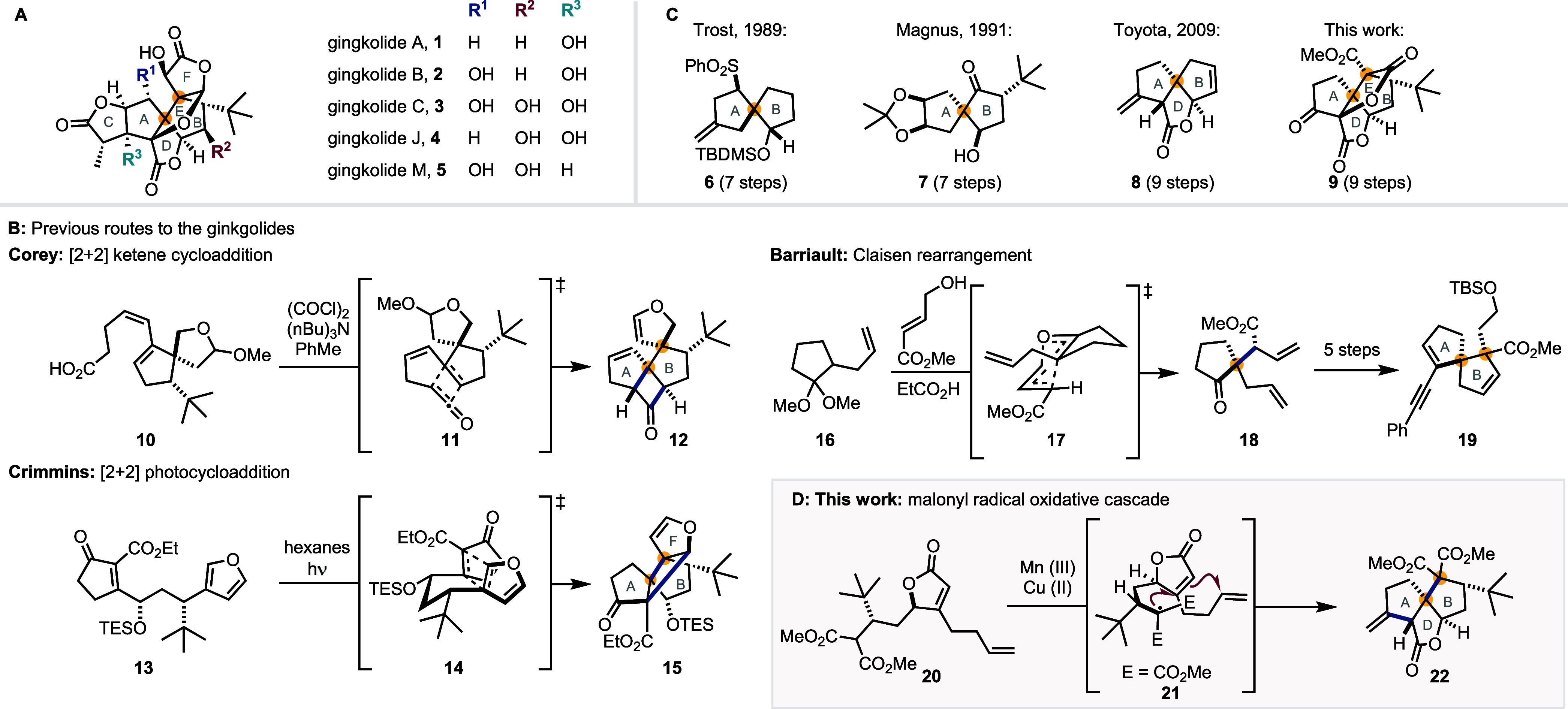
(A) The structure of the ginkgolides (**1–5**). (B) Summary of the steps employed to construct the key contiguous all-carbon stereocenters in previous ginkgolide syntheses. (C) Previously described truncated analogues of the ginkgolides (**6–8**) and this work’s target (**9**). (D) This work’s approach to the core of the ginkgolides.

The structural complexity of the ginkgolides includes a *tert*-butyl group (seldom present in natural products),^10–13^ six heavily functionalized five-membered rings and up to 12 stereogenic centers. Two of the contiguous stereocenters in the ginkgolides are all-carbon quaternary centers (highlighted in yellow in Figure 1A), which are challenging to construct in a stereoselective manner. This stems from the sterically encumbered environment around these centers and the limited number of synthetic methodologies for the stereoselective construction of adjacent all-carbon quaternary centers.^14^

Several members of the ginkgolide family of natural products have been the successful target of total synthesis. In 1988, Corey reported the first total syntheses of (±)-ginkgolides A and B, which overcame the challenging stereoselective construction of the adjacent quaternary centers through an elegant [2 + 2] ketene cycloaddition and subsequent ring expansion (Figure 1B).^15,16^ The same year, Corey et al. published the asymmetric synthesis of an intermediate in this route, thus completing the first, and to this day only, (formal) enantioselective synthesis of any member of the ginkgolide family.^17^ In 1999, Crimmins reported the second total synthesis of (±)-ginkgolide B, which also employed a [2 + 2] cycloaddition to forge the contiguous quaternary stereocenters (Figure 1B).^18,19^ More recently, in 2022, Barriault described the first total synthesis of (±)-ginkgolide C and formal synthesis of (±)-ginkgolides A and B.^20^ In this instance, the adjacent all-carbon stereocenters were constructed via a diastereoselective Claisen rearrangement followed by an enolate alkylation (Figure 1B).

Although the published synthetic routes to the ginkgolides differ significantly in synthetic strategy and execution, they all employed pericyclic reactions to construct at least one of the all-carbon quaternary stereocenters.^14^ Second, the *tert*-butyl group was incorporated into these natural products via the copper-mediated conjugate addition/S_N_2’ reaction of *tert*-butyl lithium.

Further to these studies, the synthesis of truncated analogues of the ginkgolides has also been investigated (Figure 1C). Trost published a cycloaddition strategy to rings A and B of the ginkgolides, in this instance using a [3 + 2] palladium-catalyzed cycloaddition to synthesize spirocycle **6**.^21^ Magnus constructed these same rings via alkylation of cyclopentadiene and oxidation with singlet oxygen.^22^ Finally, Toyota and co-workers described the synthesis of spirotricycle **8** through a Pd­(II)-catalyzed oxidative cyclization.^23^ None of the reported syntheses of truncated fragments, however, included the construction of the adjacent all-carbon stereocenters.

Manganese­(III)-mediated oxidative radical cascades are powerful reactions for the rapid generation of complexity and, as such, have been widely used in complex target synthesis,^24^ including in the synthesis of molecules with adjacent all-carbon quaternary centers.^25^ Our group has extensive experience in this area, as exemplified by our syntheses of salinosporamide A,^26^ the avenaciolide natural products,^27^ and aphanamol I.^28^ We envisaged such a cascade could be employed to construct the challenging all-carbon quaternary stereocenters of the ginkgolides (Figure 1D). Radical cascades are particularly suited for the construction of contiguous quaternary centers due to the high reactivity of radicals, their tolerance of steric hindrance, and predictable stereo and regiochemistry. They have been widely employed in the synthesis of natural products.^14,24,29–39^ Here, we describe the concise synthesis of the spirotetracyclic core of the ginkgolides (**9**), which features an oxidative radical cascade to forge the key adjacent all-carbon quaternary centers.

A summary of our retrosynthetic analysis toward the tetracyclic core of the ginkgolides (**9**) is shown in Figure 2. Our proposed route was based on the oxidative radical cascade of γ-butenolide **23**, which we envisaged would be used to construct rings A, B and D of the ginkgolide core (**22**) and set the relative configuration of two stereocenters (highlighted in yellow in Figure 2). Ring E would be built last (giving **9**) via olefin cleavage, α-hydroxylation and lactonization of the newly installed hydroxy group with one of the diastereotopic methyl esters of the malonate of tricycle **22**. The substrate of the key radical cascade, γ-butenolide **23**, would in turn be prepared through the addition of the organomagnesium reagent derived from alkenyl iodide **25** to aldehyde **24**, employing chemistry developed by Knochel and co-workers.^40^ Aldehyde **24** and vinyl iodide **25** could be prepared from simple, readily available starting materials (**26**–**31**).

**Figure 2:**
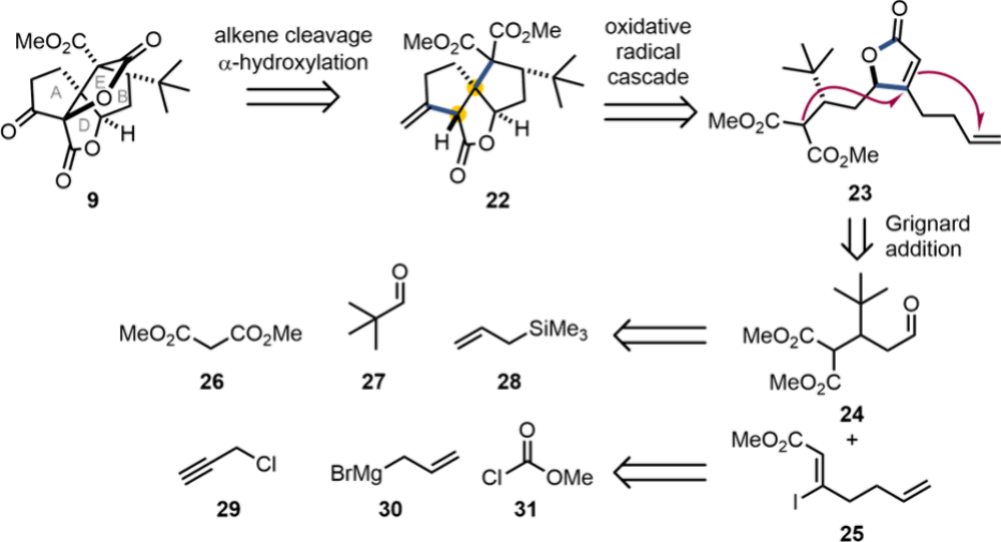
Summary of the retrosynthetic analysis of the spirotetracyclic core of the ginkgolides (**9**).

We began our studies with the synthesis of aldehyde **24** and vinyl iodide **25** (Scheme 1). Aldehyde **24** was obtained in three steps from pivalaldehyde (**27**) through an indium-mediated Knoevenagel condensation,^41^ Sakurai allylation and Lemieux-Johnson oxidation.^42^ Vinyl iodide **25** was prepared in two steps from propargyl chloride (**29**). First, alkynyl ester **33** was obtained through the one-pot substitution of propargyl chloride (**29**) with allylmagnesium bromide and methoxycarbonylation of the resulting alkynylmagnesium as developed by Hopf.^43^ Subsequently, conjugate addition of iodide under acidic conditions provided vinyl iodide **25** from alkynyl ester **33**.^44^

**Scheme 1:**
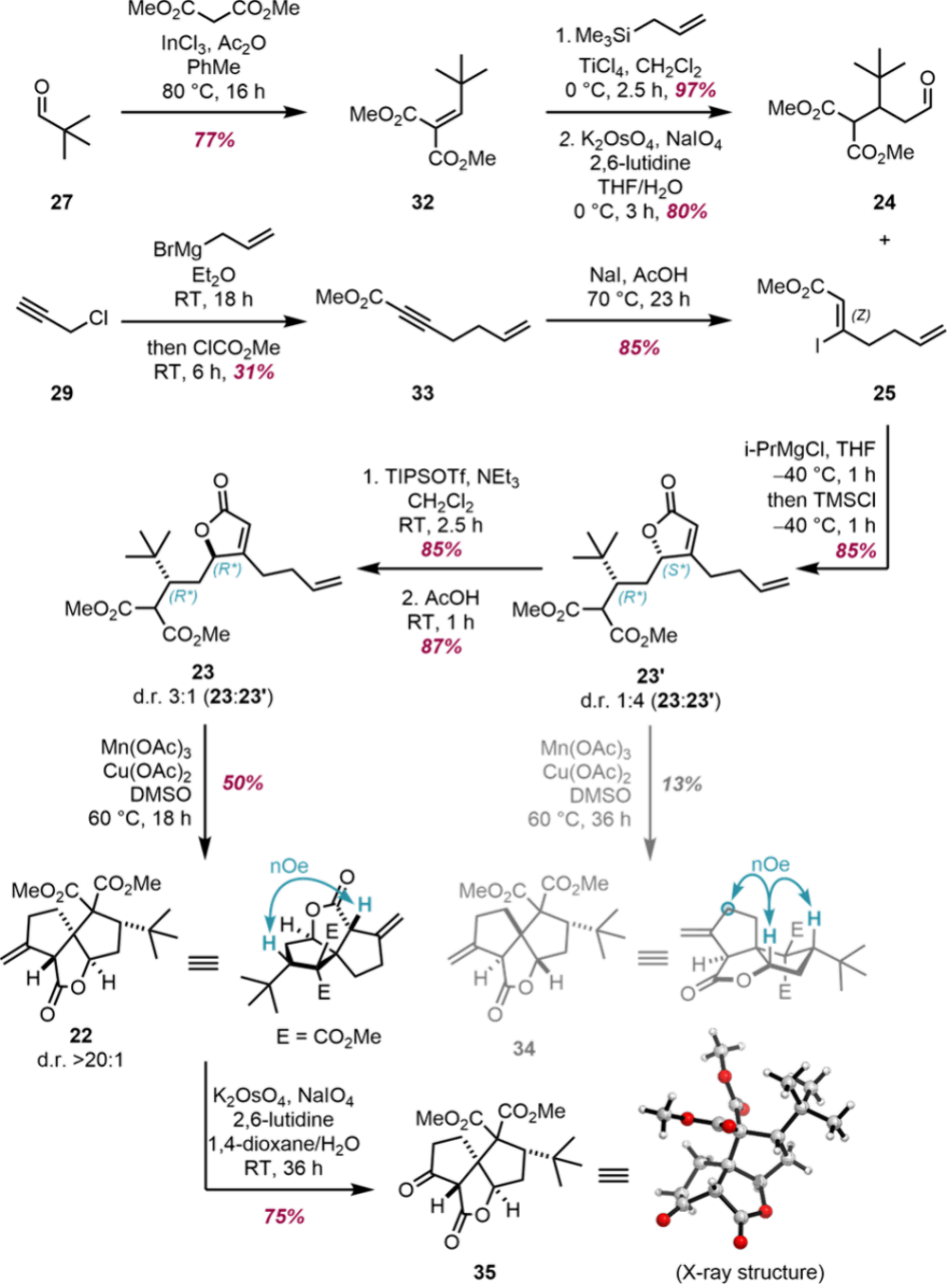
Synthesis of Aldehyde **24** and Vinyl Iodide **25**, Their Conversion to γ-Butenolides **23** and **23′**, and Radical Cyclization

With aldehyde **24** and vinyl iodide **25** in hand we investigated the synthesis of γ-butenolide **23** (Scheme 1). Treatment of aldehyde **24** with the Grignard reagent derived from vinyl iodide **25** in the presence of trimethylsilyl chloride yielded *γ-*butenolide **23′** as the major diastereomer (4:1 d.r.) in 85% yield, having the undesired *syn*relative configuration. Diastereoisomers **23** and **23′** were inseparable and their relative configuration could not be determined from NMR analysis. Instead, their relative configuration was assigned from the configurations of the oxidative cyclization products (*vide infra*). Initial cyclization studies showed that on treatment of γ-butenolide **23′** (d.r. 4:1) with Mn­(OAc)_3_ and Cu­(OAc)_2_ in DMSO,^45^ tricycle **34** was obtained in 13% yield, the relative configuration of which could be determined based on key ^1^H NMR NOE interactions (Scheme 1). The configuration of the stereocenters in tricycle **34** was thus not the one required for the synthesis of the ginkgolides, however, it allowed the assignment of the stereocenters in γ-butenolide **23′** as *syn* (R*, S*). Attempts to epimerize γ-butenolide **23′** to mixtures of **23′** and **23** under acidic or basic conditions were unsuccessful and led to recovery of γ-butenolide **23′** (see Supporting Information, S53).

The isomerization of γ-butenolide **23′** into **23** was ultimately achieved through silylation of γ-butenolide **23′** to give the corresponding silyloxyfuran, followed by desilylation in acetic acid. This protocol yielded the desired *anti*-isomer **23** in a 3:1 diastereomeric ratio with **23′**. The desilylation reaction was highly sensitive to the acid employed, with other acids yielding mixtures favoring the *syn*-isomer **23′** or containing the undesired α-butenolide (see Supporting Information, S53).

The oxidative radical cascade of γ-butenolide **23** (containing 25% of *syn*-diastereoisomer **23′**) yielded tricycle **22** in 50% yield and excellent diastereoselectivity (Scheme 1). The diasterocontrol likely arises from the kinetic and thermodynamic preference to form *cis*-fused bicyclo[3.3.0]­octanes coupled with the *tert*-butyl group residing on the less hindered convex face of the forming bicyclooctane.^46,47^ The relative configuration of tricycle **22** was subsequently confirmed by X-ray crystallography of cyclopentanone **35**, obtained *via* Lemieux-Johnson oxidation of **22**. (Scheme 1).

The last step to access the spirotetracyclic core of the ginkgolides (**9**) was the hydroxylation of cyclopentanone **35** and subsequent lactonization to construct ring E. Based on precedent in the total syntheses of ginkgolide B by Corey and Crimmins, we initially envisaged deprotonation of cyclopentanone **35** with lithium diethylamide followed by oxidation of the so-formed lithium enolate with Davis oxaziridine could yield the required product **9** (Scheme 2).^15,16,18,19^ Unfortunately, under these reaction conditions cyclopentanone **35** degraded and no hydroxylation products were observed. Alternative oxidants were equally unsuccessful in this reaction, with tetrabutylammonium fluoride/dimethyldioxirane (TBAF/DMDO), giving instead the δ-lactone **39**, the product of formal Baeyer–Villiger oxidation.

**Scheme 2:**
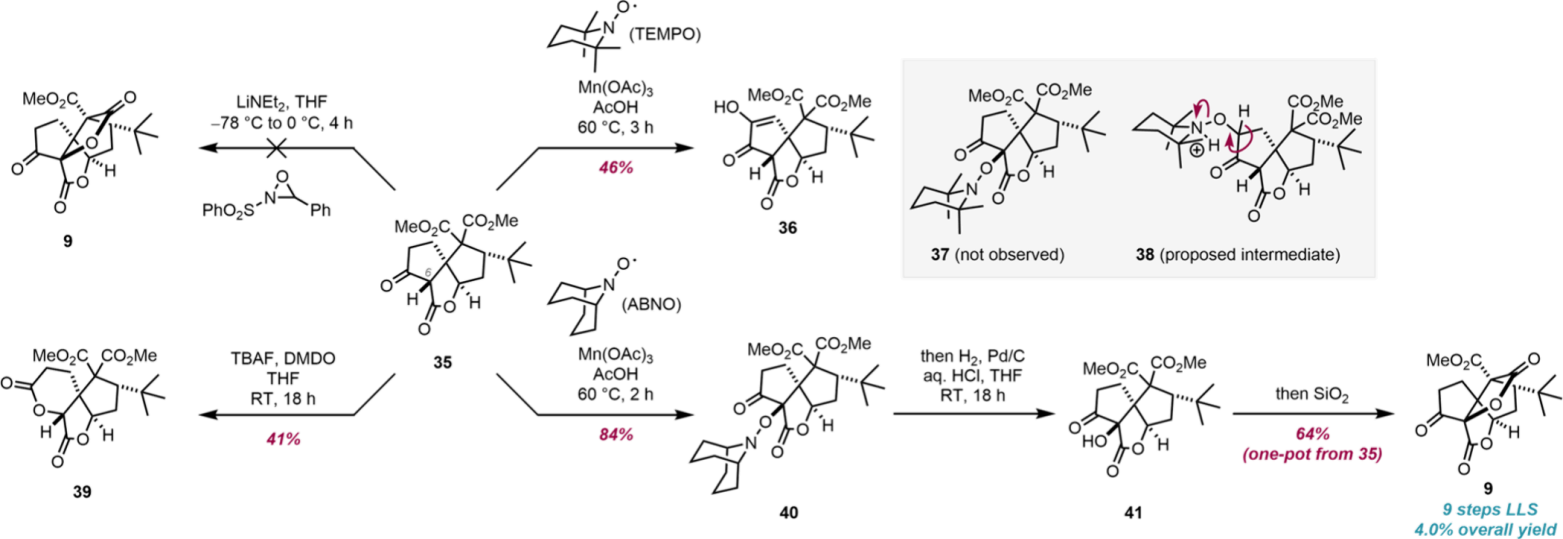
Construction of Ring E *via* Hydroxylation of Cyclopentanone **35** and Summary of Unsuccessful Approaches

We envisioned an alternative two-step sequence based on the Mn­(OAc)_3_-mediated oxidation of cyclopentanone **35** in the presence of TEMPO to give alkoxyamine **37** followed by a reductive *N–O* bond cleavage (Scheme 2). This was inspired by the work of Terent’ev et al., who employed Mn­(OAc)_3_ in their dehydrogenative coupling of 1,3-dicarbonyl compounds with oximes and *N*-hydroxyimides;^48,49^ and Jahn and co-workers, who reported a two-step α-hydroxylation method comprising the oxidation of lithium enolates with ferrocenium cation, coupling with TEMPO, and reductive *N*-*O* bond cleavage.^50^ However, upon treatment of cyclopentanone **35** with Mn­(OAc)_3_ and TEMPO, alkoxyamine **37** was not observed and hydroxyenone **36** was obtained instead in 46% yield. We hypothesized enone **36** was formed *via* Kornblum-DeLaMare-like rearrangement of TEMPO adduct **38**, which was preferentially formed over adduct **37** presumably due to the steric hindrance around C(6) in cyclopentanone **35**. Based on this hypothesis, we attempted this same α-oxidation reaction of cyclopentanone **35** with the less sterically demanding oxyl radical ABNO. Indeed, this reaction gave a completely different outcome, yielding desired C(6)-alkoxyamine **40** in 84% yield. Palladium-catalyzed hydrogenolysis of alkoxyamine **40** resulted in the required *N–O* bond cleavage to give alcohol **41**.

The lactonization of alcohol **41** to give the core of the ginkgolides (**9**) was, contrary to our initial predictions, not spontaneous. Small quantities of tetracycle **9**, however, were detected upon chromatographic purification of alcohol **41**. The observation that silica was facilitating the lactonization of alcohol **41** allowed the development of a mild, silica-mediated lactonization procedure. The formal hydroxylation and lactonization of tricycle **35** could ultimately be performed in a one-pot fashion after minor modifications, yielding the spirocyclic core (**9**), containing rings A, B, D, and E of the ginkgolides, in 64% isolated yield from **35**.

In summary, we have developed a concise synthesis of the spirotetracyclic core of the ginkgolides (**9**) containing rings A, B, D, and E in 9 steps and 4.0% overall yield, starting from six simple starting materials.^51^ This approach successfully addresses the challenging construction of the contiguous all-carbon stereocenters characteristic of the ginkgolides, achieved *via* a highly diastereoselective Mn­(OAc)_3_-mediated oxidative cascade. This represents a departure from previous strategies that used pericyclic reactions to set at least one of the all-carbon quaternary centers.^16,18,19^ The same oxidant was employed in a one-pot alkoxyamination/*N*–*O* bond cleavage sequence to construct ring E. An additional highlight of this synthesis is the incorporation of the *tert*-butyl group from pivalaldehyde in the initial step, in contrast to prior methods using *tert*-butyl lithium-derived organocopper reagents. Ongoing efforts are focused on extending this work toward the total synthesis of the ginkgolides, and further results will be reported in due course.

## Supplementary Material



## Data Availability

The data underlying this study are available in the published article and its Supporting Information.

## Notes

Detailed experimental procedures, characterization data and crystallographic data for compound **35** (PDF)

## †

Author to whom correspondence regarding X-ray crystallography should be addressed.
