# Supplementary material for: Synthesis of the Spirotetracyclic Core of the Ginkgolides via a Malonyl Radical Cascade
Source: Org Lett. 2025 Jul 14;27(29):7927–32. doi: 10.1021/acs.orglett.5c02247 (PMC12305663; doi:10.1021/acs.orglett.5c02247)
Supplement: Supplementary file 1 [file ol5c02247_si_001.pdf]

Supporting information

Synthesis of the spirotetracyclic core of the ginkgolides *via* a malonyl radical cascade

Pol Hernández-Lladó, Kirsten E. Christensen, Jonathan W. Burton*

Department of Chemistry, Chemistry Research Laboratory
University of Oxford,
Mansfield Road, Oxford OX1 3TA, UK.

E-mail: jonathan.burton@chem.ox.ac.uk
Homepage: https://burton.web.ox.ac.uk

Contents

1. General Experimental Details 2
2. Compound Synthesis and Characterisation 4
3. NMR Spectra 19
4. Studies on the epimerisation of *γ*-butenolides 53
5. X-ray crystallographic data 54
6. References 57

1. General Experimental Details

Solvents and reagents

All reactions were performed using flame-dried reaction vessels under an atmosphere of argon unless stated otherwise. Anhydrous diethyl ether (Et_2O), dichloromethane (CH_2Cl_2), *N*,*N*-dimethylformamide (DMF), acetonitrile (MeCN), tetrahydrofuran (THF), and toluene (PhMe) were obtained from solvent dispenser units having been passed through an activated alumina column under argon. Methanol was dried over 4 Å molecular sieves under argon. All chemical reagents used were commercially available from Alfa Aesar, Fluorochem and Sigma-Aldrich and used as supplied.

Chromatography

Thin layer chromatography (TLC) was carried out using Merck aluminium-backed TLC Silica Gel 60 F254 pre-coated plates (particle size 0.2 mm). Plates were visualised by the quenching of fluorescence under ultraviolet light (λ_{max} = 254 nm) and by staining and heating with $KMnO_4$ or vanillin. Flash column chromatography was performed using Merck Geduran 60 silica gel (particle size 40–63 μm) with the solvent system given. HPLC analysis was performed on an Agilent 1200 series using a Daicel Chiralpak IA column (4.6 mm × 250 mm, 5 μm). All solvents used for chromatography purification were HPLC grade or equivalent and supplied by Sigma Aldrich.

NMR spectroscopy

^{1}H NMR and ^{13}C NMR spectra were recorded on Bruker AVIII HD 400 nanobay (400/101 MHz), Bruker AVII 500 (500/126 MHz), and Bruker NEO 600 (600/151 MHz) spectrometers in deuterated solvents. ^{13}C NMR spectra were recorded with broadband decoupling. Chemical shifts (δ_H, δ_C) are reported in parts per million (ppm) to the nearest 0.01 ppm for ^{1}H NMR and and 0.1 ppm for ^{13}C NMR. ^{1}H and ^{13}C NMR spectra were referenced relative to the solvent

residual peak. Peak assignments were made on the basis of chemical shifts, integrations, coupling constants, and comparison to known compounds, using COSY, HSQC, and HMBC experiments where appropriate. Multiplets are described as singlet (s), doublet (d), triplet (t), quartet (q), multiplet (m), broad (br), or combinations thereof. Coupling constants (*J*) are reported to the nearest 0.5 Hz.

Mass spectrometry

High resolution mass spectrometry (HRMS) was recorded by the staff of the Chemistry Research Laboratory Mass Spectrometry facilities using a Bruker MicroTOF spectrometer. The mass reported is that containing the most abundant isotopes, with each value to 4 or 5 decimal places and within 5 ppm of the calculated mass.

Infrared spectra

Infrared spectra were recorded using a Bruker Tensor 27 FT-IR spectrometer as neat samples on the ATR diamond module. Absorption maxima (ν_{max}) are reported in wavenumbers (cm^{-1}) and are described as strong (s), medium (m), weak (w), and broad (br).

Melting points

Melting points were measured using a Griffin MFB-700-010U melting point apparatus and are uncorrected.

Reporting of compounds

Systematic names were generated by the computer programme ChemDraw according to the guidelines specified by the International Union of Pure and Applied Chemistry (IUPAC). However, the numbering on the structures may not correspond to the systematic name. The NMR assignments follow the numbering system shown on the structures for straightforward comparison of data.

2. Compound Synthesis and Characterisation

Dimethyl 2-(2,2-dimethylpropylidene)malonate, 32

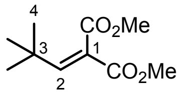

According to the modified procedure of Sakai,[1] pivaldehyde (4.4 mL, 40 mmol), dimethyl malonate (9.2 mL, 80 mmol), and acetic anhydride (3.8 mmol, 40 mmol) were added to a suspension of $InCl_3$ (1.76 g, 8.00 mmol) in dry toluene (20.0 mL). The resulting white suspension was allowed to stir in an oil bath at 80 °C for 16 h. After this time, the reaction mixture was allowed to cool down and was quenched with a mixture of sat. aq. $NaHCO_3$ (150 mL) and brine (40 mL). It was then extracted with EtOAc (3 × 150 mL). The combined organic layers were dried (Na_2SO_4) and evaporated *in vacuo* to give a colourless oil. Purification of the crude mixture by flash-chromatography (eluent: 10% to 15% EtOAc in pentane; 300 g SiO_2) provided the *title compound* as a colourless oil (6.16 g, 30.8 mmol, 77%).

R_f 0.50 (20% EtOAc in $PE_{40\text{-}60}$, $KMnO_4$); **^{1}H NMR** (400 MHz, $CDCl_3$) δ 6.91 (s, 1H, C{2}–H), 3.80 (s, 3H, CO_2**Me**), 3.76 (s, 3H, CO_2**Me**’), 1.12 (s, 9H, C{4}–H); **^{13}C NMR** (101 MHz, $CDCl_3$) δ 167.5 (**C**O_2Me), 165.0 (**C**O_2Me’), 156.0 (C{2}), 124.7 (C{1}), 52.6 (CO_2**Me**), 52.4 (CO_2**Me**’), 34.3 (C{3}), 28.9 (C{4}); **MS** (ESI) m/z: $[M+Na]^+$ Calcd for $C_{10}H_{16}NaO_4^+$ 223.1; Found 223.0. All data in accordance with the literature.[1]

Dimethyl 2-(2,2-dimethylhex-5-en-3-yl)malonate, 42

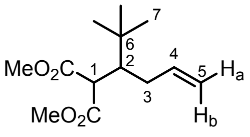

According to the modified procedure of Sakurai,[2] $TiCl_4$ (3.8 mL, 35 mmol) was added dropwise to a solution of dimethyl 2-(2,2-dimethylpropylidene)malonate **32** (6.15 g, 30.7 mmol) in CH_2Cl_2 (190 mL) at −78 °C. The resulting yellow solution was allowed to stir for 5 min before the dropwise addition of allyltrimethylsilane (5.9 mL, 37 mmol). The deep purple solution was then allowed to stir at 0 °C for 2.5 h before being quenched with water (250 mL). The layers

were separated, and the aqueous phase extracted with Et_2O (2 × 250 mL). The combined organic layers were washed with water (3 × 200 mL), dried ($MgSO_4$) and evaporated *in vacuo* to give the *title compound* as a yellow oil (7.19 g, 29.7 mmol, 97%) with no need for further purification.

R*f* 0.46 (25% Et_2O in PE_{40-60}, $KMnO_4$); **^{1}H NMR** (400 MHz, $CDCl_3$) δ 5.75 (dddd, 1H, *J* = 17.0, 10.0, 7.5, 6.0 Hz, C{4}–H), 5.02 (dddd, 1H, *J* = 17.0, 1.5, 1.5, 1.5 Hz, C{5}–H_b), 4.92 (dddd, 1H, *J* = 10.0, 1.5, 1.5, 1.5 Hz, C{5}–H_a), 3.68 (s, 6H, 2 × CO_2**Me**), 3.61 (d, 1H, *J* = 4.5 Hz, C{1}–H), 2.55–2.42 (m, 1H, C{3}–H), 2.36–2.26 (m, 1H, C{3'}–H), 2.22 (ddd, 1H, *J* = 8.5, 4.5, 4.0 Hz, C{2}–H), 0.91 (s, 9H, C{7}–H); **^{13}C NMR** (101 MHz, $CDCl_3$) δ 170.9 (**C**O_2Me), 169.9 (**C**O_2Me'), 139.0 (C{4}), 115.5 (C{5}), 52.5 (CO_2**Me**), 52.1 (CO_2**Me**'), 51.6 (C{1}), 48.9 (C{2}), 34.4 (C{6}), 31.9 (C{3}), 28.1 (C{7}); ***ν*/cm^{-1}** (film) 2954w, 1734s, 1435m, 1147s, 913w; **HRMS** (ESI) m/z: $[M+H]^+$ Calcd for $C_{13}H_{23}O_4^+$ 243.1591; Found 243.1592.

Dimethyl 2-(4,4-dimethyl-1-oxopentan-3-yl)malonate, 24

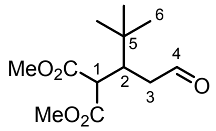

According to the modified procedure of Jin,[3] 2,6-lutidine (5.3 mL, 46 mmol), $K_2OsO_4 \cdot 2H_2O$ (39 mg, 0.11 mmol), and $NaIO_4$ (18.8 g, 87.9 mmol) were added to a solution of dimethyl 2-(2,2-dimethylhex-5-en-3-yl)malonate **42** (5.29 g, 21.8 mmol) in THF/H_2O (4:1, 87 mL) at 0 °C. The resulting mixture was allowed to stir at this temperature for 15 min and at room temperature for 3 h. After this time, the reaction was partitioned between water (300 mL) and CH_2Cl_2 (300 mL). The layers were separated, and the aqueous extracted with CH_2Cl_2 (2 × 300 mL). The combined organic layers were washed with aq. sat. $CuSO_4$ (300 mL), water (300 mL), dried (Na_2SO_4) and evaporated *in vacuo* to give a grey oil. Purification of the crude mixture by flash-chromatography (eluent: 10% to 20% EtOAc in pentane; 250 g SiO_2) provided the *title compound* as a grey oil (4.27 g, 17.5 mmol, 80%).

R$_f$ 0.33 (20% EtOAc in pentane, $KMnO_4$); ^{1}**H NMR** (400 MHz, C_6D_6) δ 9.53 (t, 1H, J = 1.5 Hz, C{4}–H), 3.70 (d, 1H, J = 3.5 Hz, C{1}–H), 3.29 (s, 3H, CO_2**Me**), 3.28 (s, 3H, CO_2**Me**'), 3.01 (ddd, 1H, J = 18.5, 5.5, 1.5 Hz, C{3}–H), 2.85 (ddd, 1H, J = 5.5, 5.5, 3.5 Hz, C{2}–H), 2.30 (ddd, 1H, J = 18.5, 5.5, 1.5 Hz, C{3'}–H), 0.69 (s, 9H, C{6}–H); ^{13}**C NMR** (101 MHz, C_6D_6) δ 200.3 (C{4}), 169.9 (**C**O_2Me), 169.5 (**C**O_2Me'), 52.3 (CO_2**Me**), 51.8 (CO_2**Me**'), 51.4 (C{1}), 42.5 (C{3}), 42.1 (C{2}), 33.8 (C{5}), 27.7 (C{6}); ***v*/cm**$^{-1}$ (film) 2957w, 1730s, 1203m, 1150s; **HRMS** (ESI) m/z: $[M+Na]^+$ Calcd for $C_{12}H_{20}O_5Na^+$ 267.1203; Found 267.1201.

Methyl hept-6-en-2-ynoate, 33

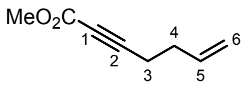

According to the modified procedure of Hopf,[4] propargyl chloride (6.6 mL, 90 mmol) was added over 10 min to a solution of allylmagnesium bromide (1 M in Et_2O, 180 mL, 180 mmol) at −10 °C. The resulting mixture was allowed to stir at room temperature for 18 h. After this time, the reaction was cooled to 0 °C and methyl chloroformate (21 mL, 272 mmol) was added. The resulting off-white suspension was allowed to stir at room temperature for 6 h. It was then cooled to 0 °C and carefully quenched with sat. aq. NH_4Cl (150 mL). The resulting mixture was diluted with water (100 mL) and extracted with Et_2O (2 × 200 mL). The combined organic layers were dried (Na_2SO_4) and evaporated *in vacuo* to give a yellow oil. Purification of the crude mixture by flash-chromatography (eluent: 10% to 60% CH_2Cl_2 in pentane; 200 g SiO_2) provided the *title compound* as a colourless, volatile oil (3.83 g, 27.7 mmol, 31%).

R$_f$ 0.30 (30% CH_2Cl_2 in pentane, $KMnO_4$); ^{1}**H NMR** (400 MHz, $CDCl_3$) δ 5.83 (ddt, 1H, J = 17.0, 10.0, 6.5 Hz, C{5}–H), 5.15–5.04 (m, 2H, C{6}–H), 3.75 (s, 3H, CO_2**Me**), 2.45–2.39 (m, 2H, C{3}–H), 2.36–2.28 (m, 2H, C{4}–H); ^{13}**C NMR** (101 MHz, $CDCl_3$) δ 154.3 (**C**O_2Me), 135.9 (C{5}), 116.6 (C{6}), 89.0 (C{2}), 73.3 (C{1}), 52.7 (CO_2**Me**), 31.7 (C{4}), 18.6 (C{3}). *Note: MS data not available due to lack of ionisation under ESI.* All data in accordance with the literature.[5]

Methyl (*Z*)-3-iodohepta-2,6-dienoate, 25

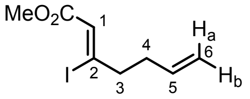

According to the modified procedure of Yun,[6] sodium iodide (10.5 g, 70 mmol) was added to a solution of methyl hept-6-en-2-ynoate **33** (3.83 g, 27.7 mmol) in acetic acid (25 mL). The resulting solution was allowed to stir in an oil bath at 70 °C for 23 h. After this time, the reaction was allowed to cool down to room temperature and was diluted with Et_2O (300 mL) and water (150 mL). The layers were separated, and the organic phase washed with water (3 × 100 mL), sat. aq. $NaHCO_3$ (100 mL), and sat. aq. $Na_2S_2O_3$ (70 mL). This solution was then dried ($MgSO_4$) and evaporated *in vacuo* to give the *title compound* as a brown oil (6.25 g, 23.5 mmol, 85%) with no need for further purification.

R_f 0.48 (10% EtOAc in pentane, $KMnO_4$); **^{1}H NMR** (400 MHz, $CDCl_3$) δ 6.35 (t, 1H, J = 1.0 Hz, C{1}–H), 5.75 (ddt, 1H, J = 17.0, 10.0, 6.5 Hz, C{5}–H), 5.08 (ddt, 1H, J = 17.0, 1.5, 1.5 Hz, C{6}–H_a), 5.03 (ddt, 1H, J = 10.0, 1.5, 1.5 Hz, C{6}–H_b), 3.75 (s, 3H, CO_2**Me**), 2.82–2.75 (m, 2H, C{3}–H), 2.40–2.31 (m, 2H, C{4}–H); **^{13}C NMR** (101 MHz, $CDCl_3$) δ 165.0 (**C**O_2Me), 135.5 (C{5}), 125.0 (C{1}), 120.8 (C{2}), 116.5 (C{6}), 51.7 (CO_2**Me**), 47.3 (C{3}), 33.4 (C{4}); ***v*/cm^{-1}** (film) 2950w, 1732s, 1623m, 1170s, 852w; **HRMS** (ESI) m/z: $[M+H]^+$ Calcd for $C_8H_{12}IO_2^+$ 266.9876; Found 266.9875.

Dimethyl 2-((*R**)-1-((*S**)-3-(but-3-en-1-yl)-5-oxo-2,5-dihydrofuran-2-yl)-3,3-dimethylbutan-2-yl)malonate, 23’

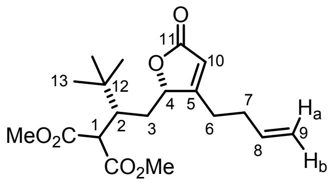

iPrMgCl (2.0 M in THF, 12 mL, 24 mmol) was added over 5 min to a solution of methyl (*Z*)-3-iodohepta-2,6-dienoate **25** (6.25 g, 23.5 mmol) in dry THF (100 mL) at −40 °C. The resulting solution was allowed to stir at this temperature for 1 h. After this time, and in a separate flask,

freshly distilled trimethylsilyl chloride (3.0 mL, 24 mmol) was added to a solution of dimethyl 2-(4,4-dimethyl-1-oxopentan-3-yl)malonate **24** (2.80 g, 11.5 mmol) in THF (60 mL) at −40 °C and was allowed to stir at this temperature for 5 min. After this time, the Grignard reagent solution was transferred to the flask containing aldehyde **24** with the aid of a cannula, while maintaining both solutions at −40 °C. The resulting orange solution was then allowed to stir for 50 min at this temperature. Subsequently, the reaction was quenched through the addition of a solution of acetic acid (15 mL) in THF (20 mL) at −40 °C over 5 min, before it was allowed to warm up to room temperature and was diluted with water (200 mL). This mixture was extracted with EtOAc (3 × 200 mL) and the combined organic layers washed with brine (150 mL), dried (Na_2SO_4) and evaporated *in vacuo* to give a brown oil. Purification of the crude mixture by flash-chromatography (eluent: 5% to 30% EtOAc in pentane; 300 g SiO_2) provided the *title compound* as a colourless oil (3.13 g, 8.89 mmol, 4:1 mixture of inseparable diastereoisomers (major: **23'**), 85%).

Characterised as a 4:1 mixture of diastereoisomers, data for the major diastereoisomer (**23'**): **R_f** 0.40 (30% EtOAc in pentane, $KMnO_4$); **^{1}H NMR** (600 MHz, $CDCl_3$) δ 5.86–5.78 (m, 1H, C{8}–H), 5.76 (ddd, 1H, J = 1.5, 1.5, 1.5 Hz, C{10}–H), 5.11 (dddd, 1H, J = 17.0, 1.5, 1.5, 1.5 Hz, C{9}–H_a), 5.07–5.01 (m, 2H, C{4}–H, C{9}–H_b), 3.83 (d, 1H, J = 2.0 Hz, C{1}–H), 3.77 (s, 3H, CO_2**Me**), 3.74 (s, 3H, CO_2**Me**'), 2.55–2.43 (m, 4H, C{2, 3, 3', 6/7}–H), 2.43–2.31 (m, 3H, C{6, 7}–H), 0.92 (s, 9H, C{13}–H); **^{13}C NMR** (151 MHz, $CDCl_3$) δ 173.7 (C{11}), 173.4 (C{5}), 171.3 (**C**O_2Me), 170.0 (**C**O_2Me'), 136.5 (C{8}), 116.4 (C{9}), 115.3 (C{10}), 83.1 (C{4}), 53.1 (CO_2**Me**), 52.4 (CO_2**Me**'), 50.2 (C{1}), 46.0 (C{2}), 34.3 (C{12}), 31.1 (C{6/7}), 30.9 (C{6/7}), 28.0 (C{13}), 27.5 (C{3}); ***v*/cm^{-1}** (film) 2955w, 1754s, 1436w, 1148m; **HRMS** (ESI) m/z: $[M+Na]^+$ Calcd for $C_{19}H_{28}O_6Na^+$ 375.1778; Found 375.1780.

Dimethyl 2-(1-(3-(but-3-en-1-yl)-5-((triisopropylsilyl)oxy)furan-2-yl)-3,3-dimethyl-butan-2-yl)malonate, 43

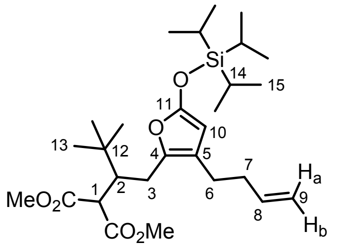

Triethylamine (3.1 mL, 22 mmol) was added to a solution of dimethyl 2-((*R**)-1-((*S**)-3-(but-3-en-1-yl)-5-oxo-2,5-dihydrofuran-2-yl)-3,3-dimethylbutan-2-yl)malonate **23'** (3.13 g, 8.89 mmol, 4:1 d.r.) in CH_2Cl_2 (100 mL) at 0 °C. The resulting solution was allowed to stir at this temperature for 15 min before the addition of triisopropylsilyl trifluoromethanesulfonate (2.7 mL, 10 mmol). The reaction was then allowed to stir at room temperature for 2.5 h before being quenched with water (100 mL) and the resulting layers separated. The aqueous phase was extracted with CH_2Cl_2 (2 × 100 mL) and the combined organic layers were dried (Na_2SO_4) and evaporated *in vacuo* to give a yellow oil. Purification of the crude mixture by flash-chromatography (eluent: 1:5:94 NEt_3/EtOAc/pentane; 125 g SiO_2) provided the *title compound* as a colourless oil (3.84 g, 7.54 mmol, 85%).

$\mathbf{R}_f$ 0.75 (20% EtOAc in pentane, $KMnO_4$); ^{1}**H NMR** (600 MHz, $CDCl_3$) δ 5.84 (dddd, 1H, J = 17.0, 10.0, 6.5, 6.5 Hz, C{8}–H), 5.01 (dddd, 1H, J = 17.0, 1.5, 1.5, 1.5 Hz, C{9}–H_a), 4.94 (dddd, 1H, J = 10.0, 2.0, 1.0, 1.0 Hz, C{9}–H_b), 4.89 (s, 1H, C{10}–H), 3.69 (s, 3H, CO_2**Me**), 3.64 (d, 1H, J = 3.5 Hz, C{1}–H), 3.57 (s, 3H, CO_2**Me'**), 3.02 (dd, 1H, J = 15.5, 8.5 Hz, C{3}–H), 2.65 (dd, 1H, J = 15.5, 4.5 Hz, C{3'}–H), 2.56 (ddd, 1H, J = 8.5, 4.5, 3.5 Hz, C{2}–H), 2.46–2.36 (m, 1H, C{6}–H), 2.36–2.26 (m, 1H, C{6'}–H), 2.24–2.14 (m, 2H, C{7}–H), 1.31–1.18 (m, 3H, C{14}–H), 1.13–1.06 (m, 18H, C{15}–H), 0.93 (s, 9H, C{13}–H); ^{13}**C NMR** (151 MHz, $CDCl_3$) δ 170.2 (**C**O_2Me), 170.1 (**C**O_2Me'), 155.0 (C{11}), 138.8 (C{8}), 138.4 (C{4}), 120.8 (C{5}), 114.8 (C{9}), 85.0 (C{10}), 52.5 (CO_2**Me**), 52.1 (CO_2**Me'**), 51.3 (C{1}), 48.0 (C{2}), 34.4 (C{7}), 34.0 (C{12}), 28.3 (C{13}), 24.8 (C{6}), 23.4 (C{3}), 17.8 (C{15}), 12.4 (C{14}); ***v*/cm**$^{-1}$

(film) 2949m, 1759s, 1737s, 1148s, 860m; **HRMS** (ESI) m/z: $[M+H]^+$ Calcd for $C_{28}H_{49}O_6Si^+$ 509.3293; Found 509.3290.

Dimethyl 2-((*R**)-1-((*R**)-3-(but-3-en-1-yl)-5-oxo-2,5-dihydrofuran-2-yl)-3,3-dimethylbutan-2-yl)malonate, 23

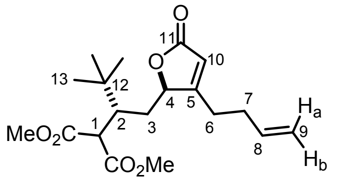

Dimethyl 2-(1-(3-(but-3-en-1-yl)-5-((triisopropylsilyl)oxy)furan-2-yl)-3,3-dimethylbutan-2-yl)-malonate **43** (3.84 g, 7.54 mmol) was dissolved in acetic acid (70 mL) and allowed to stir at room temperature for 1 h. After this time, all volatiles were evaporated *in vacuo* to give a yellow oil. Purification of the crude mixture by flash-chromatography (eluent: 5% to 30% EtOAc in pentane; 250 g SiO_2) provided the *title compound* as a colourless oil (2.31 g, 6.55 mmol, 3:1 mixture of inseparable diastereoisomers (major: **23**), 87%).

Characterised as a 3:1 mixture of diastereoisomers, data for the major diastereoisomer (**23**): **R*f*** 0.40 (30% EtOAc in pentane, $KMnO_4$); ^{1}**H NMR** (600 MHz, $CDCl_3$) δ 5.85–5.77 (m, 1H, C{8}–H), 5.77 (ddd, 1H, J = 1.5, 1.5, 1.5 Hz, C{10}–H), 5.11 (dddd, 1H, J = 17.0, 1.5, 1.5, 1.5 Hz, C{9}–H_a), 5.07 (dddd, 1H, J = 10.0, 1.5, 1.5, 1.5 Hz, C{9}–H_b), 4.77 (ddd, 1H, J = 11.0, 3.0, 1.5 Hz, C{4}–H), 3.81 (s, 3H, CO_2**Me**), 3.75 (d, 1H, J = 3.0 Hz, C{1}–H), 3.70 (s, 3H, CO_2**Me**’), 2.58–2.51 (m, 1H, C{6}–H), 2.41–2.33 (m, 4H, C{2, 6’, 7}–H), 2.11 (ddd, 1H, J = 16.0, 6.5, 3.0 Hz, C{3}–H), 2.01 (ddd, 1H, J = 16.0, 11.0, 3.0 Hz, C{3’}–H), 0.98 (s, 9H, C{13}–H); ^{13}**C NMR** (151 MHz, $CDCl_3$) δ 172.7 (C{11}), 172.4 (C{5}), 170.3 (**C**O_2Me), 169.7 (**C**O_2Me’), 136.2 (C{8}), 116.7 (C{9}), 115.9 (C{10}), 84.8 (C{4}), 53.2 (CO_2**Me**), 52.3 (CO_2**Me**), 51.5 (C{1}), 46.3 (C{2}), 35.0 (C{12}), 31.1 (C{7}), 30.8 (C{3}), 28.3 (C{13}), 27.7 (C{6}); ***v*/cm**$^{-1}$ (film) 2954w, 1756s, 1147w, 857w; **HRMS** (ESI) m/z: $[M+H]^+$ Calcd for $C_{19}H_{29}O_6^+$ 375.1778; Found 375.1780.

Dimethyl 2-(1-(3-(but-3-en-1-yl)-5-oxo-4,5-dihydrofuran-2-yl)-3,3-dimethylbutan-2-yl)malonate, 44

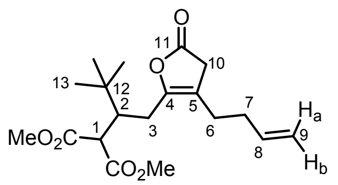

Triethylamine (10 μL, 0.065 mmol) was added to a solution of dimethyl 2-((*R**)-1-((*S**)-3-(but-3-en-1-yl)-5-oxo-2,5-dihydrofuran-2-yl)-3,3-dimethylbutan-2-yl)malonate **23'** (9.0 mg, 0.026 mmol, 4:1 d.r.) in CH_2Cl_2 (1 mL) at 0 °C. The resulting solution was allowed to stir at this temperature for 15 min before the addition of triisopropylsilyl trifluoromethanesulfonate (8 μL, 0.029 mmol). The reaction was then allowed to stir at room temperature for 2.5 h. After this time, the reaction was quenched with trifluoroacetic acid (0.10 mL) and all volatiles were evaporated *in vacuo* to give a yellow oil. Purification of the crude mixture by flash-chromatography (eluent: 0% to 40% EtOAc in pentane; 8 g SiO_2) provided the *title compound* as a colourless oil (2.9 mg, 8.2 μmol, 32%).

$\mathbf{R}_f$ 0.47 (30% EtOAc in pentane, $KMnO_4$); ^{1}**H NMR** (600 MHz, $CDCl_3$) δ 5.79 (dddd, 1H, *J* = 16.5, 10.0, 6.5, 6.5 Hz, C{8}–H), 5.05 (d, 1H, *J* = 16.5 Hz, C{9}–H_a), 5.00 (d, 1H, *J* = 10.0 Hz, C{9}–H_b), 3.73 (s, 3H, CO_2**Me**), 3.72 (d, 1H, *J* = 3.0 Hz, C{1}–H), 3.65 (s, 3H, CO_2**Me**'), 3.14 (dd, 1H, *J* = 15.0, 8.5 Hz, C{3}–H), 3.12–3.00 (m, 2H, C{10}–H), 2.49 (ddd, 1H, *J* = 9.0, 3.5, 3.0 Hz, C{2}–H), 2.44 (ddd, 1H, *J* = 15.0, 2.5, 2.5 Hz, C{3'}–H), 2.41–2.25 (m, 1H, C{6}–H), 2.26–2.15 (m, 2H, C{6', 7}–H), 2.15–2.08 (m, 1H, C{7'}–H), 0.96 (s, 9H, C{13}–H); ^{13}**C NMR** (151 MHz, $CDCl_3$) δ 176.3 (C{11}), 170.2 (**C**O_2Me), 169.7 (**C**O_2Me'), 149.1 (C{4}), 137.8 (C{8}), 115.7 (C{9}), 113.4 (C{5}), 52.6 (CO_2**Me**), 52.3 (CO_2**Me**'), 50.5 (C{1}), 45.7 (C{2}), 36.3 (C{10}), 34.2 (C{12}), 32.4 (C{7}), 28.2 (C{13}), 25.1 (C{6}), 22.8 (C{3}); ***v*/cm**$^{-1}$ (film) 2955w, 1797s, 1756s, 1734s, 1148m; **HRMS** (ESI) m/z: $[M+Na]^+$ Calcd for $C_{19}H_{28}O_6Na^+$ 375.1778; Found 375.1780.

Dimethyl (2*S,3a*R**,5a*R**,8a*S**)-2-(*tert*-butyl)-6-methylene-5-oxohexa-hydro-5*H*-dicyclopenta[*b,c*]furan-1,1(2*H*)-dicarboxylate, 22**

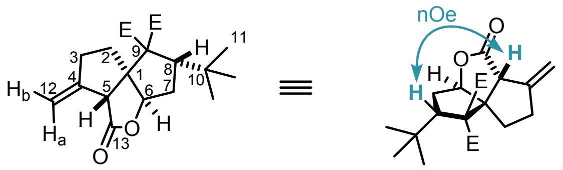

E = CO_2Me E = CO_2Me

$Mn(OAc)_3·2H_2O$ (4.01 g, 15.0 mmol) and $Cu(OAc)_2·H_2O$ (1.48 g, 7.41 mmol) were added to a solution of dimethyl 2-((*R**)-1-((*R**)-3-(but-3-en-1-yl)-5-oxo-2,5-dihydrofuran-2-yl)-3,3-dimethylbutan-2-yl)malonate **23** (2.30 g, 6.53 mmol, 3:1 d.r.) in dry degassed (Ar bubbling, 2 h) DMSO (26 mL) at room temperature. The resulting brown solution was allowed to stir in an oil bath at 60 °C for 18 h, when it had turned deep green. This solution was allowed to cool to room temperature and was quenched with sat. aq. $Na_2S_2O_3$ (50 mL). The resulting mixture was extracted with EtOAc (3 × 50 mL). The combined organic layers were washed with water (3 × 50 mL), brine (100 mL), dried (Na_2SO_4), and evaporated *in vacuo*. Purification of the crude mixture by flash-chromatography (eluent: 20% EtOAc in pentane; 150 g SiO_2) provided the *title compound* as a white solid (1.13 g, 3.23 mmol, 50%).

R*f* 0.50 (30% EtOAc in pentane, $KMnO_4$); **m.p.** 90.7–95.8 °C (from EtOAc); ^{1}**H NMR** (600 MHz, $CDCl_3$) δ 5.06 (br, 1H, C{12}–H_a), 5.00 (br, 1H, C{12}–H_b), 4.90 (d, 1H, J = 8.0 Hz, C{6}–H), 3.75 (s, 3H, CO_2**Me**), 3.67 (s, 3H, CO_2**Me'**), 3.23 (s, 1H, C{5}–H), 2.75 (dd, 1H, J = 14.0, 7.5 Hz, C{8}–H), 2.40 (ddd, 1H, J = 14.5, 13.5, 8.0 Hz, C{7}–H), 2.33–2.17 (m, 4H, C{2, 3}–H), 2.01 (ddd, 1H, J = 14.5, 7.5, 1.0 Hz, C{7'}–H), 0.90 (s, 9H, C{11}–H); ^{13}**C NMR** (151 MHz, $CDCl_3$) δ 176.0 (C{13}), 170.1 (**C**O_2Me), 169.9 (**C**O_2Me'), 146.8 (C{4}), 110.2 (C{12}), 89.7 (C{6}), 68.8 (C{1/9}), 65.2 (C{1/9}), 55.9 (C{5}), 52.7 (C{8}), 52.2 (CO_2**Me**), 52.1 (CO_2**Me'**), 36.0 (C{2}), 33.8 (C{7}), 32.9 (C{10}), 31.9 (C{3}), 28.7 (C{11}); ***v*/cm**$^{-1}$ (film) 2955w, 1773s, 1734s, 1266m, 1201m; **HRMS** (ESI) m/z: $[M+H]^+$ Calcd for $C_{19}H_{27}O_6^+$ 351.1802; Found 351.1801.

Dimethyl (2*S,3a*S**,5a*S**,8a*R**)-2-(*tert*-butyl)-6-methylene-5-oxohexa-hydro-5*H*-dicyclopenta[*b,c*]furan-1,1(2*H*)-dicarboxylate, 34**

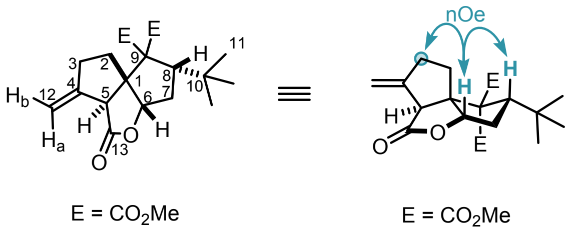

Mn(OAc)$_3$·2H$_2$O (70 mg, 0.25 mmol) and Cu(OAc)$_2$·H$_2$O (26 mg, 0.13 mmol) were added to a solution of dimethyl 2-((*R**)-1-((*S**)-3-(but-3-en-1-yl)-5-oxo-2,5-dihydrofuran-2-yl)-3,3-dimethylbutan-2-yl)malonate **23'** (40 mg, 0.11 mmol, 4:1 d.r.) in dry degassed (Ar bubbling, 2 h) DMSO (0.40 mL) at room temperature. The resulting brown solution was allowed to stir in an oil bath at 60 °C for 36 h, when it had turned deep green. This solution was allowed to cool to room temperature and was quenched with sat. aq. Na$_2$S$_2$O$_3$ (5 mL). The resulting mixture was extracted with EtOAc (3 × 5 mL). The combined organic layers were washed with water (3 × 5 mL), brine (10 mL), dried (Na$_2$SO$_4$), and evaporated *in vacuo*. Purification of the crude mixture by flash-chromatography (eluent: 0% to 30% EtOAc in pentane; 7 g SiO$_2$) provided the *title compound* as a colourless oil (5 mg, 0.014 mmol, *ca.* 13%). The *title compound* exhibited partial decomposition on silica gel, preventing its isolation in high purity.

R*f* 0.40 (30% EtOAc in pentane, KMnO$_4$); ^{1}**H NMR** (600 MHz, CDCl$_3$) *δ* 5.25 (br, 1H, C{12}–H$_a$), 5.07 (br, 1H, C{12}–H$_b$), 4.42 (dd, 1H, *J* = 8.0, 5.5 Hz, C{6}–H), 3.73 (s, 3H, CO$_2$**Me**), 3.73 (s, 3H, CO$_2$**Me**'), 3.32 (ddd, 1H, *J* = 2.0, 2.0, 1.5 Hz, C{5}–H), 2.70 (dd, 1H, *J* = 14.5, 7.0 Hz, C{8}–H), 2.51 (ddd, 1H, *J* = 14.0, 8.5, 7.0 Hz, C{7}–H), 2.46–2.33 (m, 2H, C{3}–H), 2.16 (ddd, 1H, *J* = 14.5, 14.0, 5.5 Hz, C{7'}–H), 1.72 (dd, 1H, *J* = 12.5, 6.5 Hz, C{2}–H), 1.31 (ddd, 1H, *J* = 12.5, 12.5, 7.5 Hz, C{2'}–H), 0.94 (s, 9H, C{11}–H); ^{13}**C NMR** (151 MHz, CDCl$_3$) *δ* 176.8 (C{13}), 170.3 (**C**O$_2$Me), 170.1 (**C**O$_2$Me'), 146.0 (C{4}), 111.2 (C{12}), 86.1 (C{6}), 67.7 (C{9}), 64.9 (C{1}), 55.0 (C{5}), 53.5 (C{8}), 52.5 (CO$_2$**Me**), 52.2 (CO$_2$**Me**'), 35.9 (C{2}), 33.4 (C{7}), 33.0 (C{10}), 31.6 (C{3}), 28.8 (C{11}); ***v*/cm**$^{-1}$ (film) 2958w, 1771s, 1731s, 1241m, 784w; **HRMS** (ESI) m/z: [M+Na]$^+$ Calcd for C$_{19}$H$_{27}$O$_6$$^+$ 351.1802; Found 351.1799.

Dimethyl (2*S,3a*R**,5a*S**,8a*S**)-2-(*tert*-butyl)-5,6-dioxohexahydro-5*H*-dicyclo-penta[*b,c*]furan-1,1(2*H*)-dicarboxylate, 35**

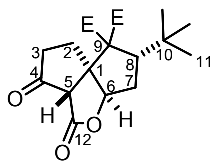

E = CO_2Me

According to the modified procedure of Jin,[3] 2,6-lutidine (0.80 mL, 7.1 mmol), $K_2OsO_4 \cdot 2H_2O$ (115 mg, 0.31 mmol), and $NaIO_4$ (3.53 g, 16.5 mmol) were added to a solution of dimethyl (2*S**,3a*R**,5a*R**,8a*S**)-2-(*tert*-butyl)-6-methylene-5-oxohexahydro-5*H*-dicyclo-penta[*b*,*c*]furan -1,1(2*H*)-dicarboxylate **22** (1.13 g, 3.22 mmol) in 1,4-dioxane/H_2O (3:1, 60 mL) at room temperature. The resulting mixture was allowed to stir at this temperature for 36 h. After this time, the reaction was quenched with aq. sat. $Na_2S_2O_3$ (40 mL) and extracted with EtOAc (3 × 100 mL). The combined organic layers were washed with 0.5 M aq. HCl (100 mL), brine (100 mL), dried (Na_2SO_4) and evaporated *in vacuo* to give a yellow oil. Purification of the crude mixture by flash-chromatography (eluent: 30% to 40% EtOAc in pentane; 100 g SiO_2) provided the *title compound* as a white solid (856 mg, 2.43 mmol, 75%).

R_f 0.51 (50% EtOAc in pentane, $KMnO_4$); **m.p.** 163–164 °C (from EtOAc); **^{1}H NMR** (600 MHz, $CDCl_3$) δ 5.03 (d, 1H, J = 7.5 Hz, C{6}–H), 3.78 (s, 3H, CO_2**Me**), 3.69 (s, 3H, CO_2**Me**'), 3.17 (s, 1H, C{5}–H), 2.70 (dd, 1H, J = 13.5, 7.5 Hz, C{8}–H), 2.51–2.36 (m, 5H, C{2, 2', 3, 3', 7}–H), 2.05 (dd, 1H, J = 15.0, 7.5 Hz, C{7'}–H), 0.92 (s, 9H, C{11}–H); **^{13}C NMR** (151 MHz, $CDCl_3$) δ 204.7 (C{4}), 169.7 (**C**O_2Me), 169.5 (**C**O_2Me'), 168.9 (C{12}), 90.3 (C{6}), 68.9 (C{9}), 62.7 (C{1}), 59.6 (C{5}), 52.7 (C{8}), 52.7 (CO_2**Me**), 52.5 (CO_2**Me**'), 36.8 (C{3}), 33.7 (C{7}), 33.0 (C{10}), 30.7 (C{2}), 28.8 (C{11}); ***v*/cm^{-1}** (film) 2957w, 1787s, 1734s, 1208m; **HRMS** (ESI) m/z: $[M+H]^+$ Calcd for $C_{18}H_{25}O_7^+$ 353.1595; Found 353.1595.

Dimethyl (2*S**,3a*R**,5a*R**,9a*S**)-2-(*tert*-butyl)-5,7-dioxohexahydro-7*H*-cyclopenta[4,5]furo[3,4-*b*]pyran-1,1 (2*H*)-dicarboxylate, 39

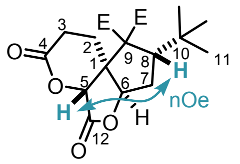

E = CO_2Me

Isolated in variable amounts during the optimisation of the hydroxylation of dimethyl (2*S**,3a*R**,5a*S**,8a*S**)-2-(*tert*-butyl)-5,6-dioxohexahydro-5*H*-dicyclopenta[*b*,*c*]furan-1,1 (2*H*)-dicarboxylate **35** with dimethyldioxirane.

R*f* 0.31 (40% EtOAc in pentane, $KMnO_4$); **^{1}H NMR** (600 MHz, $CDCl_3$) δ 5.09 (s, 1H, C{5}–H), 5.06 (dd, 1H, J = 9.0, 1.5 Hz, C{6}–H), 3.80 (s, 3H, CO_2**Me**), 3.77 (s, 3H, CO_2**Me**'), 2.90 (dd, 1H, J = 13.5, 8.5 Hz, C{8}–H), 2.62–2.55 (m, 1H, C{3}–H), 2.54–2.41 (m, 2H, C{2, 7}–H), 2.34–2.17 (m, 2H, C{2', 3'}–H), 2.03 (ddd, 1H, J = 15.0, 8.5, 1.5 Hz, C{7'}–H), 0.92 (s, 9H, C{11}–H); **^{13}C NMR** (151 MHz, $CDCl_3$) δ 171.1 (C{12}), 169.2 (C{4}/**C**O_2Me), 169.2 (C{4}/**C**O_2Me), 168.9 (C{4}/**C**O_2Me), 88.6 (C{6}), 75.4 (C{5}), 67.4 (C{9}), 55.5 (C{1}), 53.4 (CO_2**Me**), 52.6 (CO_2**Me**'), 52.5 (C{8}), 33.2 (C{10}), 32.9 (C{7}), 28.5 (C{3}), 28.5 (C{11}), 27.2 (C{2}); ***v*/cm^{-1}** (film) 2959w, 1791s, 1737m, 1213s, 1125s; **HRMS** (ESI) m/z: $[M+Na]^+$ Calcd for $C_{18}H_{24}O_8Na^+$ 391.1363; Found 391.1347.

Dimethyl (2*S**,3a*R**,5a*R**,8a*S**)-5a-(((1*S*,5*S*)-9-azabicyclo[3.3.1]nonan-9-yl)oxy)-2-(*tert*-butyl)-5,6-dioxohexahydro-5*H*-dicyclopenta[*b,c*]furan-1,1(2*H*)dicarboxylate, 40

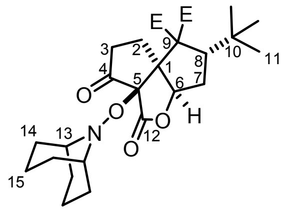

E = CO_2Me

According to the modified procedure of Terent'ev,[7] $Mn(OAc)_3\cdot 2H_2O$ (32 mg, 0.12 mmol) was added to a solution of dimethyl (2*S**,3a*R**,5a*S**,8a*S**)-2-(*tert*-butyl)-5,6-dioxohexahydro-5*H*-

dicyclopenta[*b*,*c*]furan-1,1(2*H*)-dicarboxylate **35** (35 mg, 0.10 mmol) and ABNO (17 mg, 0.12 mmol) in anhydrous acetic acid (1.0 mL). The resulting yellow solution was allowed to stir in an oil bath at 60 °C for 2 h. After this time, the reaction was diluted with water (10 mL) and extracted with EtOAc (3 × 10 mL). The combined organic layers were washed with aq. sat. $Na_2S_2O_3$ (10 mL), brine (10 mL), dried (Na_2SO_4) and evaporated *in vacuo* to give a colourless oil. Purification of the crude mixture by flash-chromatography (eluent: 10% to 40% EtOAc in pentane; 7 g SiO_2) provided the *title compound* as a colourless oil (41 mg, 0.084 mmol, 84%).

R*f* 0.60 (50% EtOAc in $PE_{40\text{-}60}$, $KMnO_4$); **^{1}H NMR** (600 MHz, $CDCl_3$) *δ* 5.07 (dd, 1H, *J* = 9.5, 4.5 Hz, C{6}–H), 3.76 (s, 3H, CO_2**Me**), 3.75 (br, 1H, C{13}–H), 3.58 (s, 3H, CO_2**Me**’), 3.43 (dd, 1H, *J* = 11.5, 9.5 Hz, C{8}–H), 3.22 (br, 1H, C{13’}–H), 2.46 (ddd, 1H, *J* = 15.0, 9.5, 9.5 Hz, C{7}–H), 2.40–2.25 (m, 4H, C{2, 3, 3’, 14}–H), 2.25–2.12 (m, 2H, C{2’, 14’}–H), 2.10–2.00 (m, 3H, C{7’, 14”, 14”’}–H), 1.99–1.88 (m, 1H, C{15}–H), 1.85–1.68 (m, 3H, C{14””, 14””’, 15’}–H), 1.61 (ddd, 1H, *J* = 14.0, 7.0, 7.0 Hz, C{15”}–H), 1.46–1.36 (m, 3H, C{14”””, 14”””’, 15”’}–H), 0.93 (s, 9H, C{11}–H); **^{13}C NMR** (151 MHz, $CDCl_3$) *δ* 205.9 (C{4}), 169.9 (**C**O_2Me), 169.1 (C{12}), 168.6 (**C**O_2Me’), 91.7 (C{5}), 89.9 (C{6}), 67.4 (C{9}), 64.7 (C{1}), 56.0 (C{13}), 55.9 (C{13’}), 53.4 (C{8}), 52.4 (CO_2**Me**), 51.6 (CO_2**Me**’), 34.0 (C{10}), 33.6 (C{7}), 32.6 (C{14}), 32.0 (C{14’}), 32.0 (C{3}), 29.2 (C{11}), 28.5 (C{2}), 24.0 (C{14”}), 23.4 (C{14”’}), 19.9 (C{15}), 19.6 (C{15’}); ***ν*/cm^{-1}** (film) 2951m, 1784s, 1766s, 1734s, 1145m; **HRMS** (ESI) m/z: $[M+H]^+$ Calcd for $C_{26}H_{38}NO_8^+$ 492.2592; Found 492.2570.

Dimethyl (2*S**,3a*R**,5a*S**,8a*S**)-2-(*tert*-butyl)-7-hydroxy-5,6-dioxo-3,3a,5a,6-tetrahydro-5*H*-dicyclopenta[*b,c*]furan-1,1(2*H*)-dicarboxylate, 36

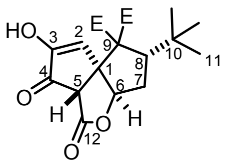

E = CO_2Me

According to the modified procedure of Terent’ev,[7] $Mn(OAc)_3{\cdot}2H_2O$ (5.0 mg, 20 *µ*mol) was added to a solution of dimethyl (2*S**,3a*R**,5a*S**,8a*S**)-2-(*tert*-butyl)-5,6-dioxohexahydro-5*H*-

dicyclopenta[*b*,*c*]furan-1,1(2*H*)-dicarboxylate **35** (3.5 mg, 10 μmol) and TEMPO (3.0 mg, 20 μmol) in anhydrous acetic acid (0.10 mL). The resulting yellow solution was allowed to stir in an oil bath at 60 °C for 3 h. After this time, the reaction was diluted with water (2 mL) and extracted with EtOAc (3 × 3 mL). The combined organic layers were washed with aq. sat. $Na_2S_2O_3$ (4 mL), brine (4 mL), dried (Na_2SO_4) and evaporated *in vacuo* to give a yellow oil. Purification of the crude mixture by flash-chromatography (eluent: 30% to 40% EtOAc in pentane; 1 g SiO_2) provided the *title compound* as a colourless oil (1.7 mg, 4.6 μmol, 46%).

$\mathbf{R}_f$ 0.24 (40% EtOAc in pentane, $KMnO_4$); ^{1}**H NMR** (600 MHz, $CDCl_3$) δ 6.71 (s, 1H, C{2}–H), 5.08 (d, 1H, J = 8.0 Hz, C{6}–H), 3.79 (s, 3H, CO_2**Me**), 3.62 (s, 3H, CO_2**Me**'), 3.42 (s, 1H, C{5}–H), 2.83 (dd, 1H, J = 14.0, 7.5 Hz, C{8}–H), 2.46 (ddd, 1H, J = 15.0, 14.0, 8.0 Hz, C{7}–H), 2.13 (dd, 1H, J = 15.0, 7.5 Hz, C{7'}–H), 0.94 (s, 9H, C{11}–H); ^{13}**C NMR** (151 MHz, $CDCl_3$) δ 191.2 (C{4}), 169.5 (**C**O_2Me), 169.0 (**C**O_2Me'), 167.5 (C{12}), 152.6 (C{3}), 128.8 (C{2}), 85.9 (C{6}), 68.5 (C{9}), 61.9 (C{1}), 54.2 (C{5}), 52.9 (CO_2**Me**), 52.7 (CO_2**Me**'), 52.6 (C{8}), 33.8 (C{7}), 33.2 (C{10}), 28.7 (C{11}); ***ν*/cm**$^{-1}$ (film) 3421br, 2969m, 1783s, 1722s, 1373s; **HRMS** (ESI) m/z: $[M+Na]^+$ Calcd for $C_{18}H_{22}O_8Na^+$ 389.1207; Found 389.1201.

Methyl (1*S**,2*S**,3a*R**,5a*R**,8a*S**)-2-(*tert*-butyl)-5,6,10-trioxotetrahydro-5*H*,6*H*-5a,1-(epoxymethano)dicyclopenta[*b,c*]furan-1(2*H*)-carboxylate, 9

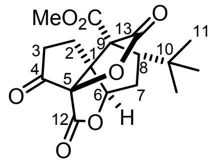

According to the modified procedure of Terent'ev,[7] $Mn(OAc)_3 \cdot 2H_2O$ (32 mg, 0.12 mmol) was added to a solution of dimethyl (2*S**,3a*R**,5a*S**,8a*S**)-2-(*tert*-butyl)-5,6-dioxohexahydro-5*H*-dicyclopenta[*b*,*c*]furan-1,1(2*H*)-dicarboxylate **35** (35 mg, 0.10 mmol) and ABNO (17 mg, 0.12 mmol) in anhydrous acetic acid (1.0 mL). The resulting yellow solution was allowed to stir in an oil bath at 60 °C for 1.5 h. After this time, the reaction mixture was transferred into a separate flask containing Pd (10% w/w on activated carbon, 53 mg, 0.050 mmol) and aq. HCl (2 M, 0.35 mL) in THF (6.5 mL). The atmosphere was then replaced with hydrogen and the

resulting mixture allowed to stir for 18 h at room temperature. After this time, the reaction was filtered through a Celite pad, which was eluted with EtOAc (20 mL). This solution was then washed with water (10 mL), sat. aq. $NaHCO_3$ (10 mL), and brine (10 mL). It was then dried (Na_2SO_4) and evaporated *in vacuo* to give a yellow oil. This oil was dissolved in 40% EtOAc in pentane (6 mL), SiO_2 (1 g) added, and the resulting mixture allowed to stand at room temperature for 2.5 h. After this time, all volatiles were evaporated *in vacuo* and the crude mixture purified by flash-chromatography (50% to 100% EtOAc in pentane) to give the *title compound* as an off-white solid (21.5 mg, 0.064 mmol, 64%).

R_f 0.29 (60% EtOAc in pentane, $KMnO_4$); **m.p.** 226–232 °C (from EtOAc); **^{1}H NMR** (600 MHz, $CDCl_3$) δ 4.99 (d, 1H, J = 5.0 Hz, C{6}–H), 3.83 (s, 3H, CO_2**Me**), 2.75–2.66 (m, 2H, C{3, 7}–H), 2.59 (ddd, 1H, J = 18.0, 13.5, 8.5 Hz, C{3'}–H), 2.49 (dd, 1H, J = 14.0, 8.5 Hz, C{2}–H), 2.36–2.30 (m, 2H, C{7', 8}–H), 1.84 (ddd, 1H, J = 14.0, 13.5, 8.0 Hz, C{2'}–H), 1.01 (s, 9H, C{11}–H); **^{13}C NMR** (151 MHz, $CDCl_3$) δ 199.5 (C{4}), 168.8 (C{13}), 166.6 (**C**O_2Me/C{12}), 166.5 (**C**O_2Me/C{12}), 87.4 (C{6}), 83.1 (C{5}), 65.9 (C{9}), 64.8 (C{1}), 58.9 (C{8}), 52.9 (CO_2**Me**), 37.4 (C{7}), 37.1 (C{3}), 33.6 (C{10}), 29.2 (C{11}), 27.9 (C{2}); ***v*/cm^{-1}** (film) 2928w, 1821s, 1793s, 1745s, 1012m; **HRMS** (ESI) m/z: $[M+H]^+$ Calcd for $C_{17}H_{21}O_7^+$ 337.1282; Found 337.1270.

3. NMR Spectra

Dimethyl 2-(2,2-dimethylpropylidene)malonate, 32

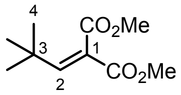

400 MHz ^{1}H NMR spectrum ($CDCl_3$)

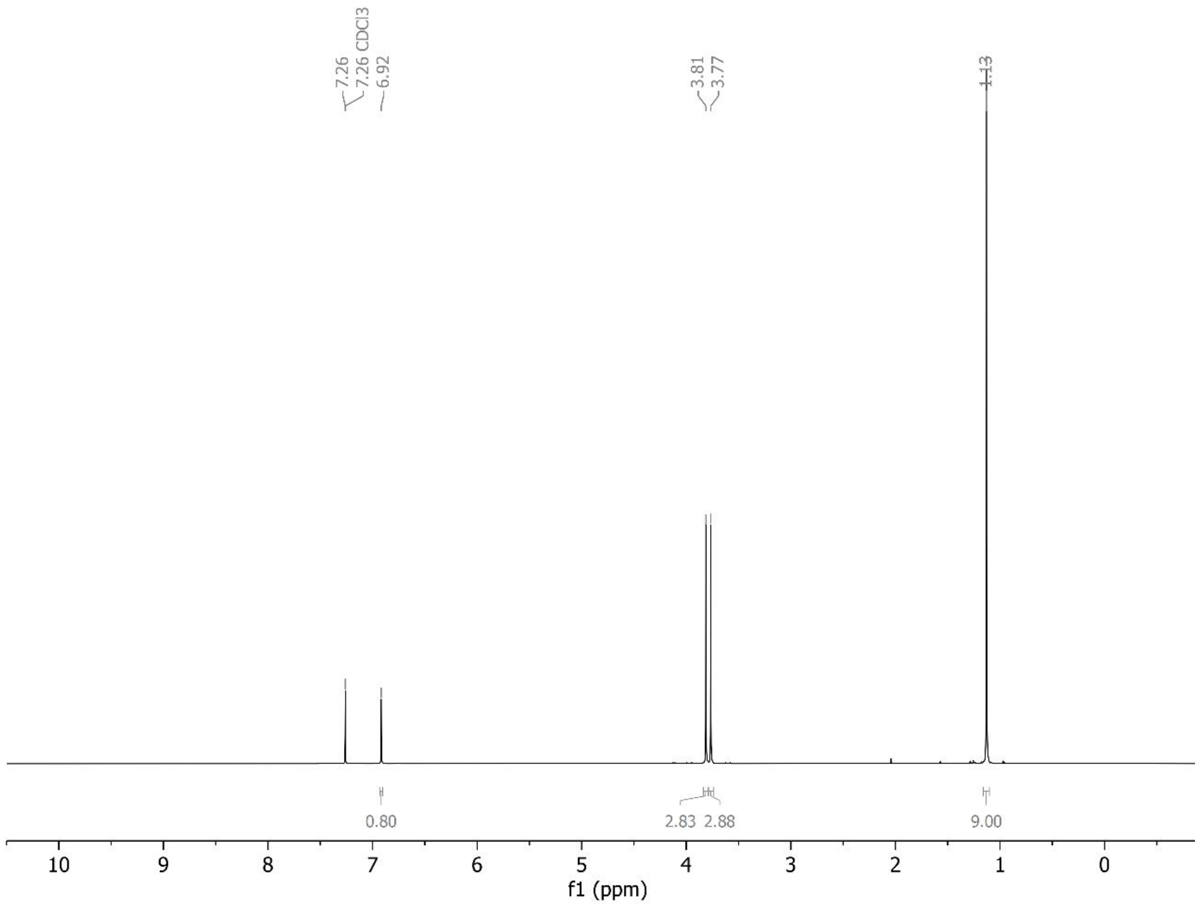

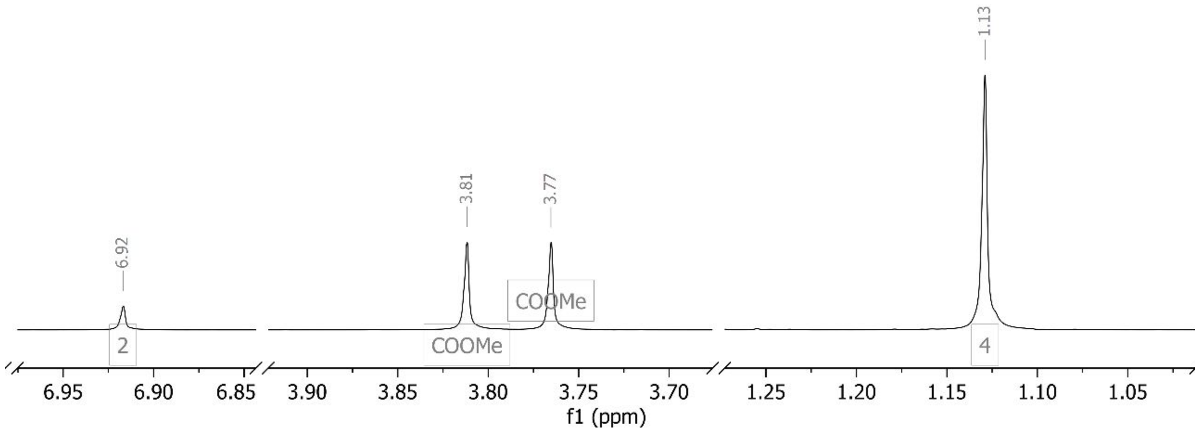

101 MHz ^{13}C NMR spectrum ($CDCl_3$)

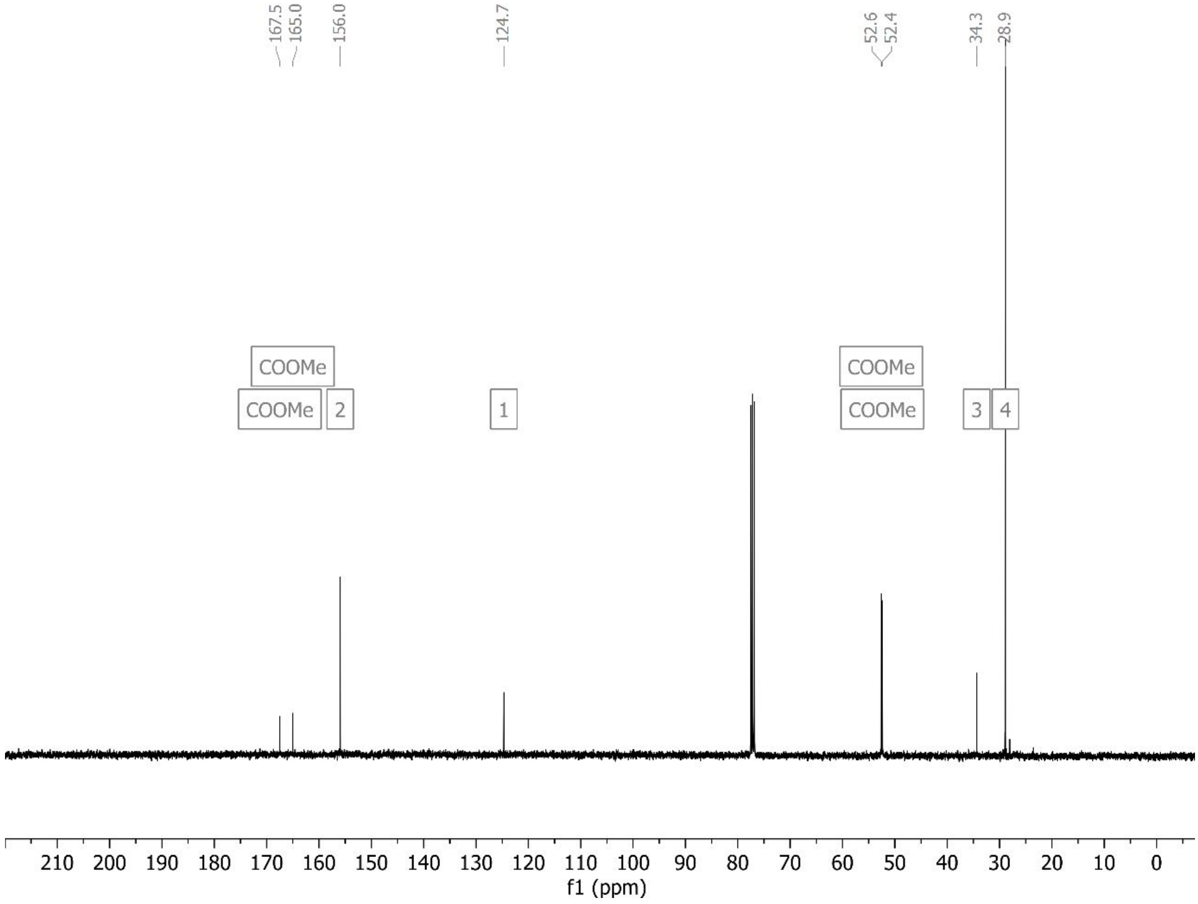

Dimethyl 2-(2,2-dimethylhex-5-en-3-yl)malonate, 42

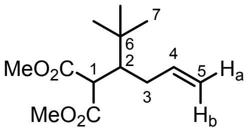

400 MHz ^{1}H NMR spectrum ($CDCl_3$)

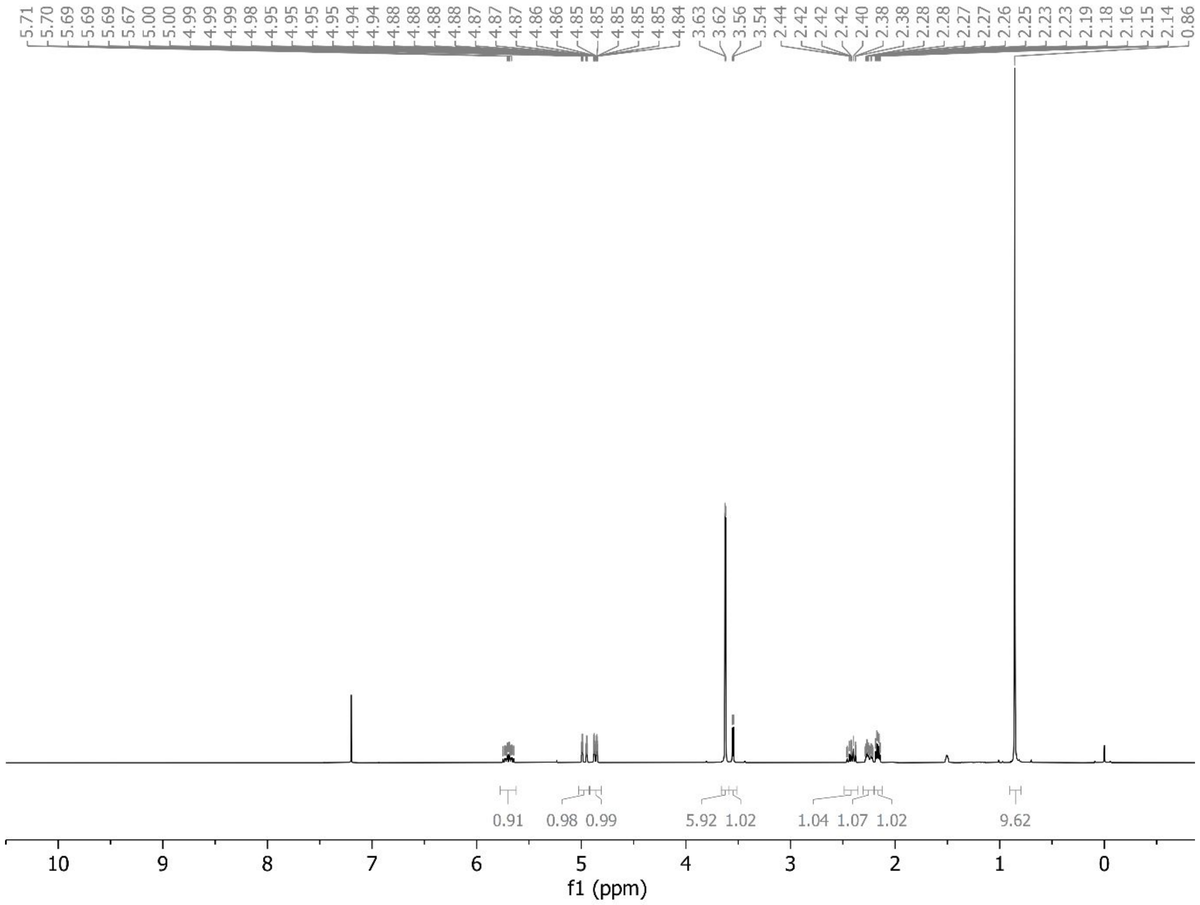

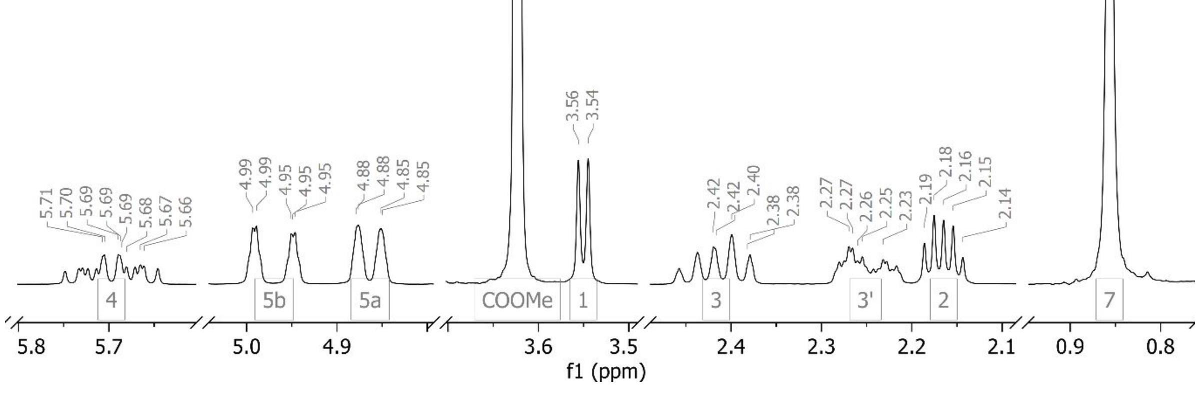

101 MHz ^{13}C NMR spectrum ($CDCl_3$)

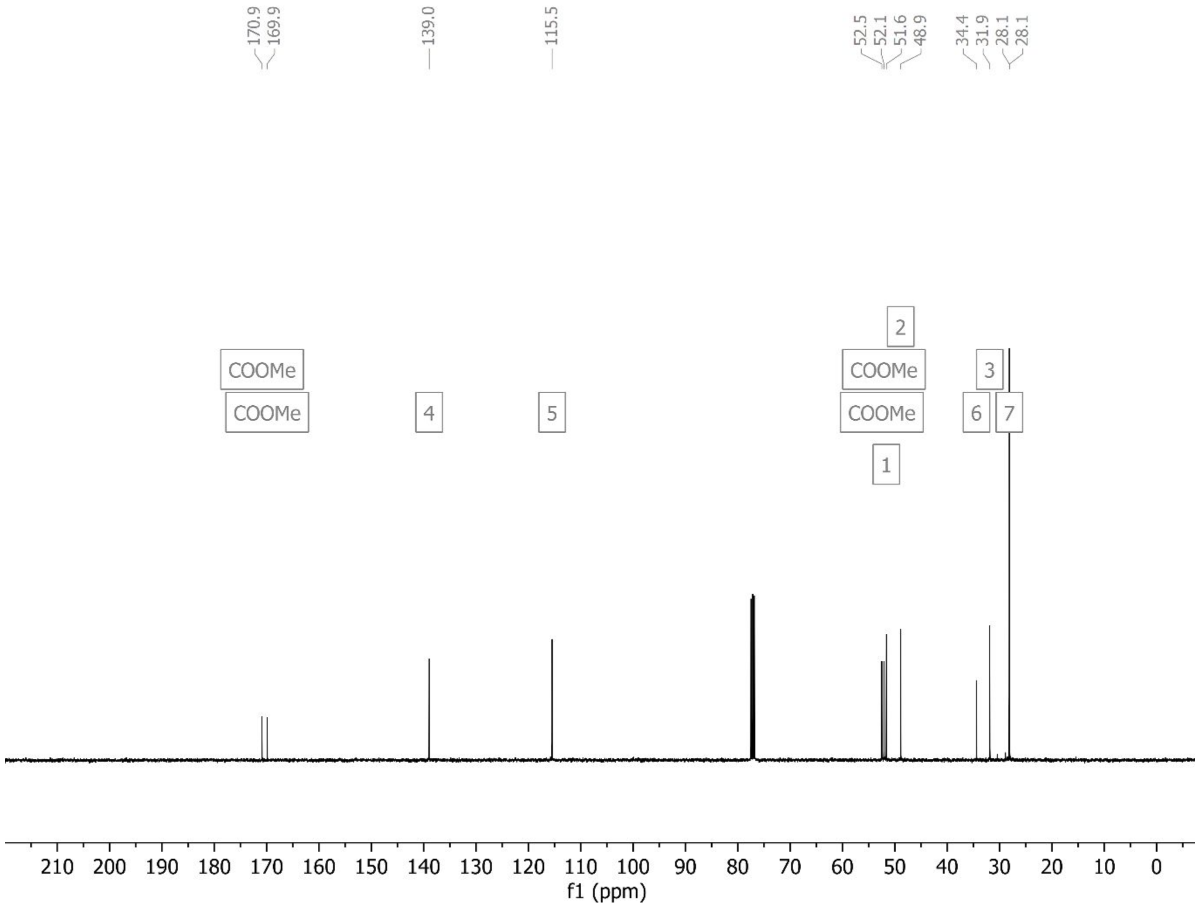

Dimethyl 2-(4,4-dimethyl-1-oxopentan-3-yl)malonate, 24

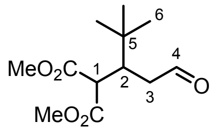

400 MHz ^{1}H NMR spectrum (C_6D_6)

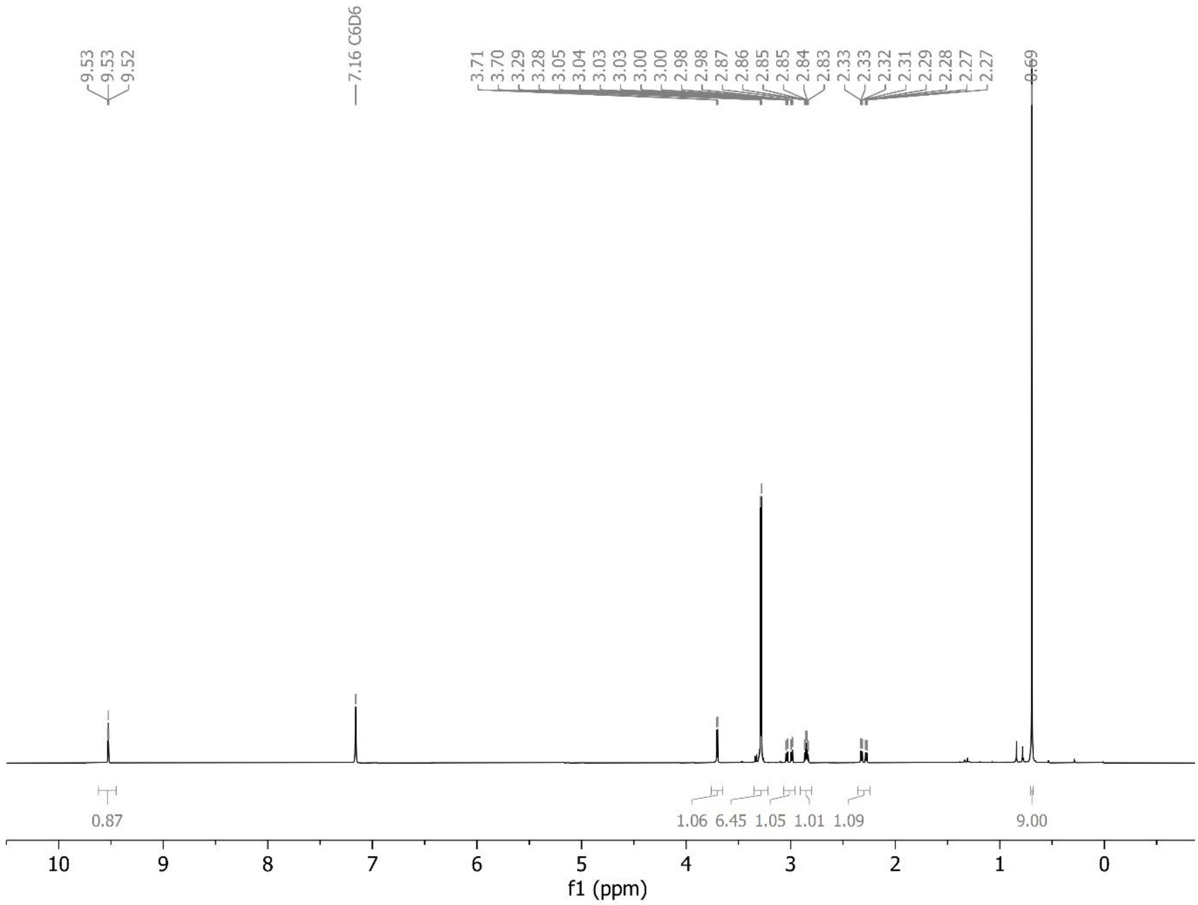

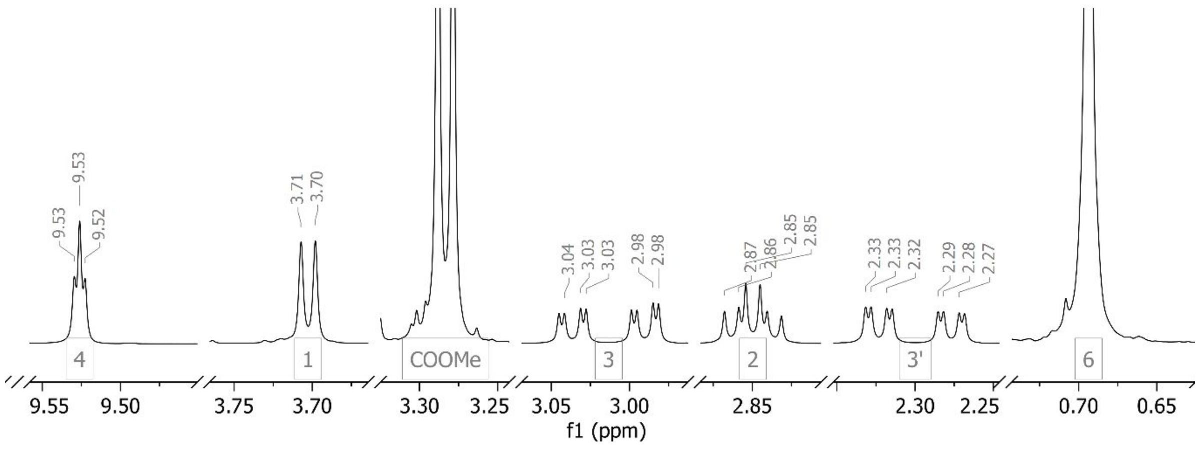

101 MHz ^{13}C NMR spectrum (C_6D_6)

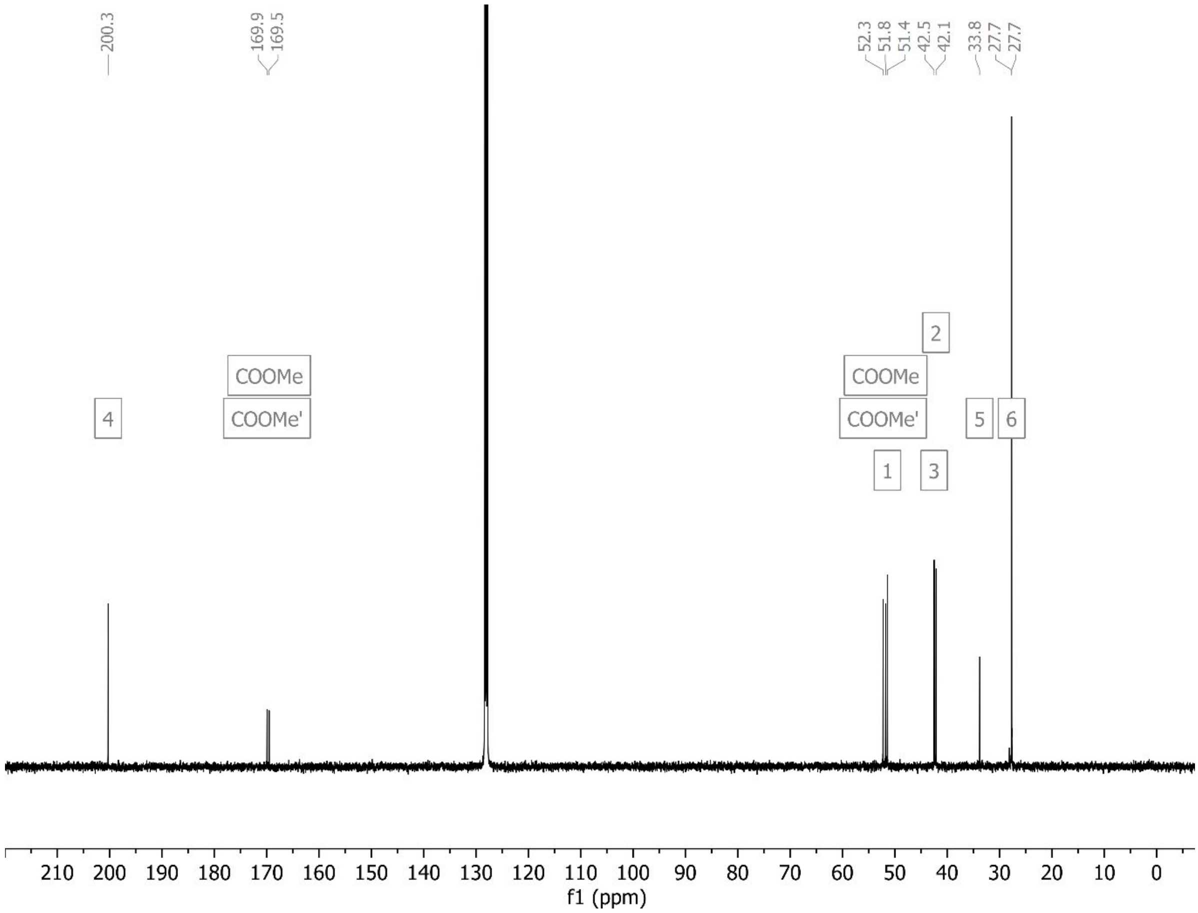

Methyl hept-6-en-2-ynoate, 33

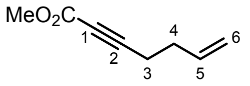

400 MHz ^{1}H NMR spectrum ($CDCl_3$)

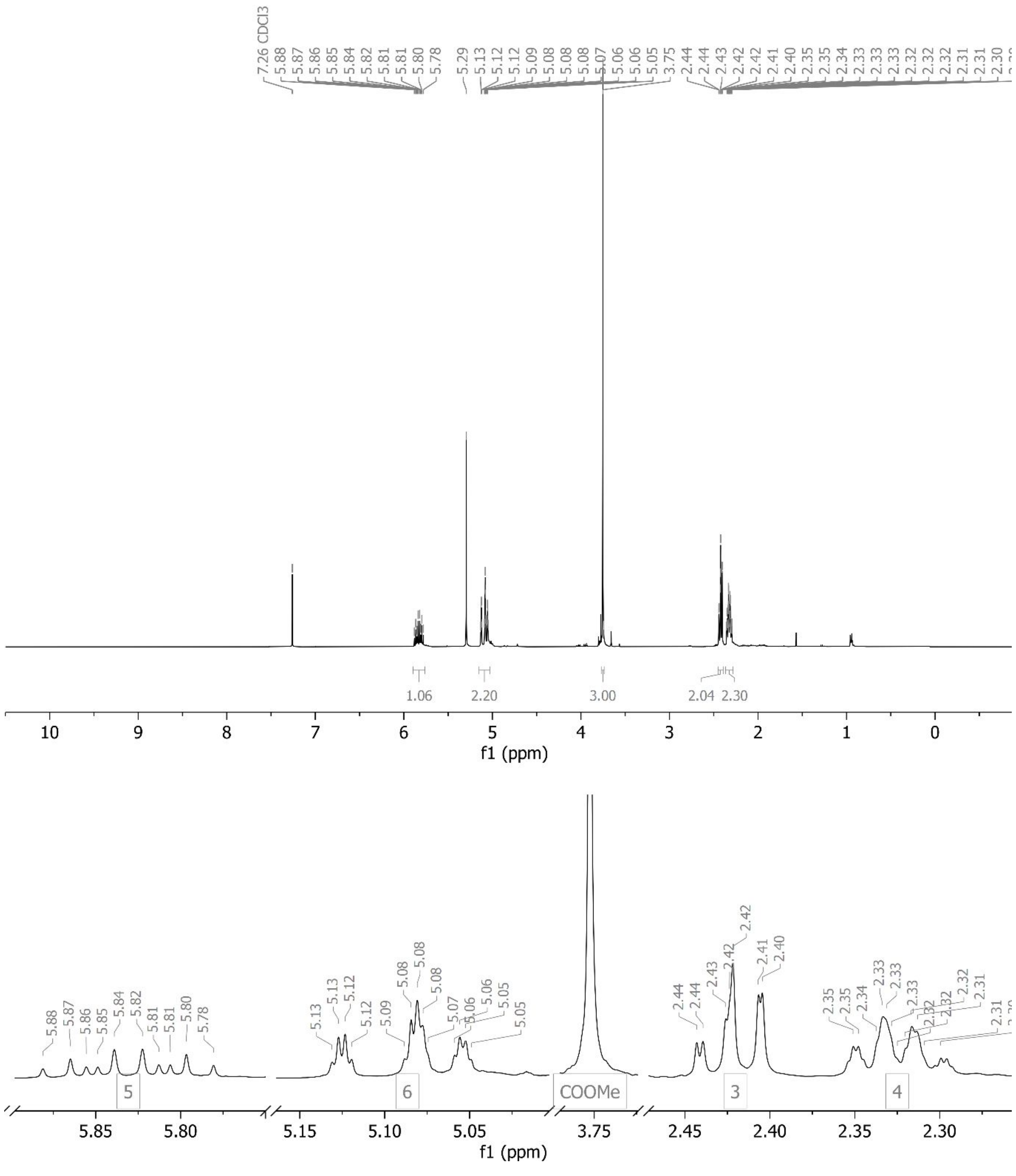

101 MHz ^{13}C NMR spectrum ($CDCl_3$)

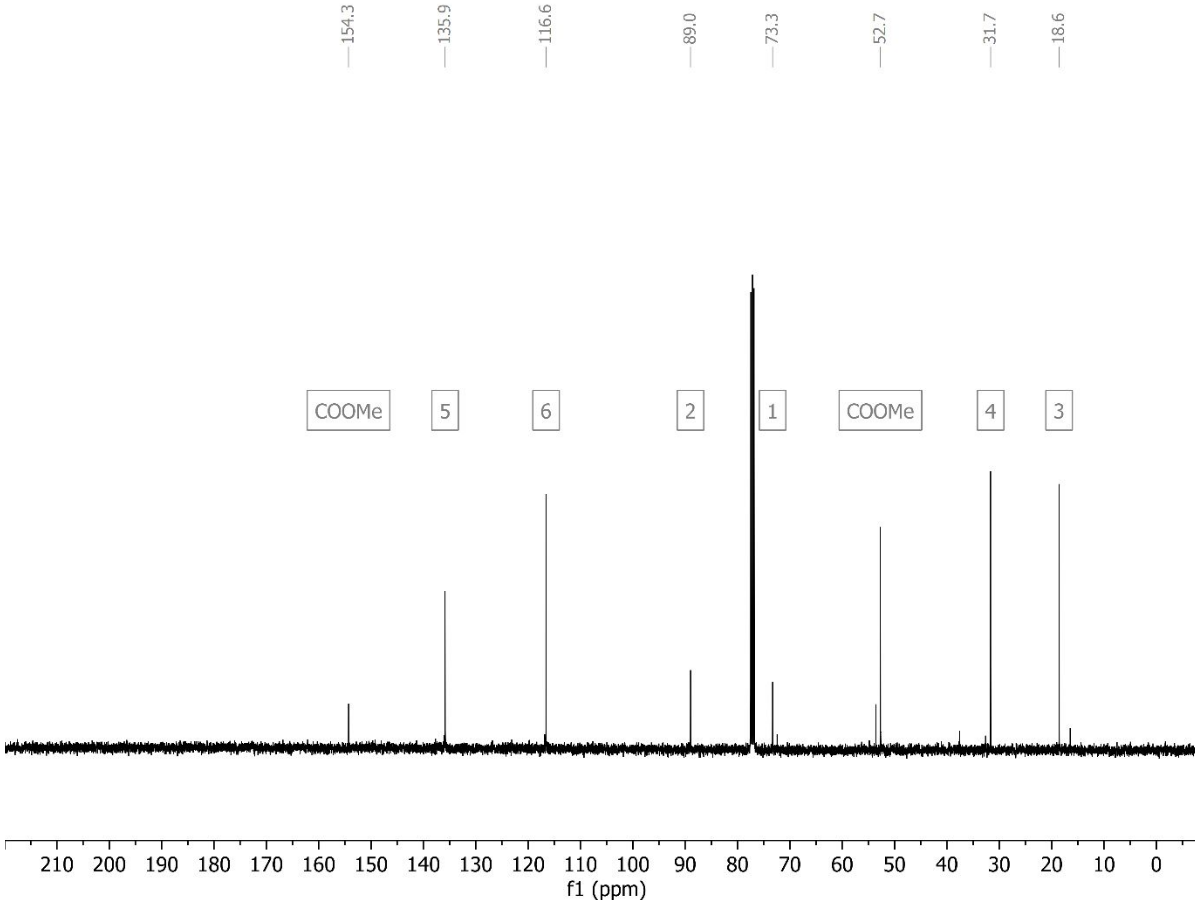

Methyl (*Z*)-3-iodohepta-2,6-dienoate, 25

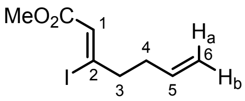

400 MHz ^{1}H NMR spectrum ($CDCl_3$)

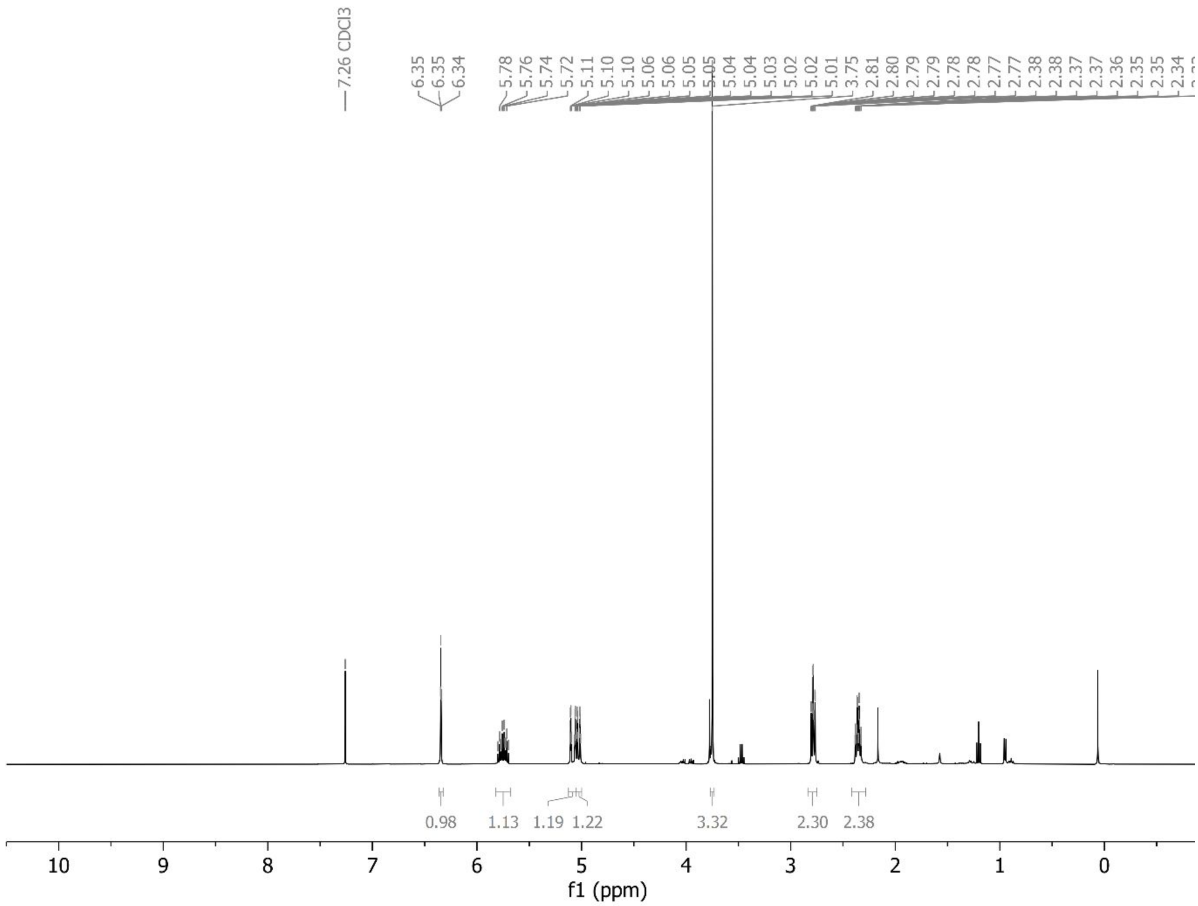

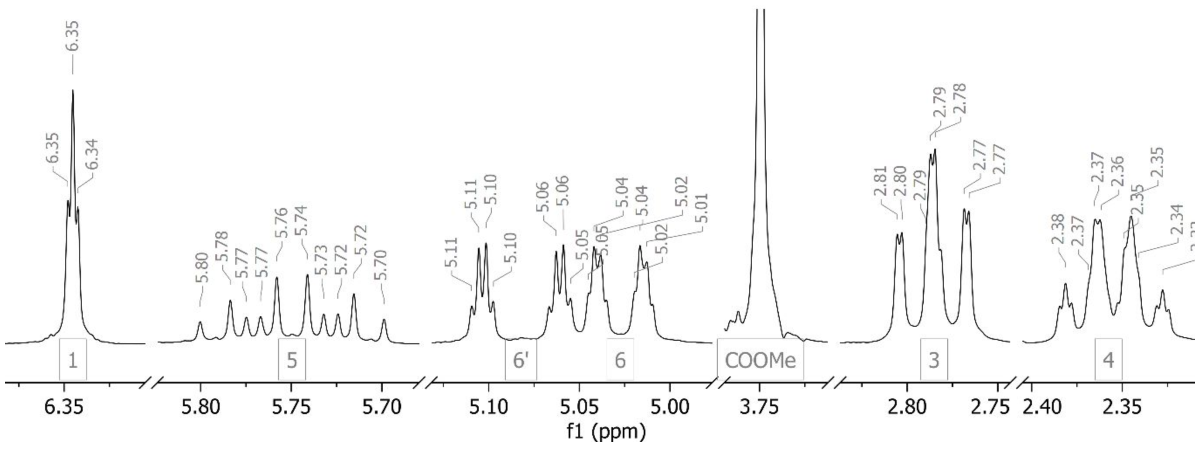

101 MHz ^{13}C NMR spectrum ($CDCl_3$)

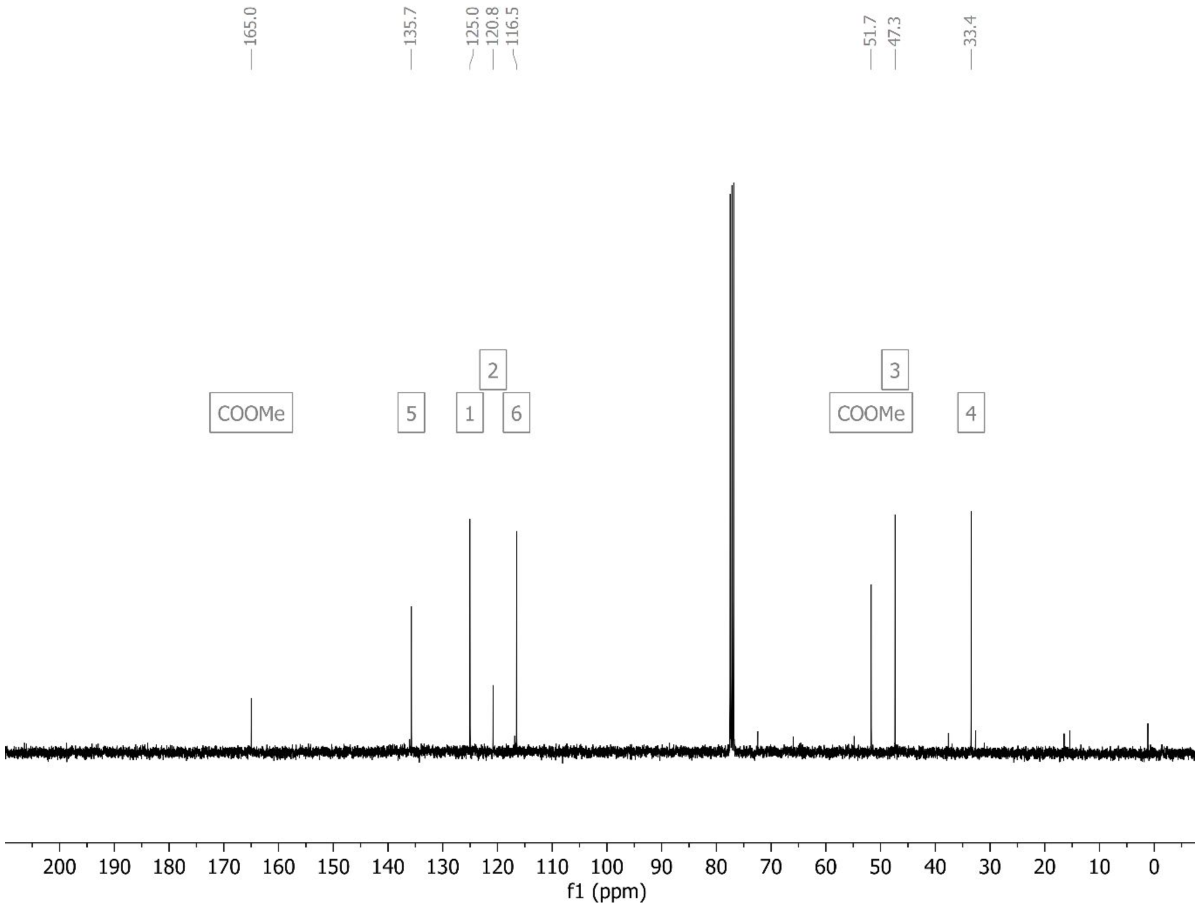

Dimethyl 2-((*R)-1-((*S**)-3-(but-3-en-1-yl)-5-oxo-2,5-dihydrofuran-2-yl)-3,3-dimethylbutan-2-yl)malonate, 23’, 4:1 d.r.**

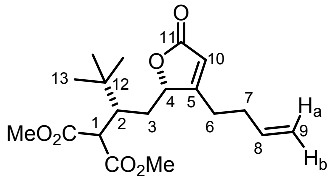

600 MHz ^{1}H NMR spectrum ($CDCl_3$)

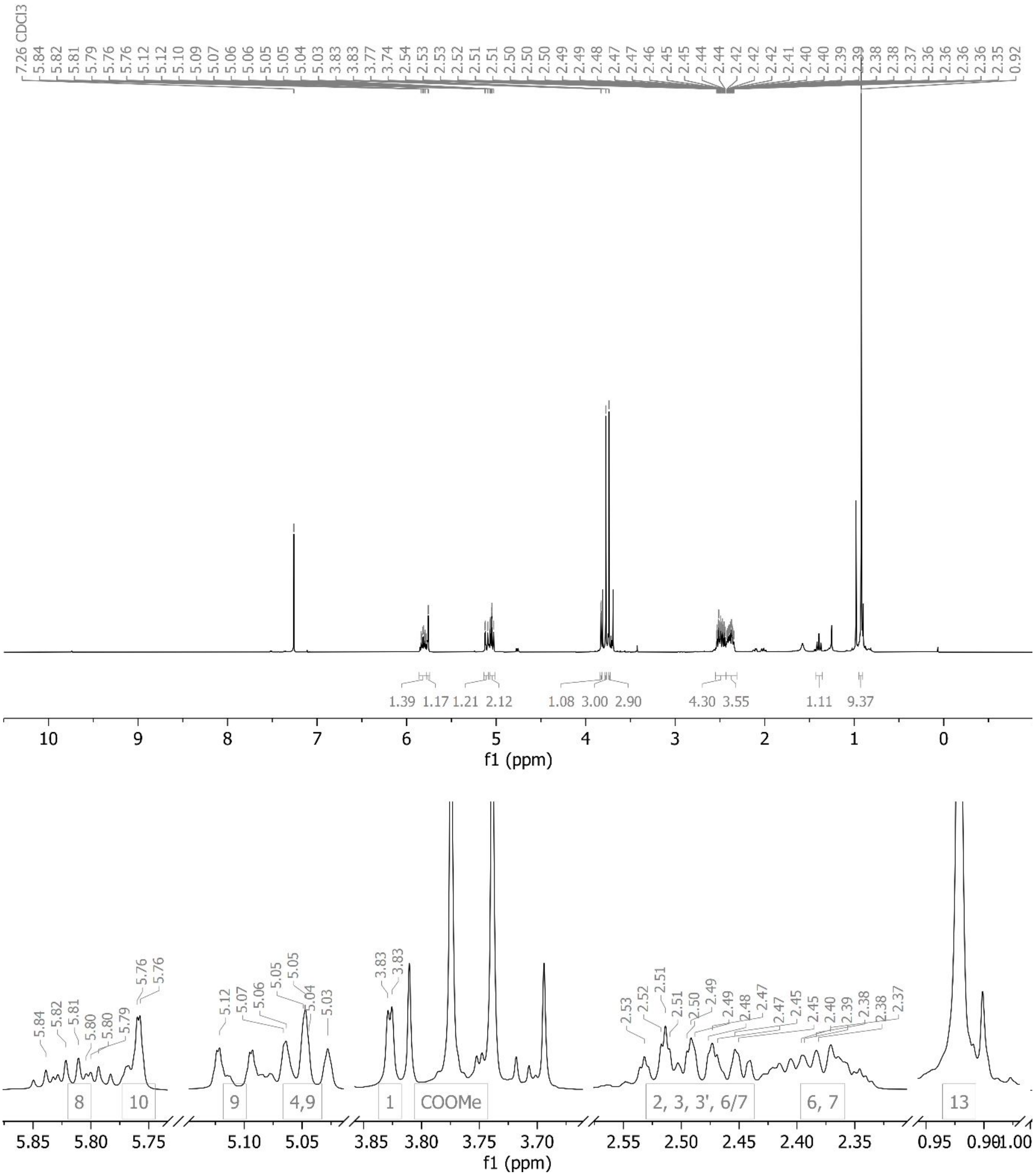

151 MHz ^{13}C NMR spectrum ($CDCl_3$)

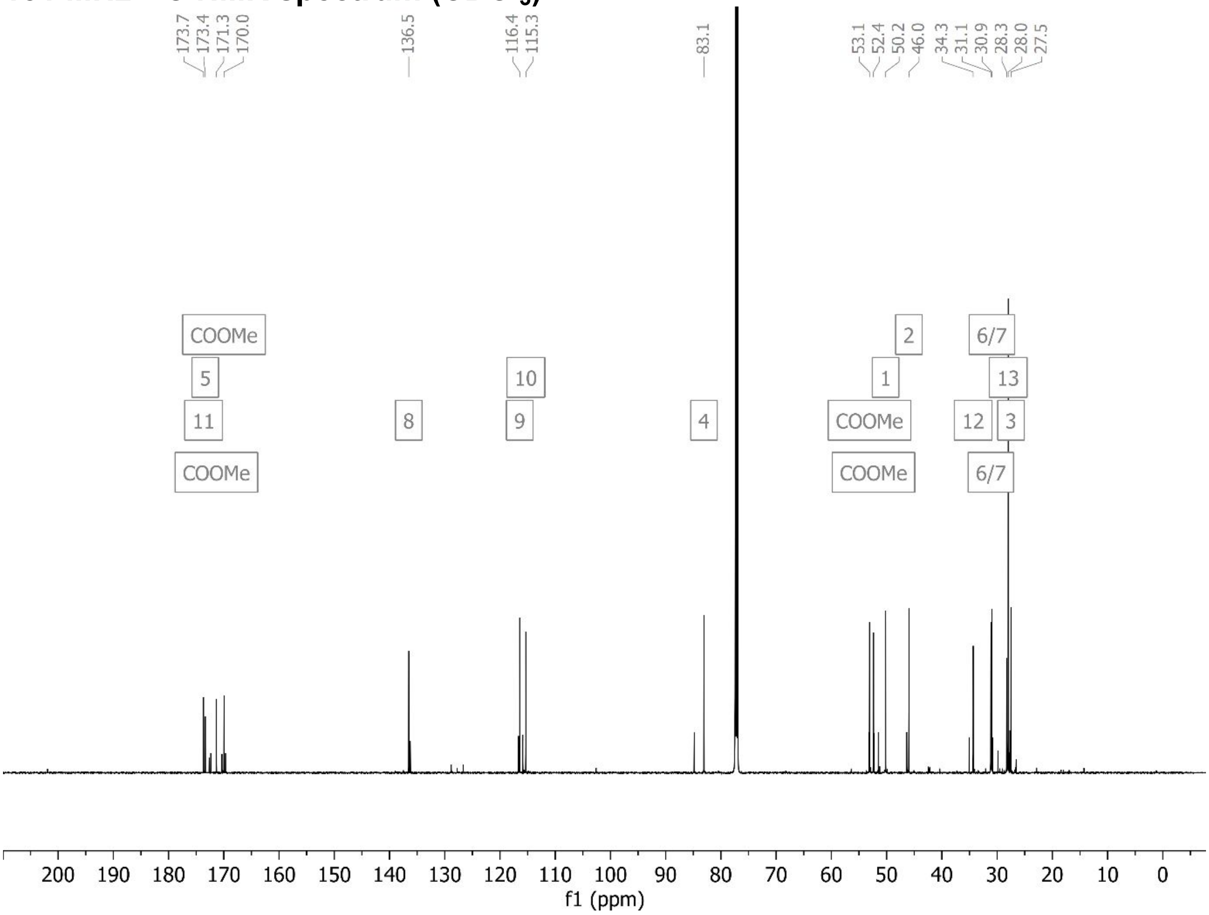

Dimethyl 2-(1-(3-(but-3-en-1-yl)-5-((triisopropylsilyl)oxy)furan-2-yl)-3,3-dimethyl-butan-2-yl)malonate, 43

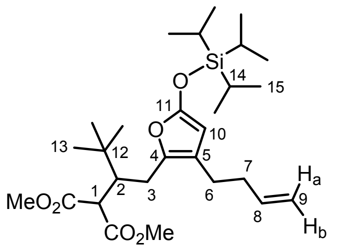

600 MHz ^{1}H NMR spectrum ($CDCl_3$)

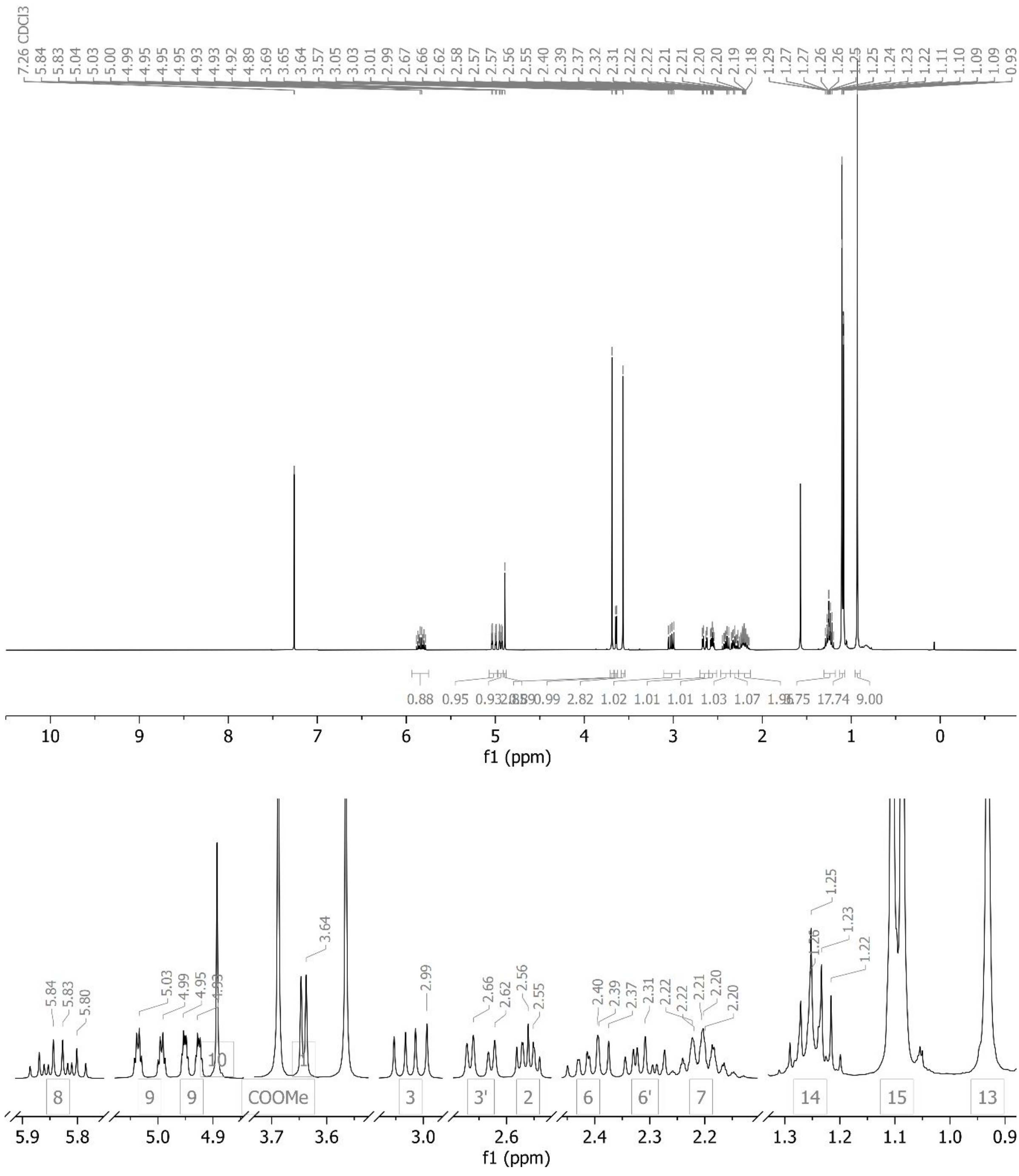

151 MHz ^{13}C NMR spectrum ($CDCl_3$)

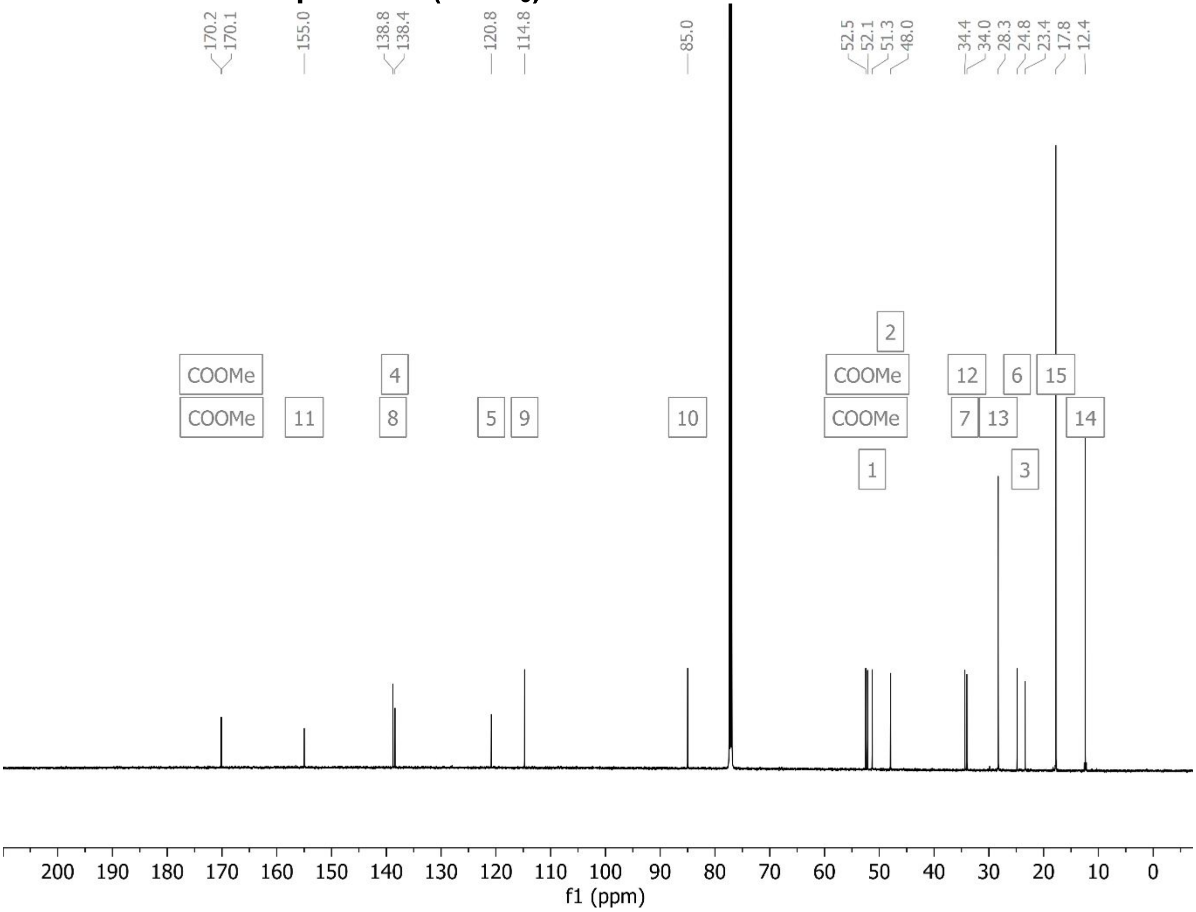

Dimethyl 2-((*R)-1-((*R**)-3-(but-3-en-1-yl)-5-oxo-2,5-dihydrofuran-2-yl)-3,3-dimethylbutan-2-yl)malonate, 23, 4:1 d.r.**

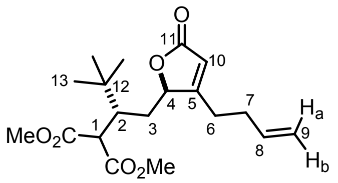

600 MHz ^{1}H NMR spectrum ($CDCl_3$)

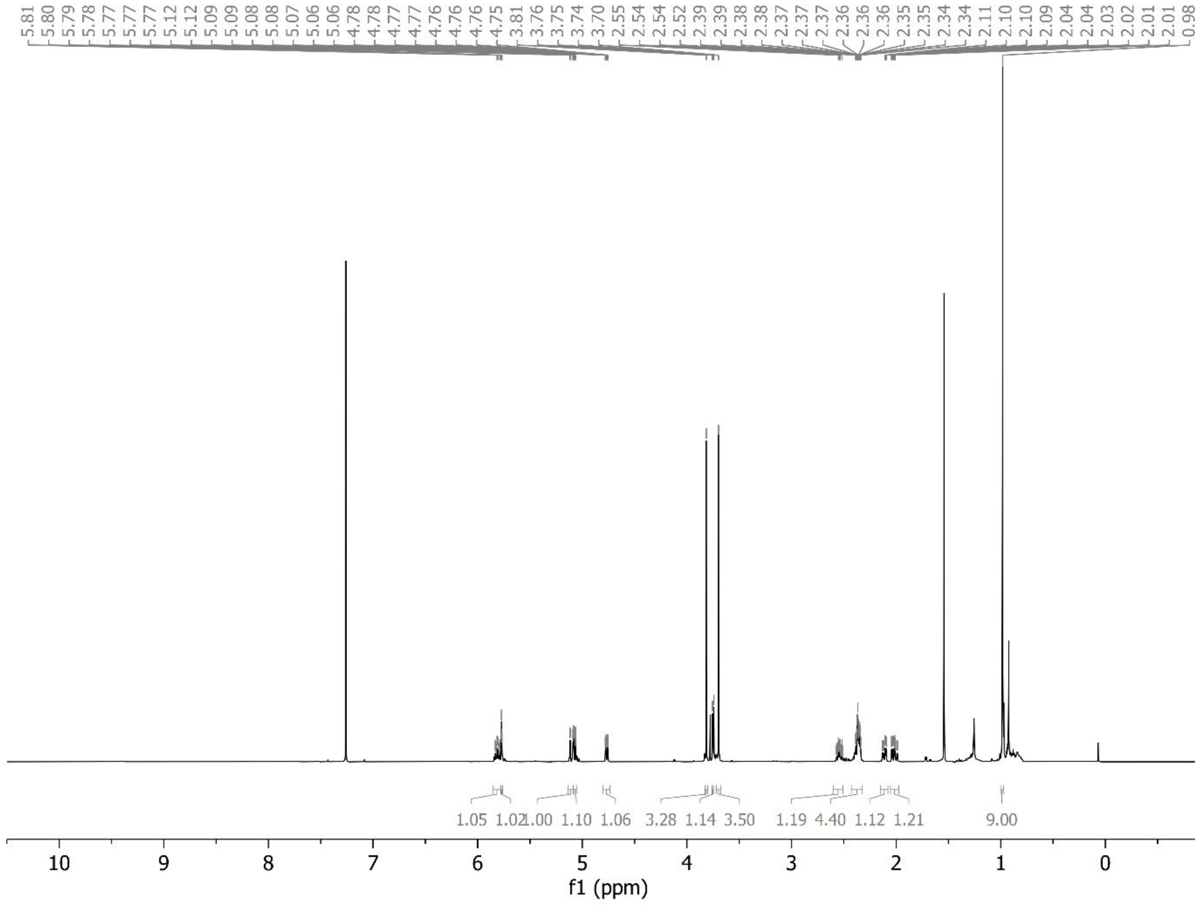

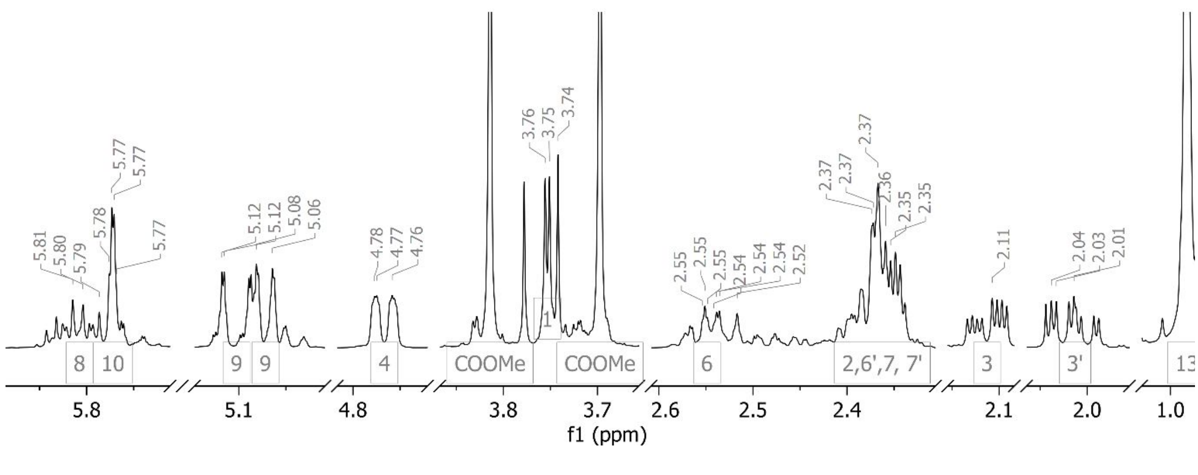

151 MHz ^{13}C NMR spectrum ($CDCl_3$)

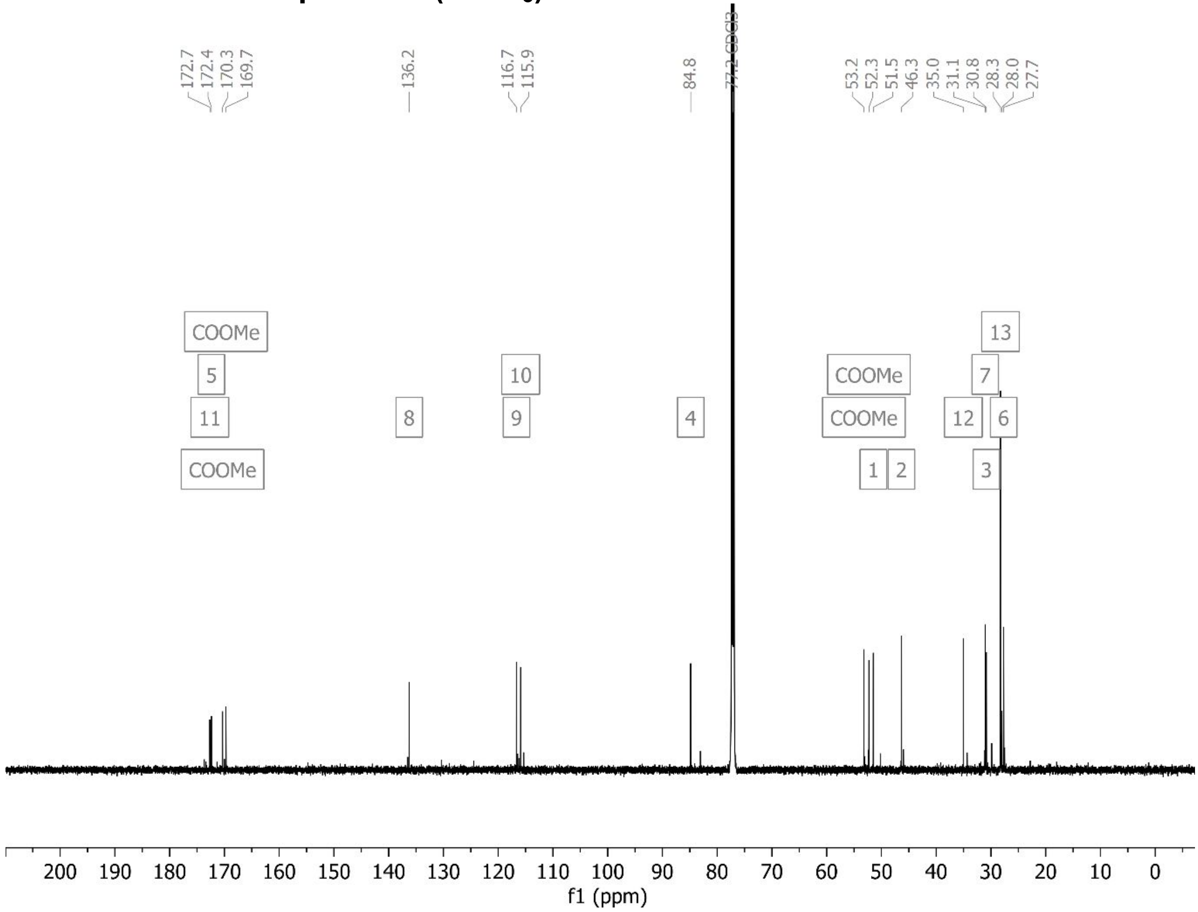

Dimethyl 2-(1-(3-(but-3-en-1-yl)-5-oxo-4,5-dihydrofuran-2-yl)-3,3-dimethylbutan-2-yl)malonate, 44

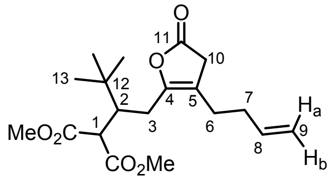

600 MHz ^{1}H NMR spectrum ($CDCl_3$)

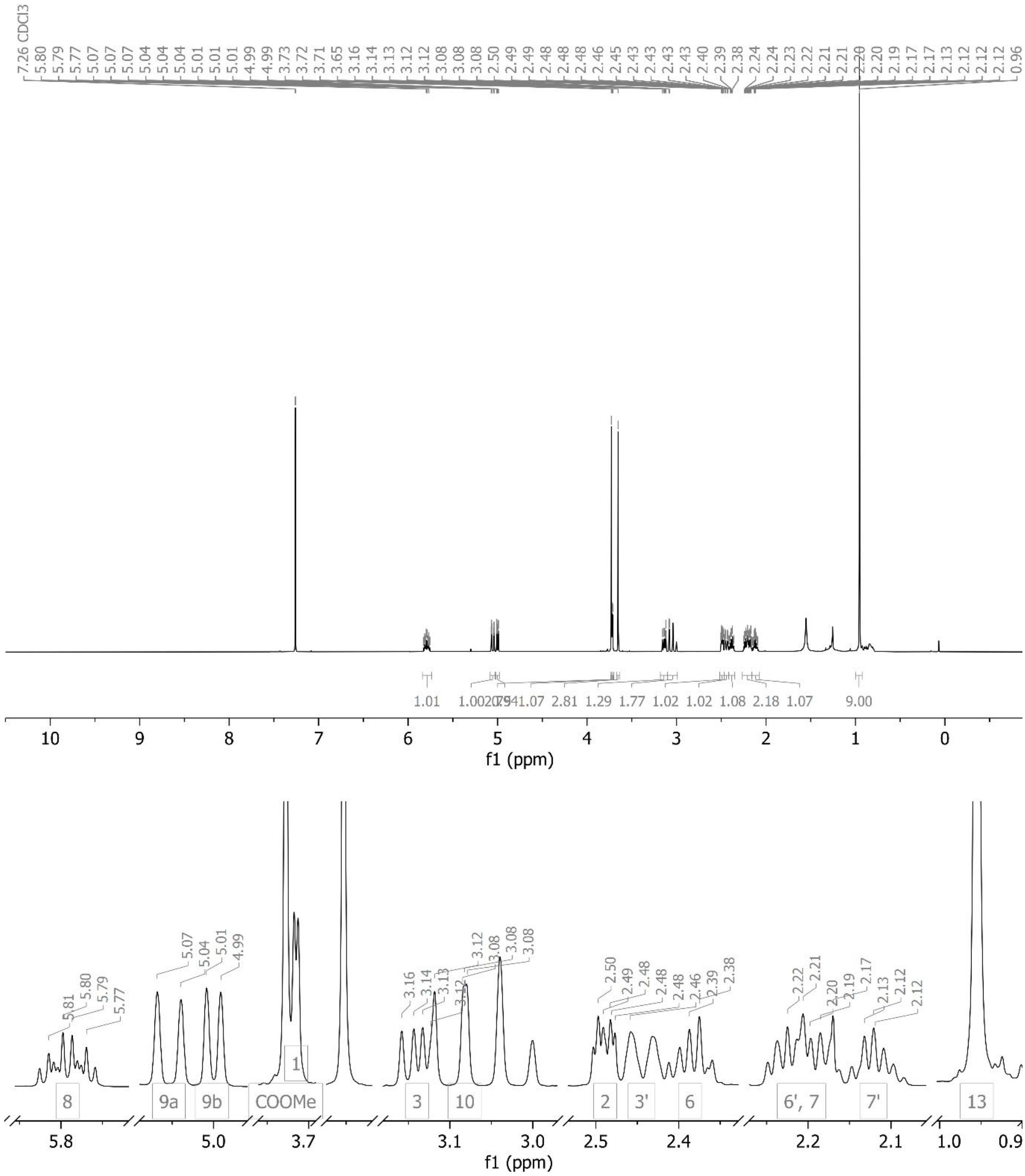

151 MHz ^{13}C NMR spectrum ($CDCl_3$)

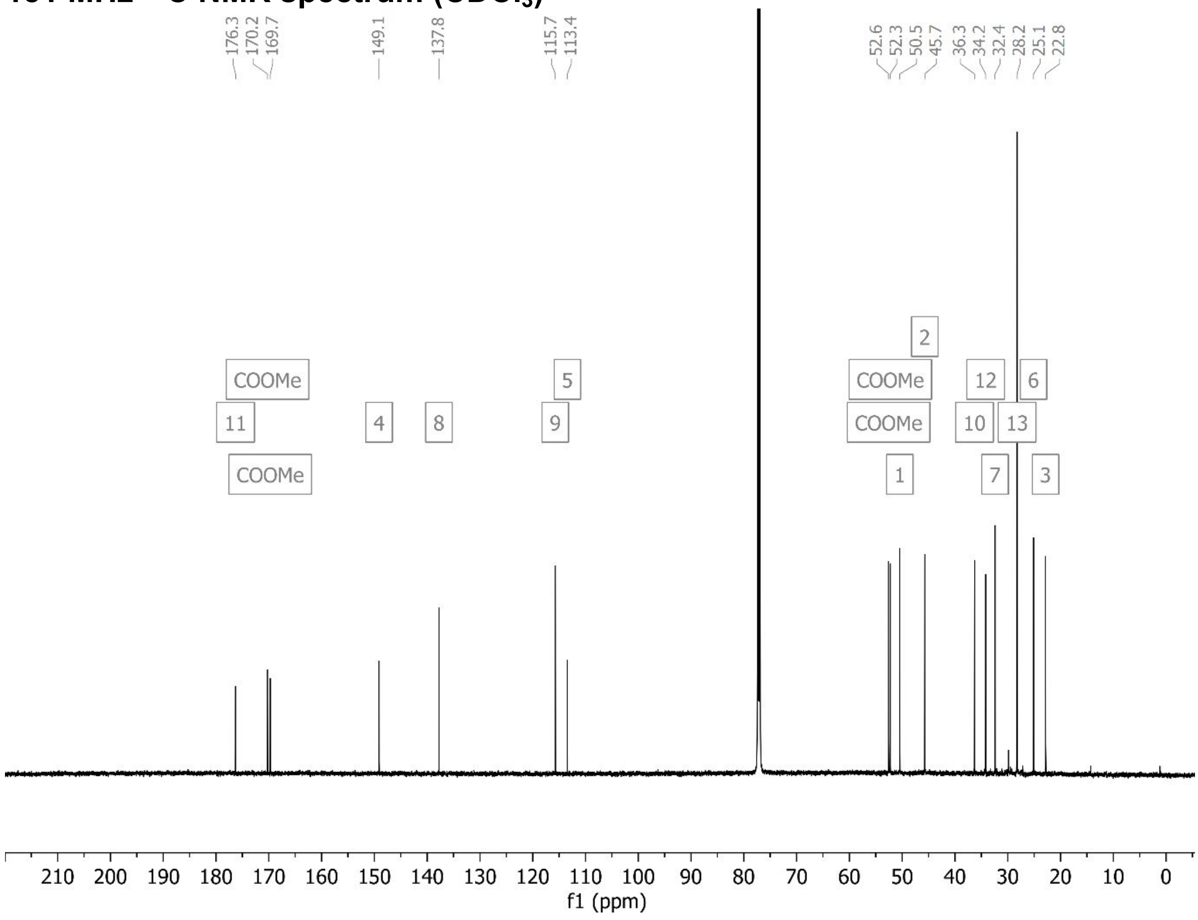

Dimethyl (2*S,3a*R**,5a*R**,8a*S**)-2-(*tert*-butyl)-6-methylene-5-oxohexahy-dro-5*H*-dicyclopenta[*b,c*]furan-1,1(2*H*)-dicarboxylate, 22**

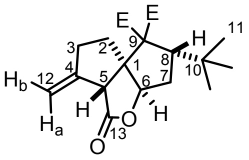

E = CO_2Me

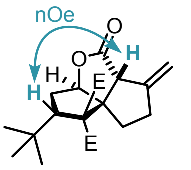

E = CO_2Me

600 MHz ^{1}H NMR spectrum ($CDCl_3$)

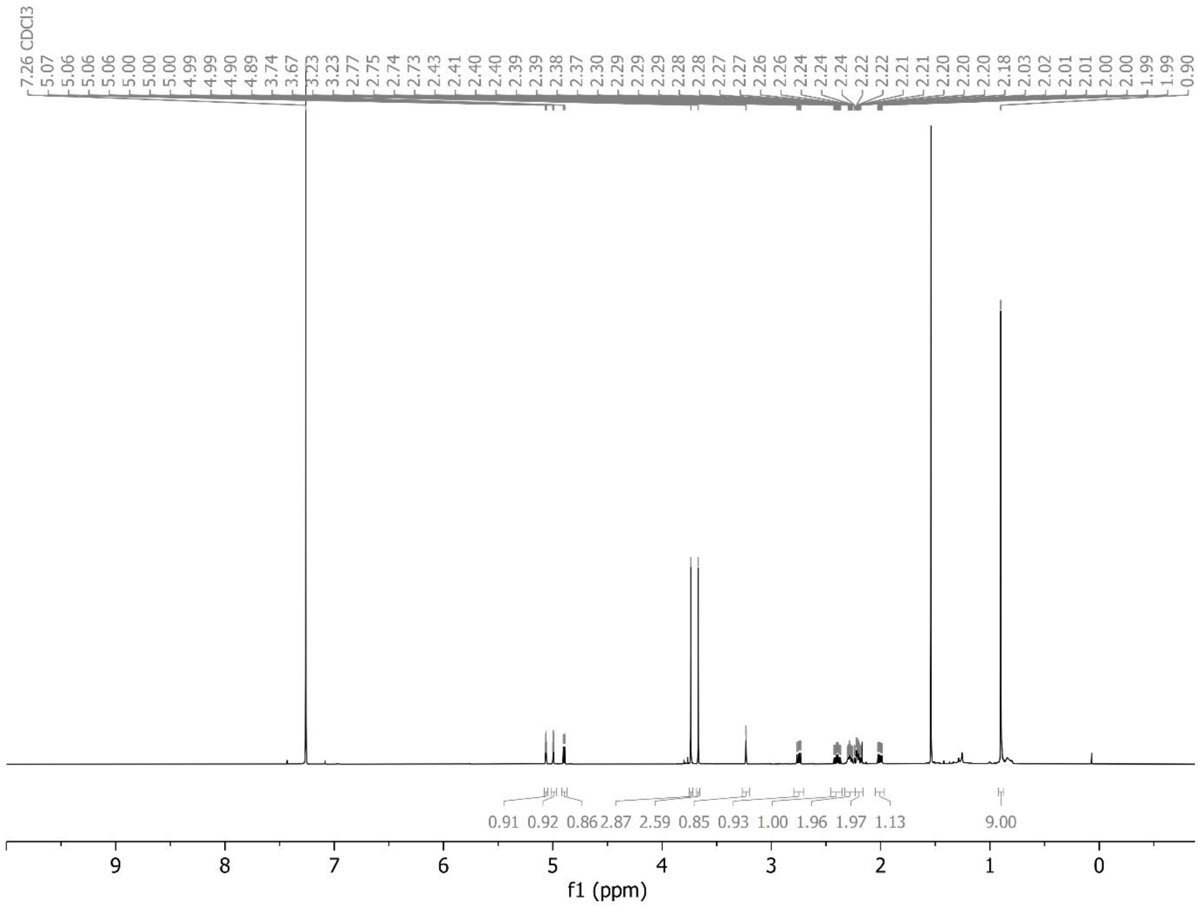

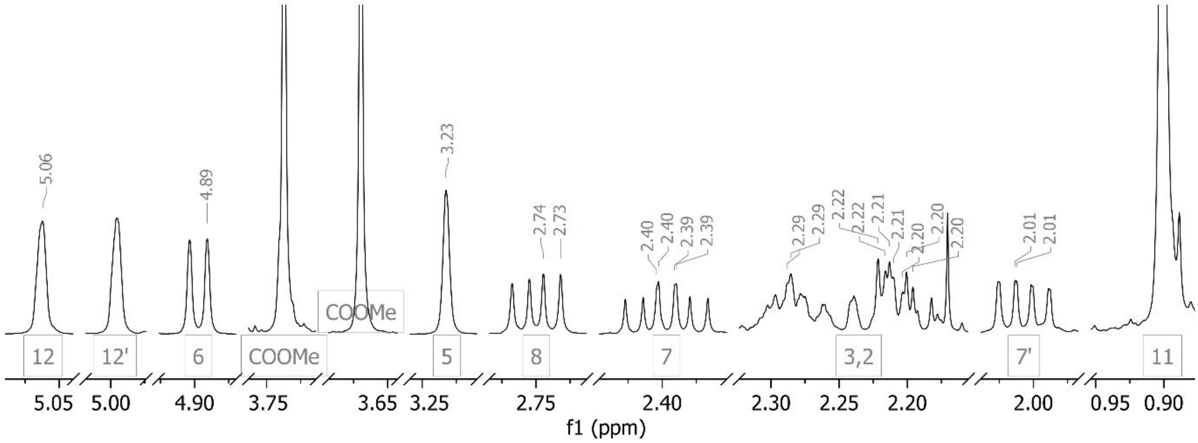

151 MHz ^{13}C NMR spectrum ($CDCl_3$)

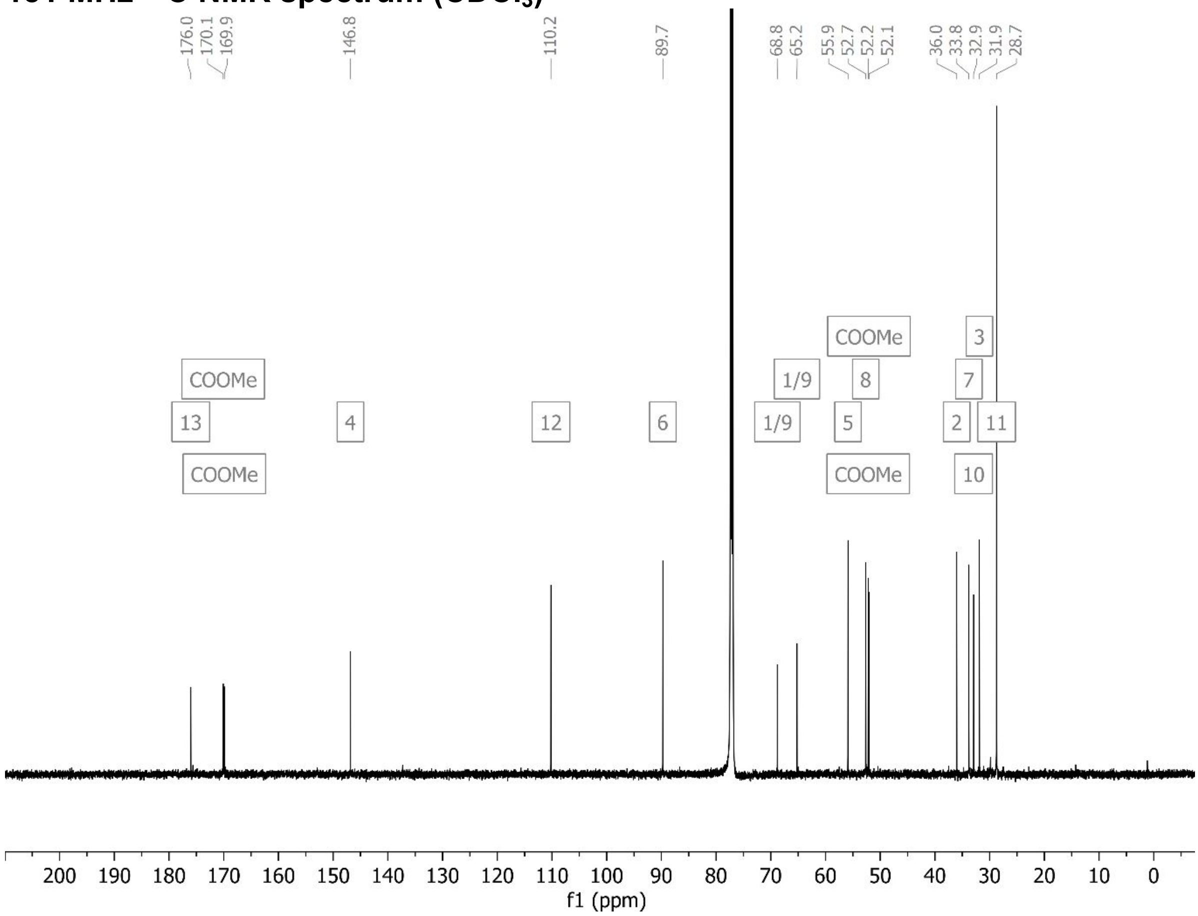

600 MHz ^1H NMR NOESY spectrum ($CDCl_3$)

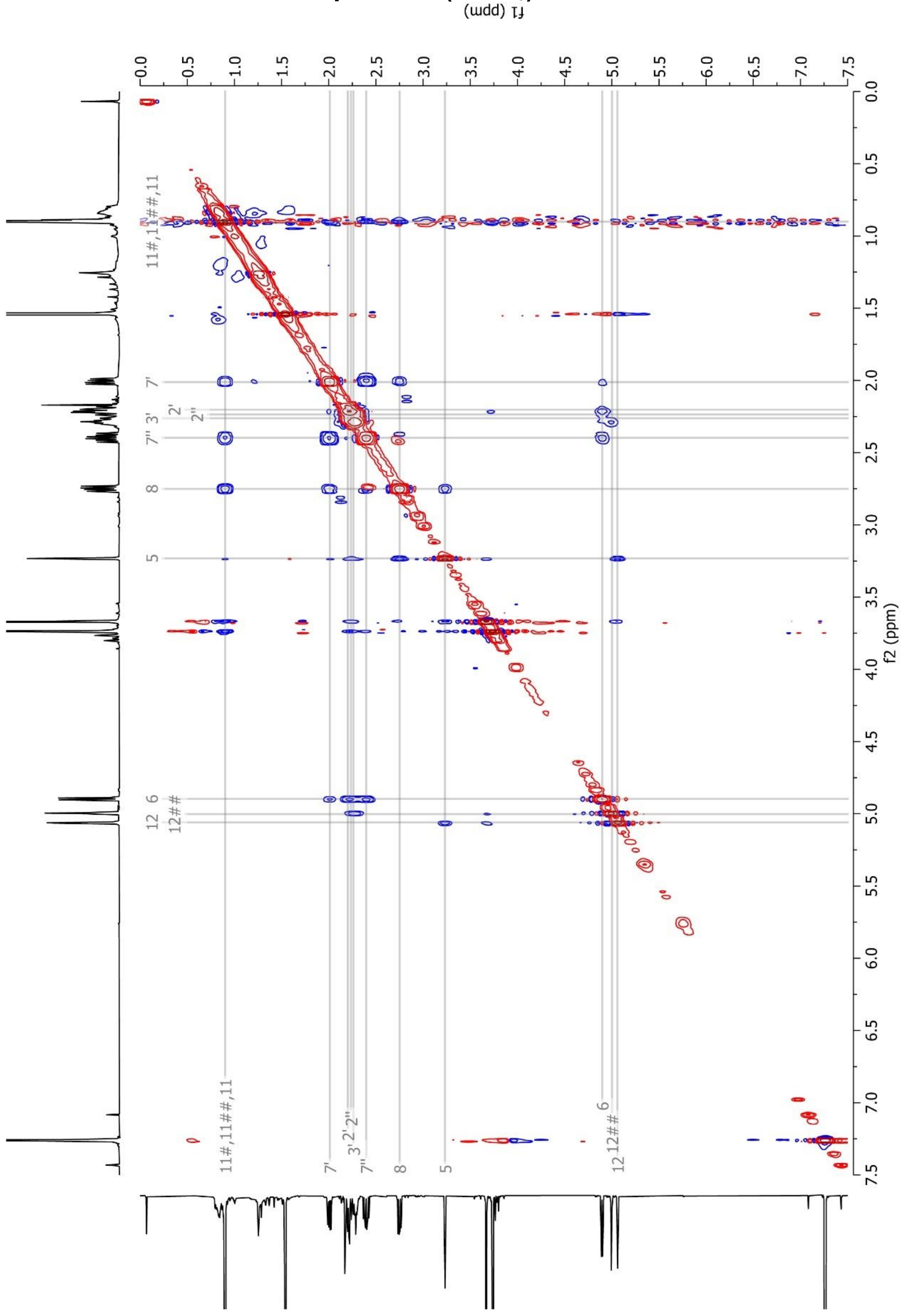

Dimethyl (2S*,3aS*,5aS*,8aR*)-2-(tert-butyl)-6-methylene-5-oxohexa-hy-dro-5H-dicyclopenta[b,c]furan-1,1(2H)-dicarboxylate, 34

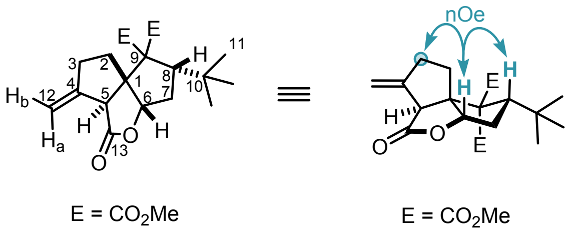

600 MHz ^1H NMR spectrum ($CDCl_3$)

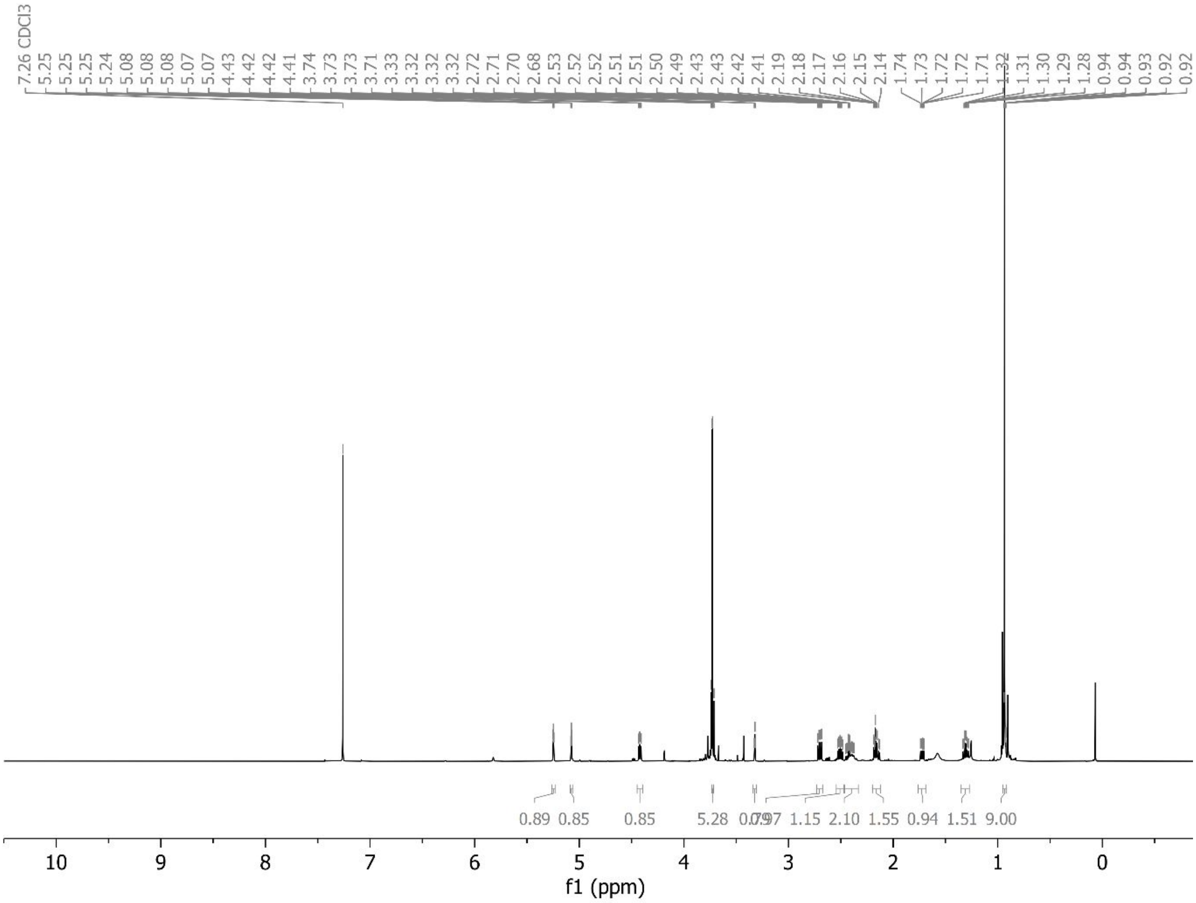

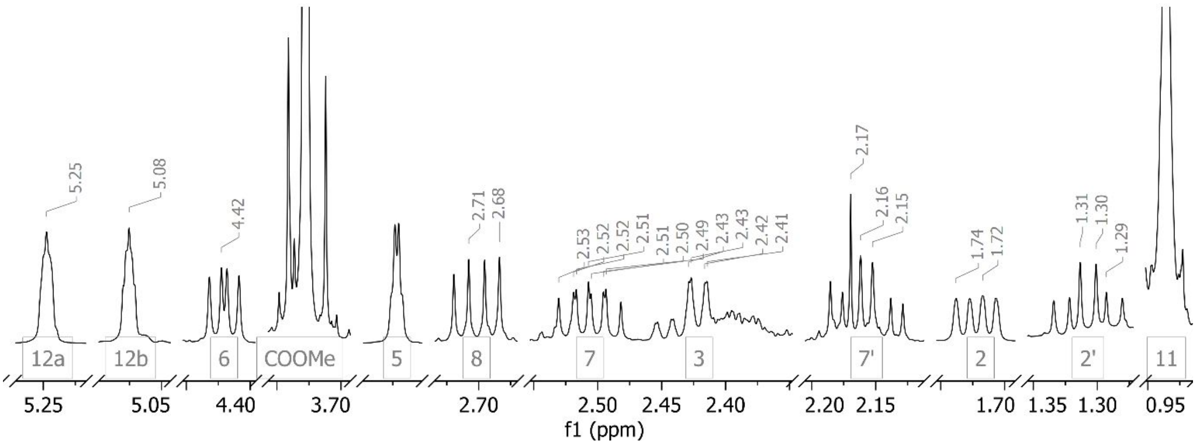

151 MHz ^{13}C NMR spectrum ($CDCl_3$)

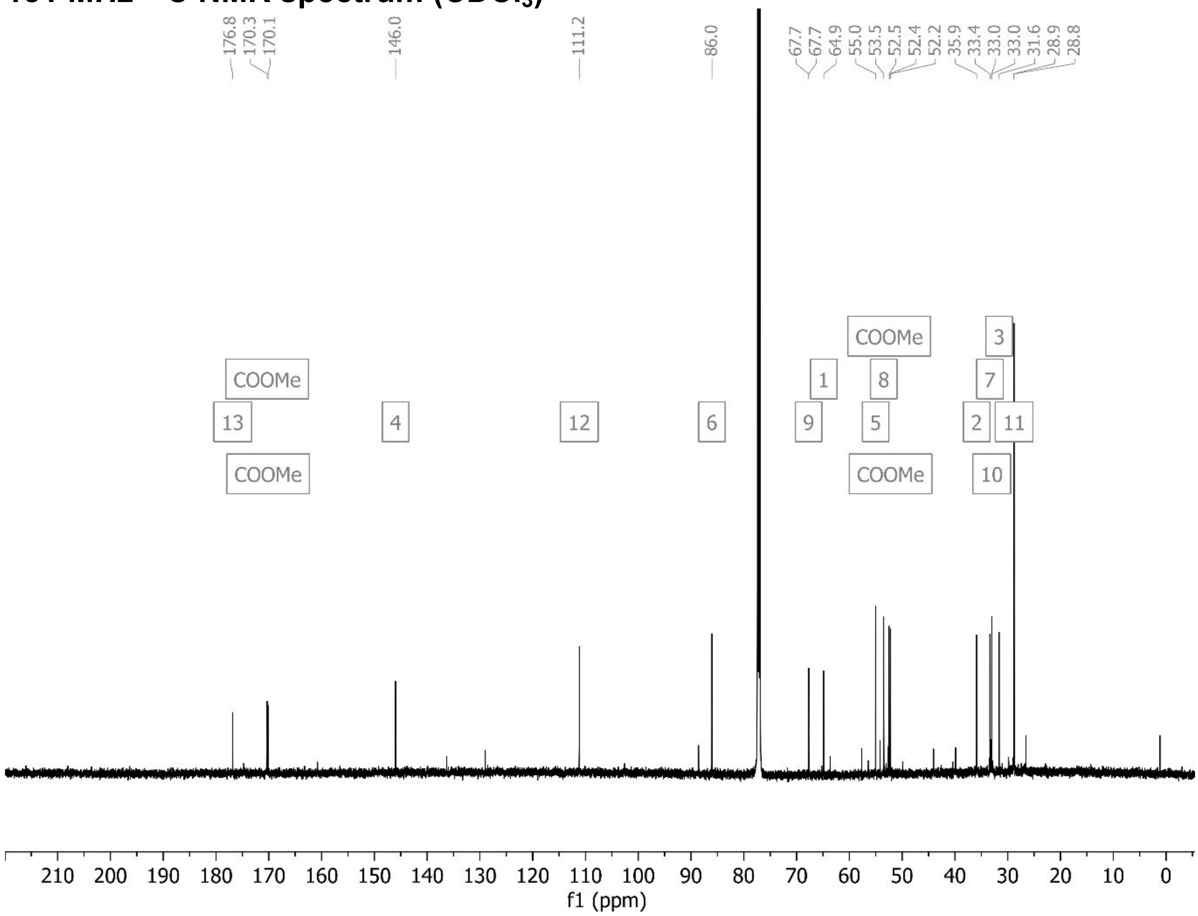

600 MHz ^{1}H NMR NOESY spectrum ($CDCl_3$)

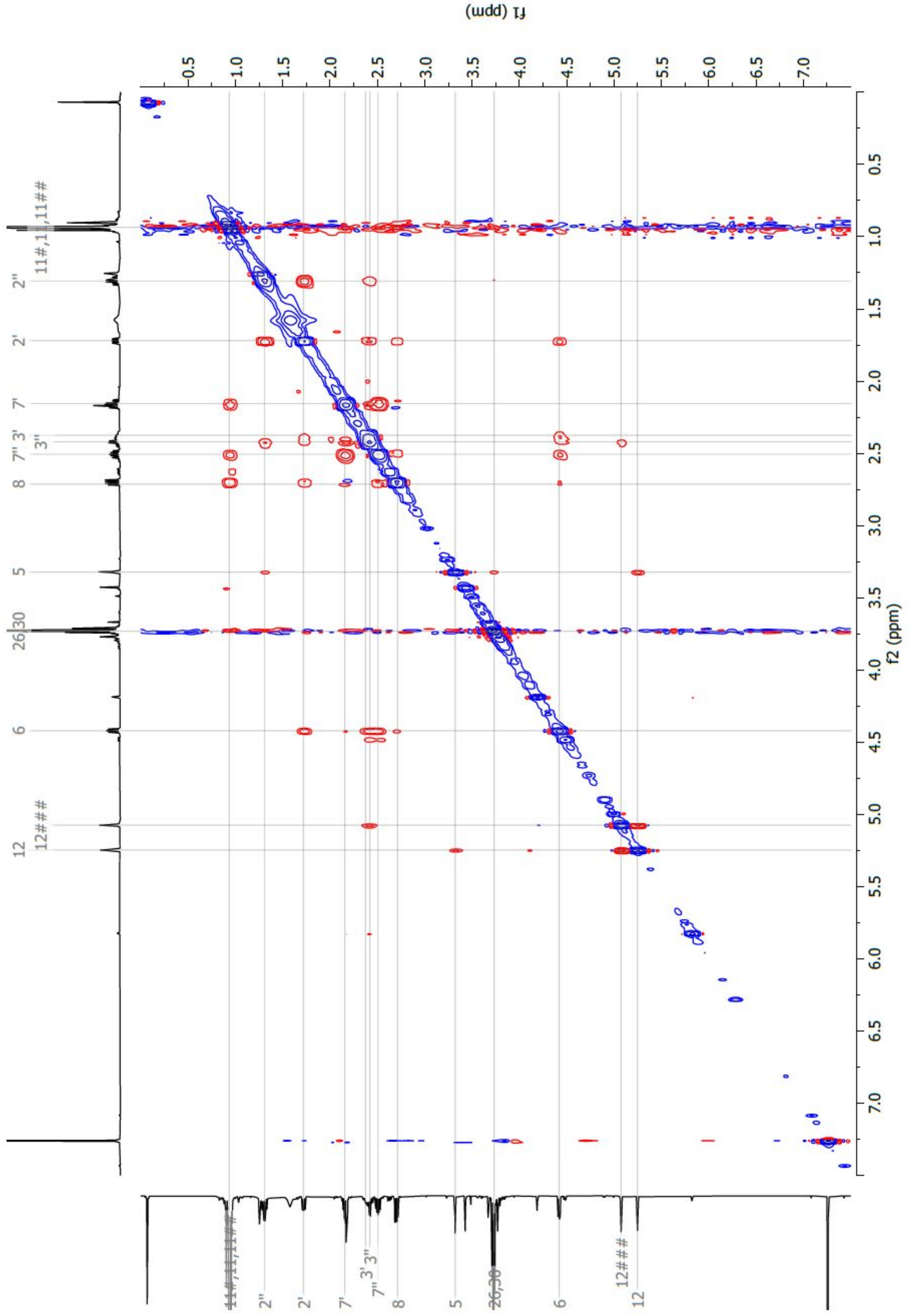

Dimethyl (2*S,3a*R**,5a*S**,8a*S**)-2-(*tert*-butyl)-5,6-dioxohexahydro-5*H*-dicyclo-penta[*b,c*]furan-1,1(2*H*)-dicarboxylate, 35**

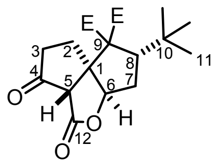

E = CO_2Me

600 MHz ^{1}H NMR spectrum ($CDCl_3$)

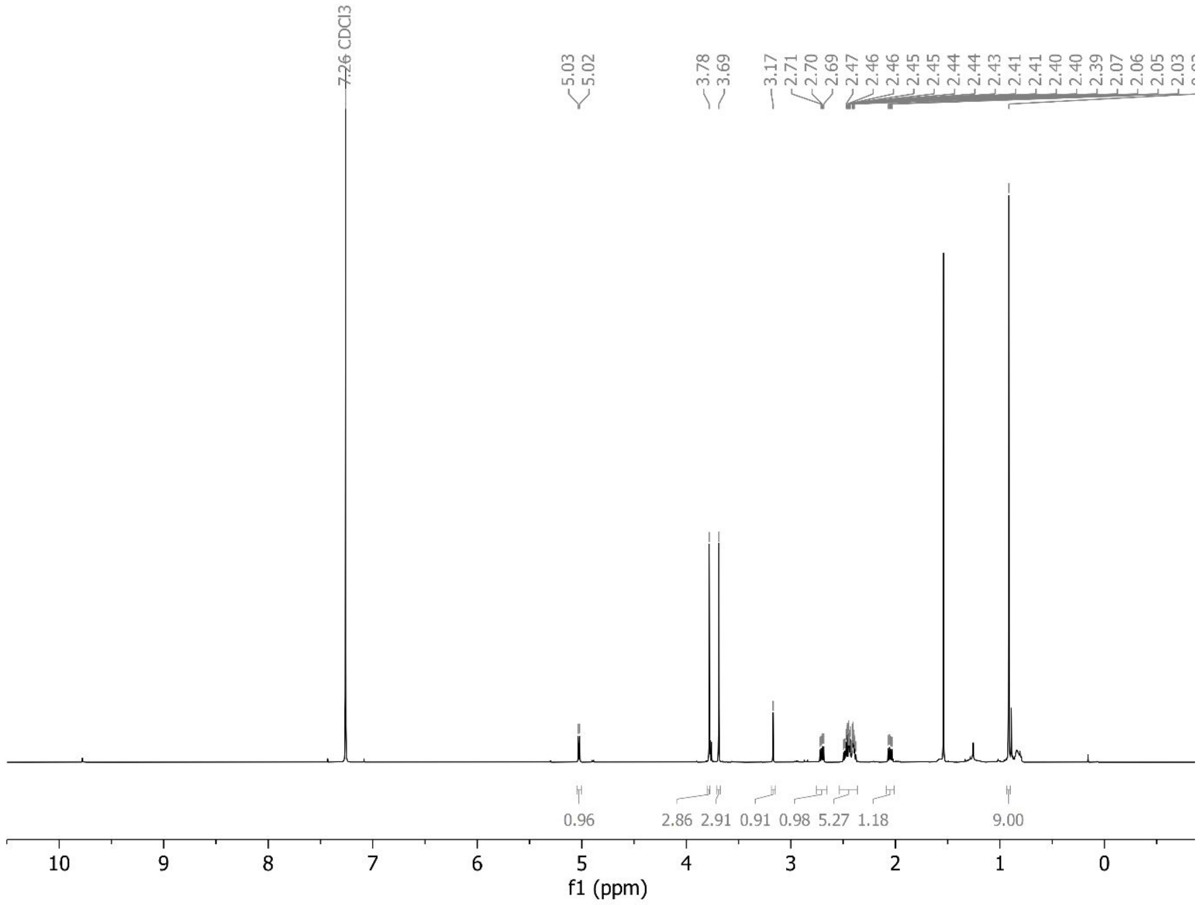

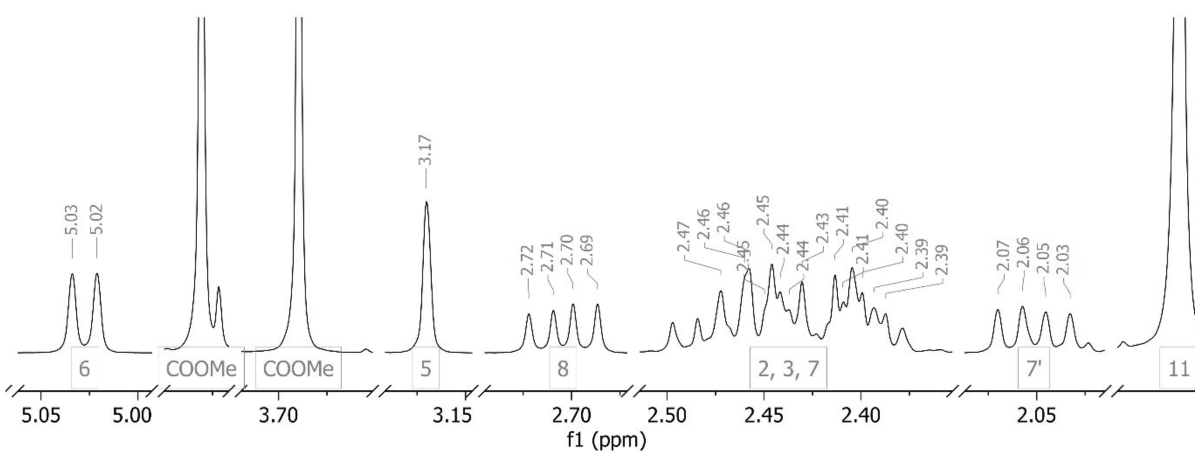

151 MHz ^{13}C NMR spectrum ($CDCl_3$)

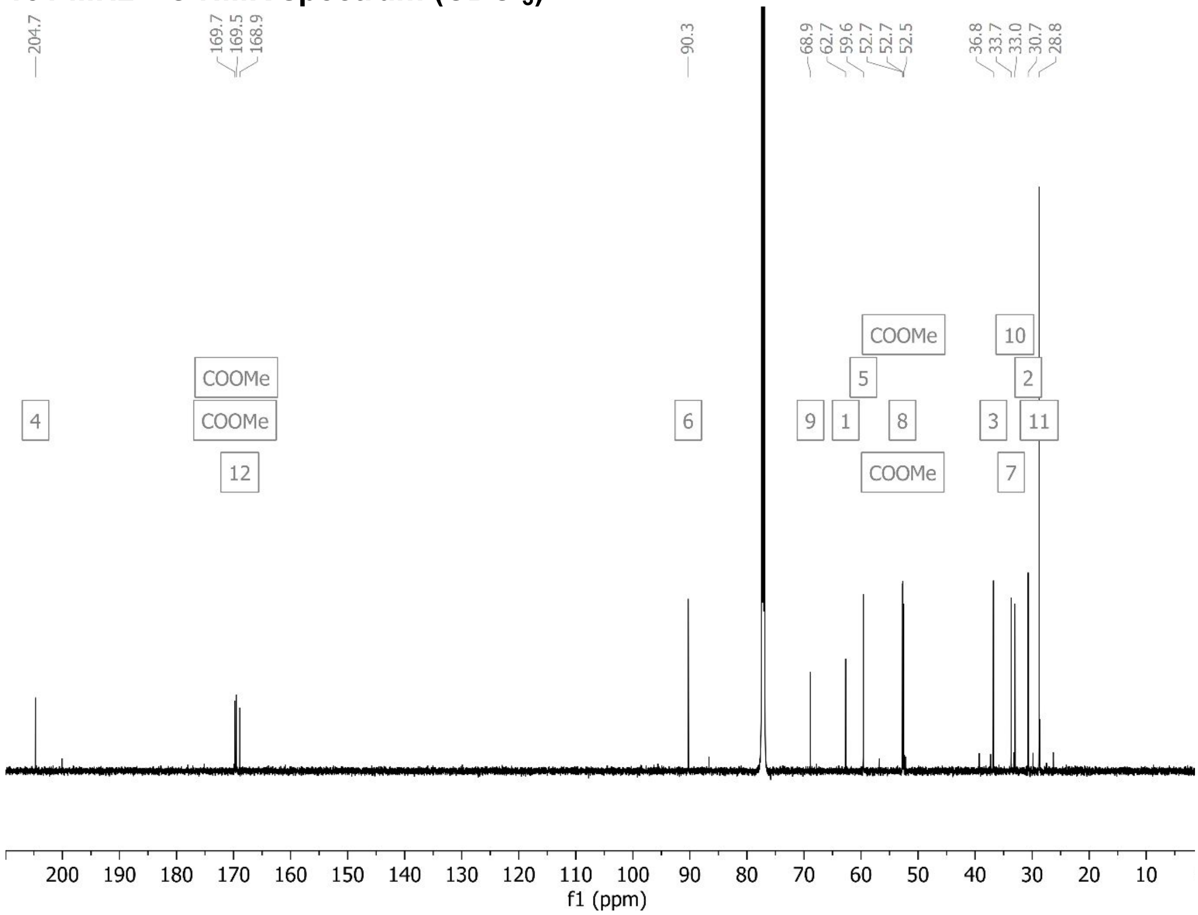

Dimethyl (2*S,3a*R**,5a*R**,9a*S**)-2-(*tert*-butyl)-5,7-dioxohexahydro-7*H*-cyclopenta[4,5]furo[3,4-*b*]pyran-1,1 (2*H*)-dicarboxylate, 39**

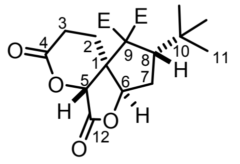

E = CO_2Me

600 MHz ^1H NMR spectrum ($CDCl_3$)

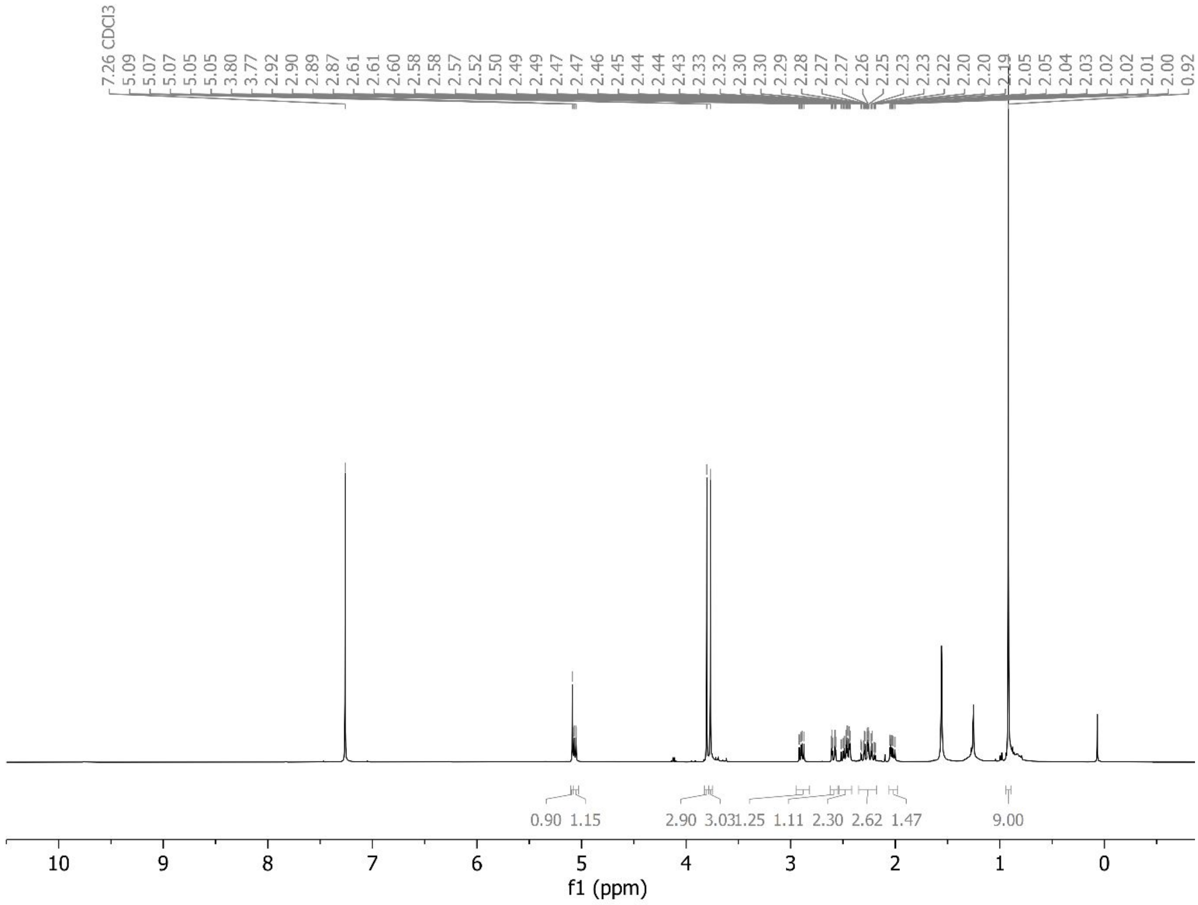

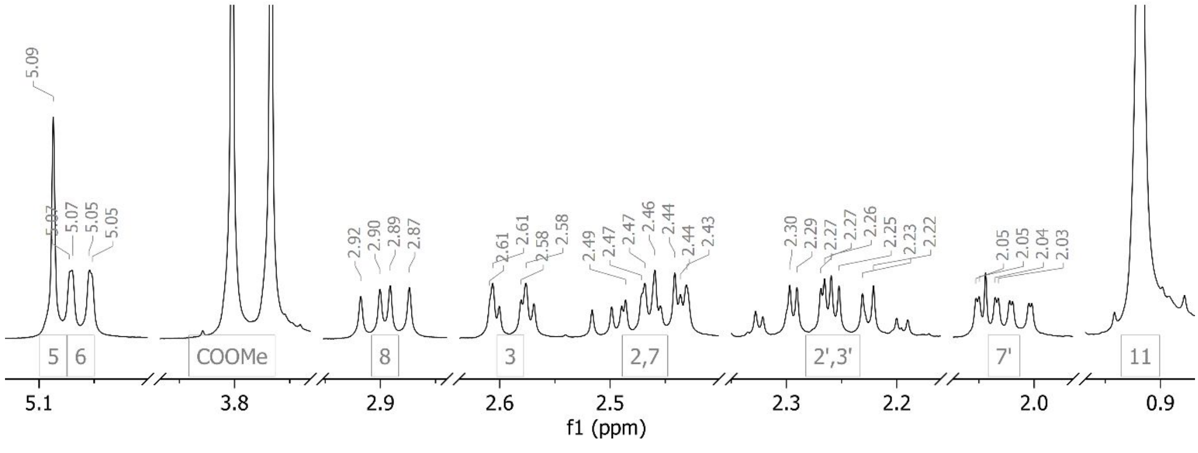

151 MHz ^{13}C NMR spectrum ($CDCl_3$)

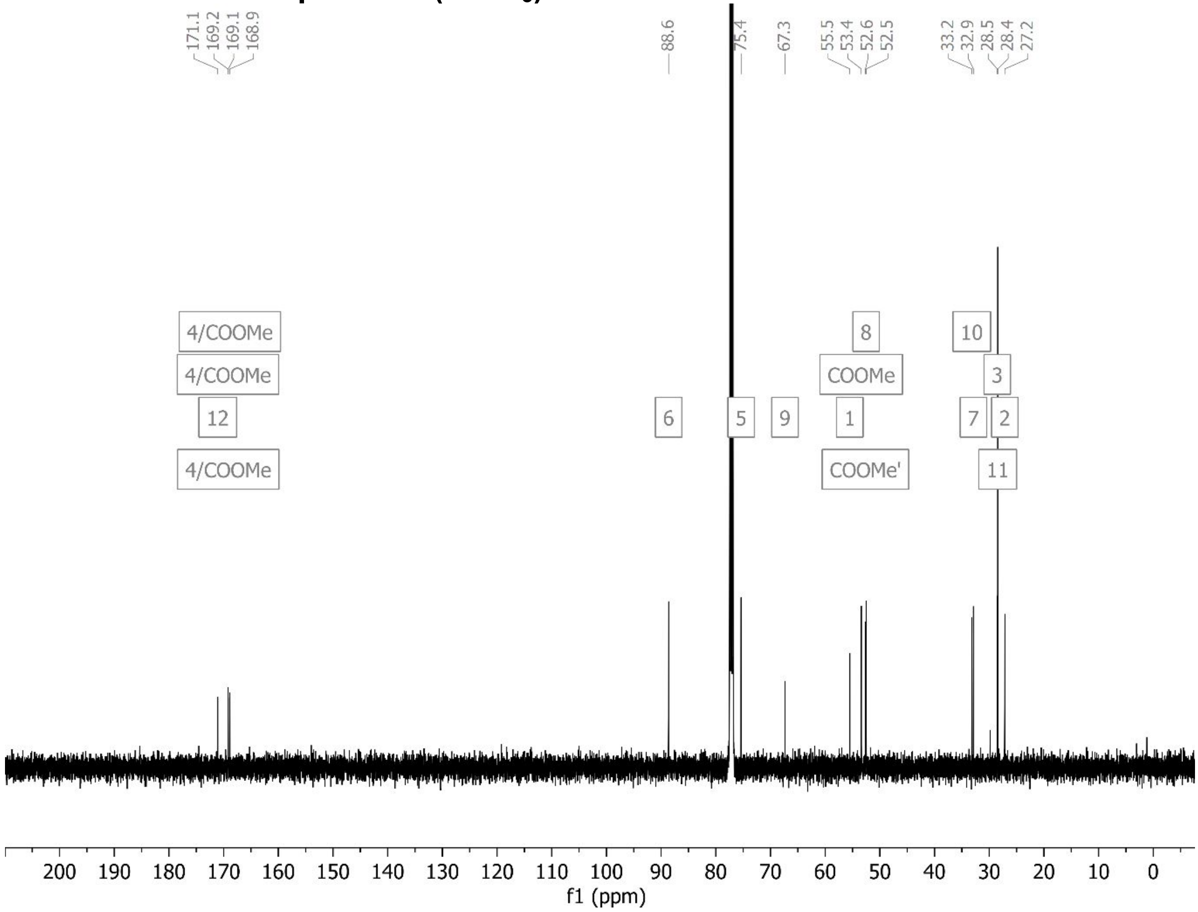

Dimethyl (2*S,3a*R**,5a*R**,8a*S**)-5a-(((1*S*,5*S*)-9-azabicyclo[3.3.1]nonan-9-yl)oxy)-2-(*tert*-butyl)-5,6-dioxohexahydro-5*H*-dicyclopenta[*b,c*]furan-1,1-(2*H*)-dicarboxylate, 40**

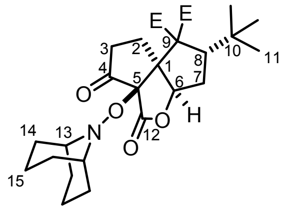

E = CO_2Me

600 MHz ^{1}H NMR spectrum ($CDCl_3$)

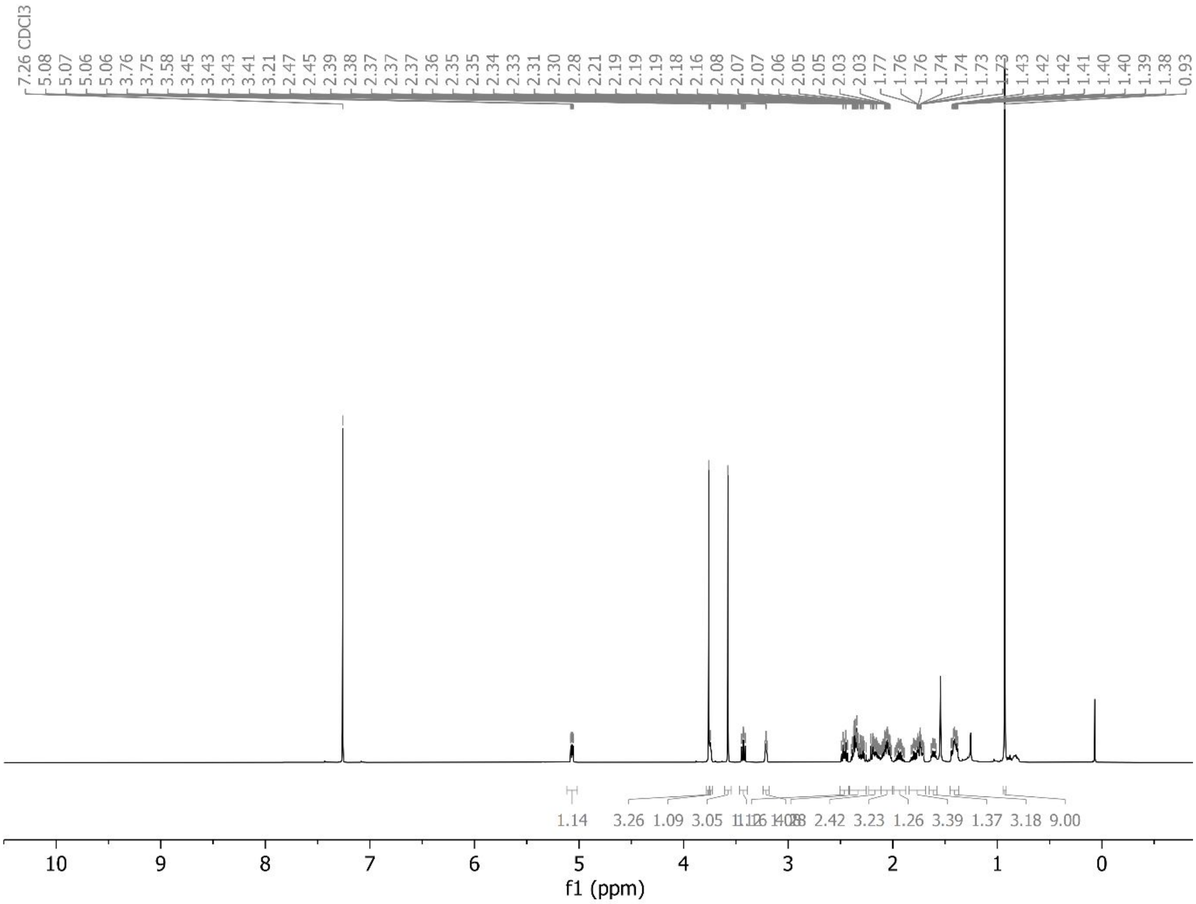

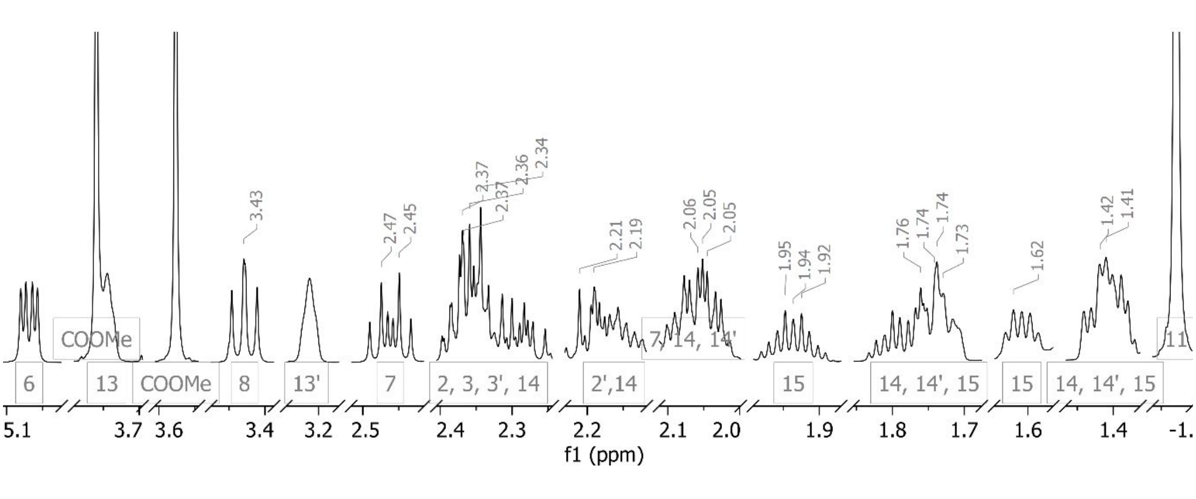

151 MHz ^{13}C NMR spectrum ($CDCl_3$)

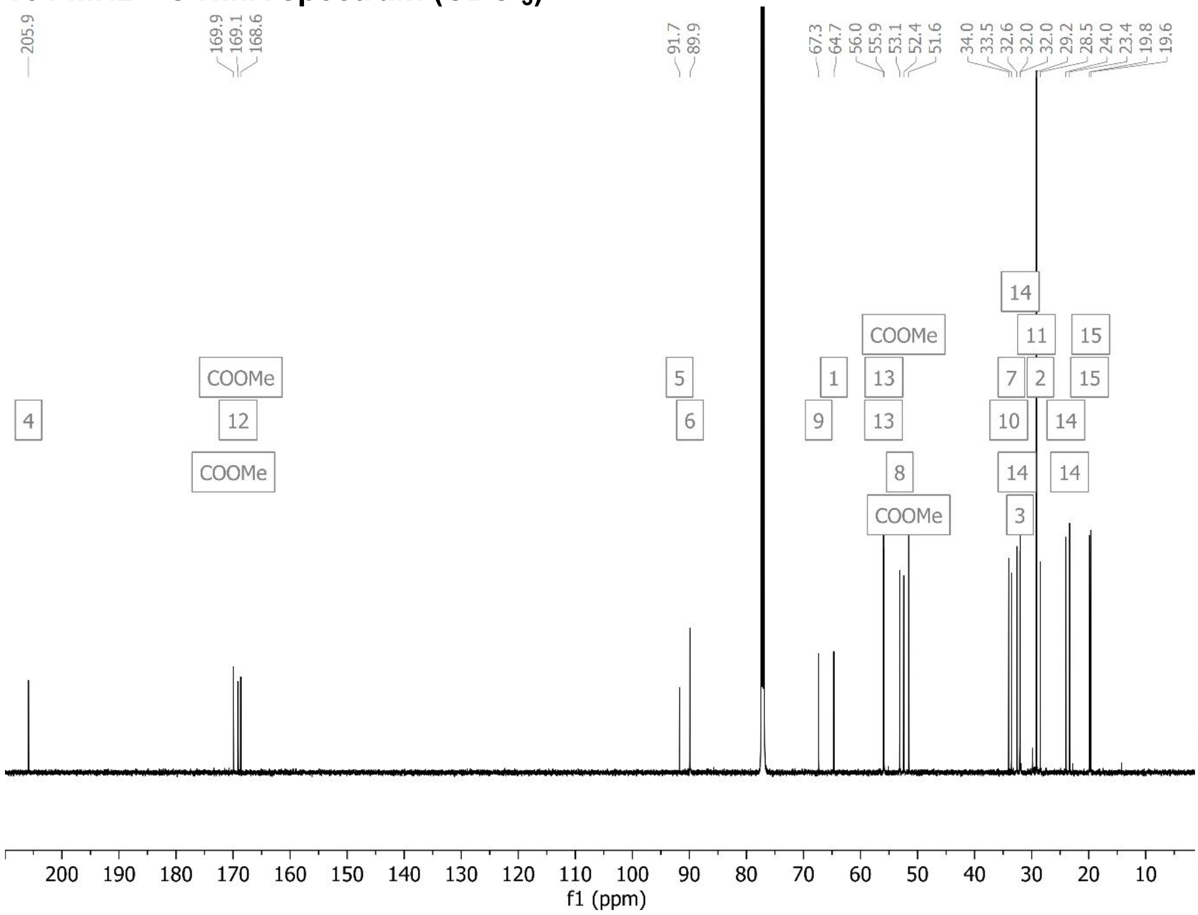

Dimethyl (2*S,3a*R**,5a*S**,8a*S**)-2-(*tert*-butyl)-7-hydroxy-5,6-dioxo-3,3a,5a,6-tetrahydro-5*H*-dicyclopenta[*b*,*c*]furan-1,1(2*H*)-dicarboxylate, 36**

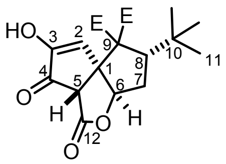

E = CO_2Me

600 MHz ^{1}H NMR spectrum ($CDCl_3$)

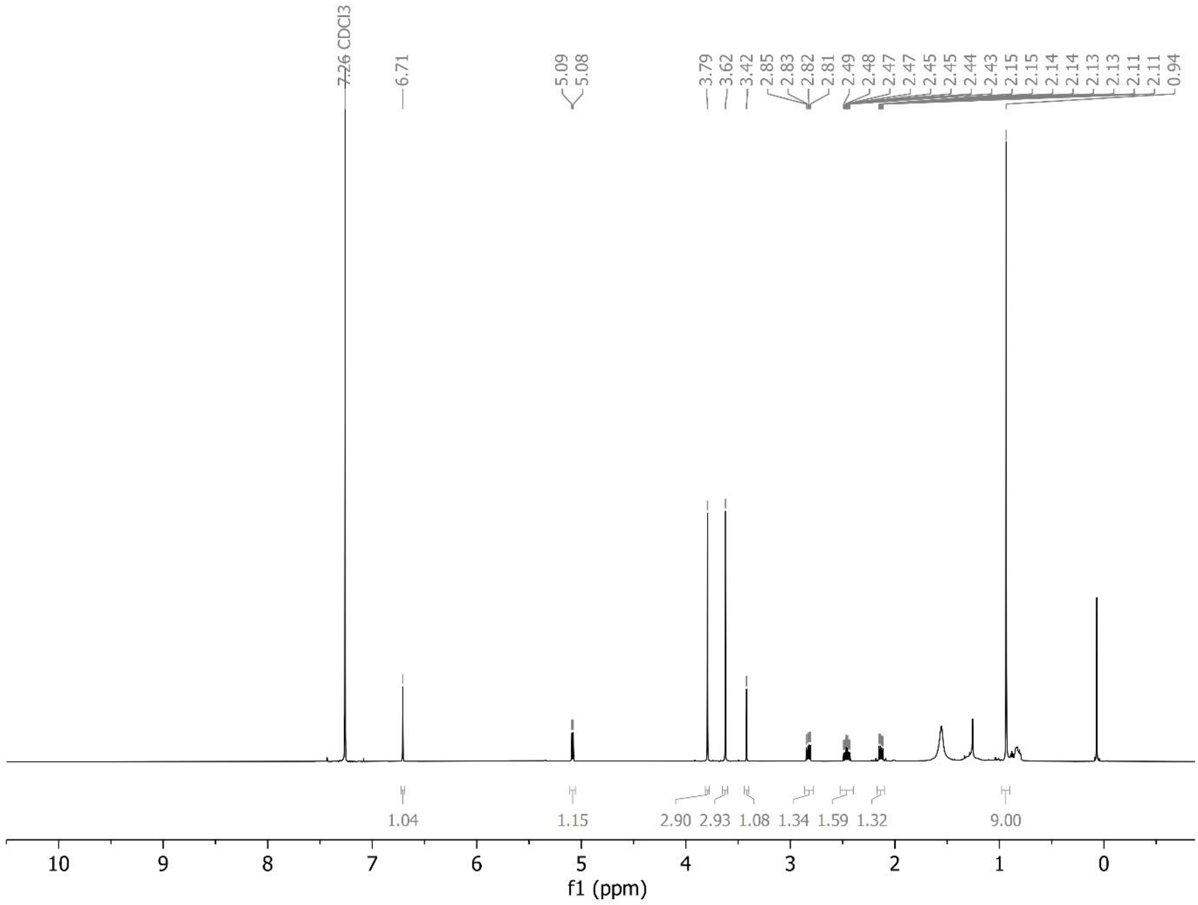

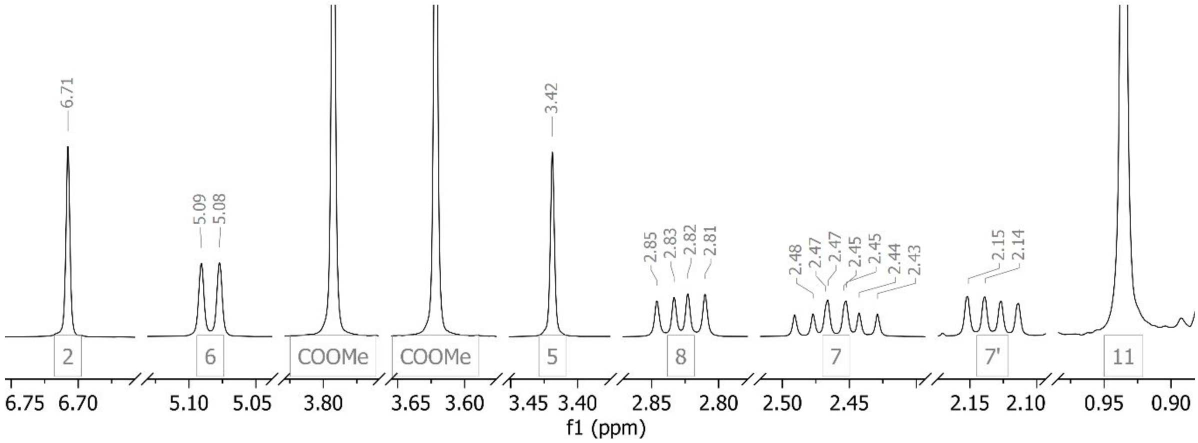

151 MHz ^{13}C NMR spectrum ($CDCl_3$)

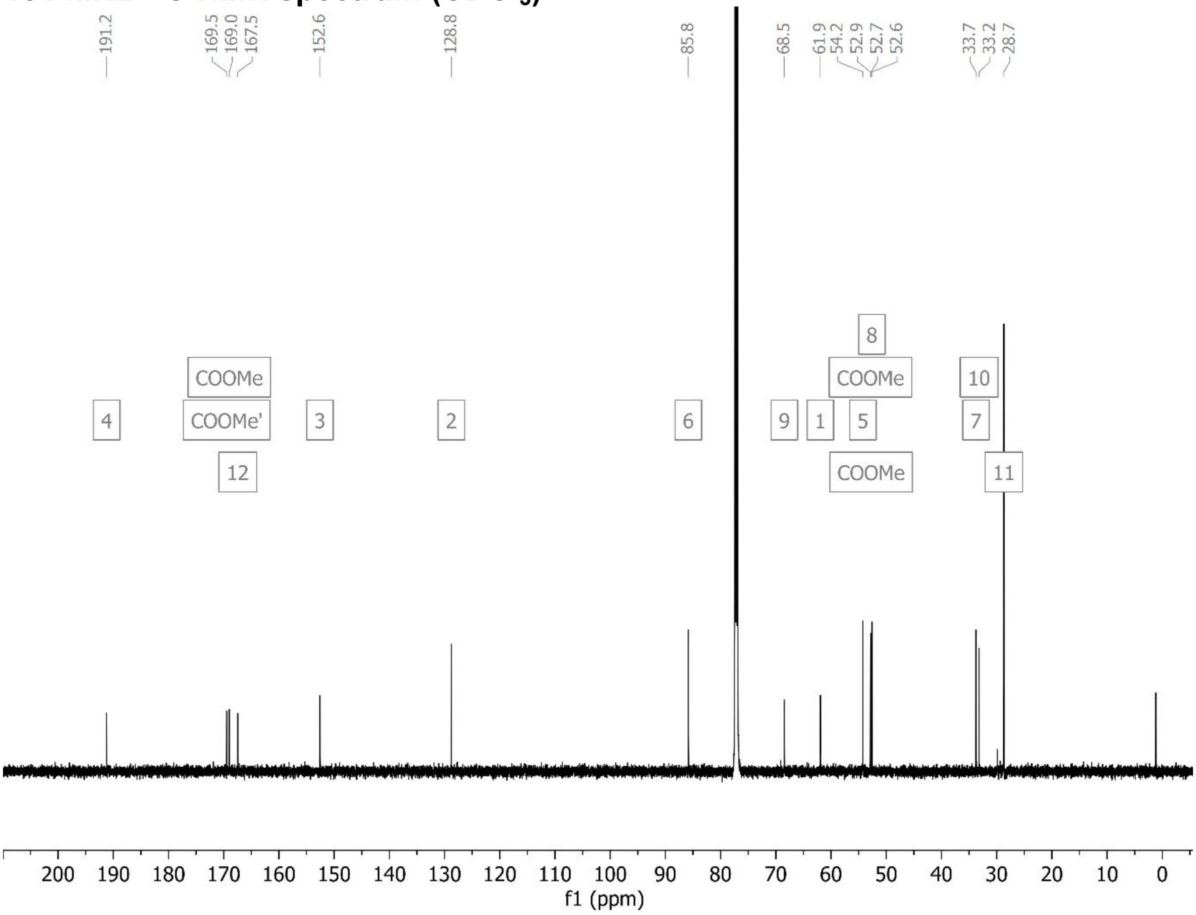

Methyl (1*S,2*S**,3a*R**,5a*R**,8a*S**)-2-(*tert*-butyl)-5,6,10-trioxotetrahydro-5*H*,6*H*-5a,1-(epoxymethano)dicyclopenta[*b,c*]furan-1(2*H*)-carboxylate, 9**

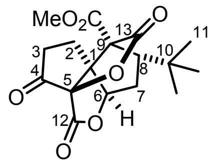

600 MHz ^{1}H NMR spectrum ($CDCl_3$)

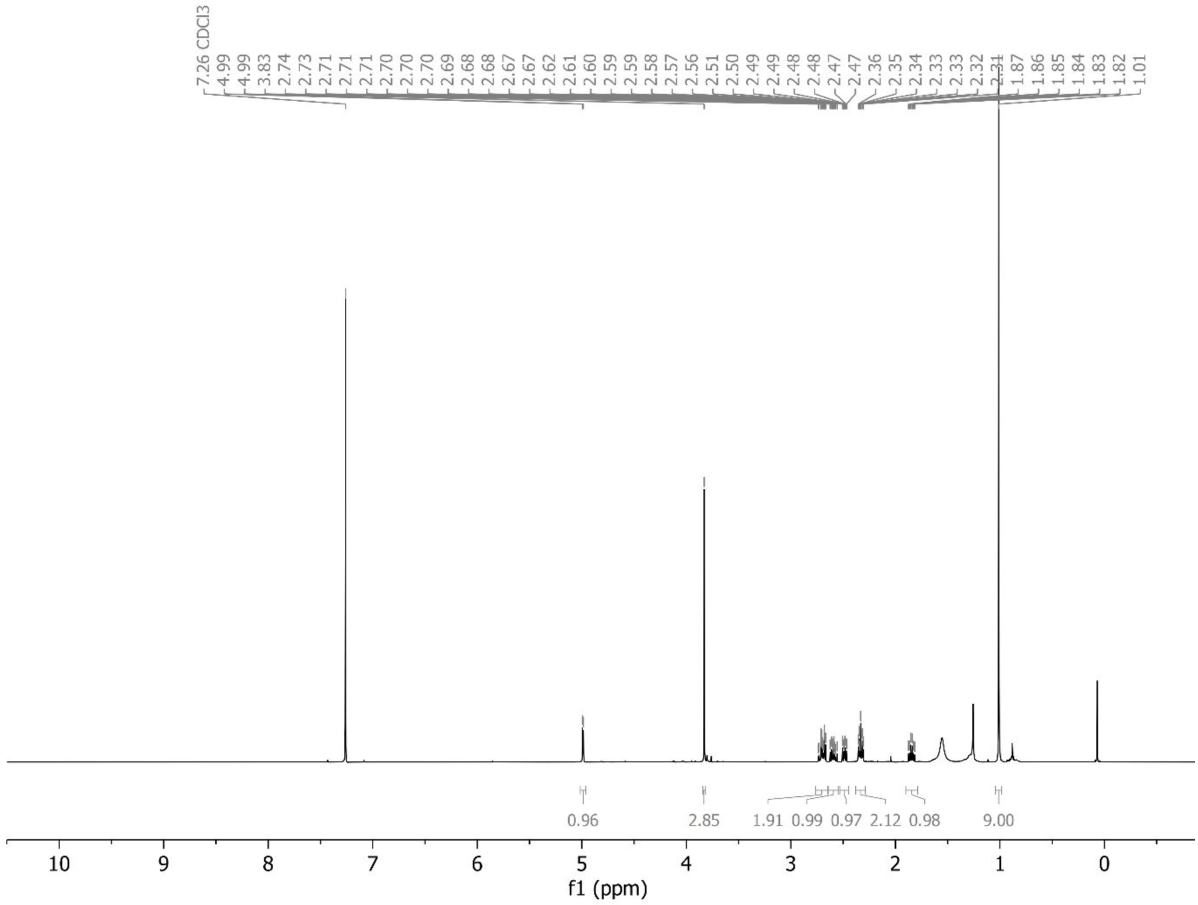

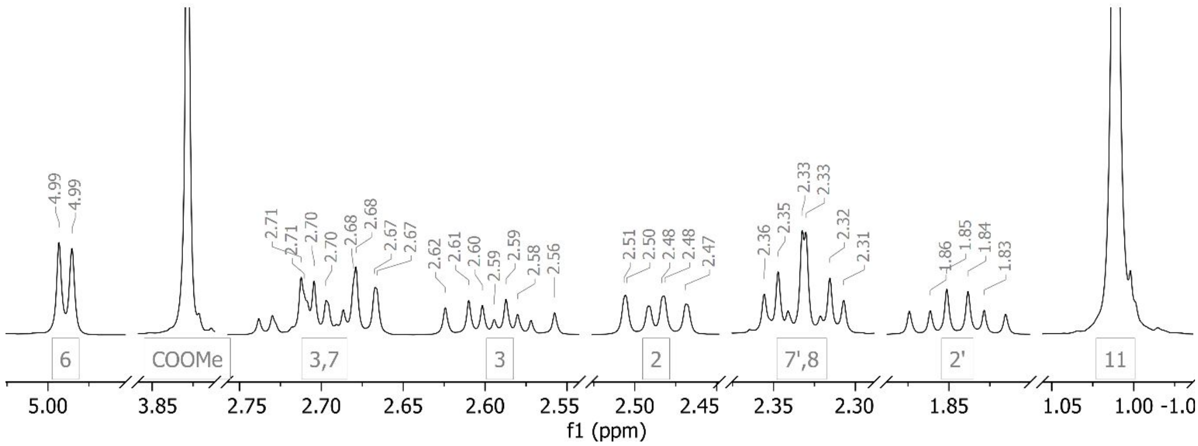

151 MHz ^{13}C NMR spectrum ($CDCl_3$)

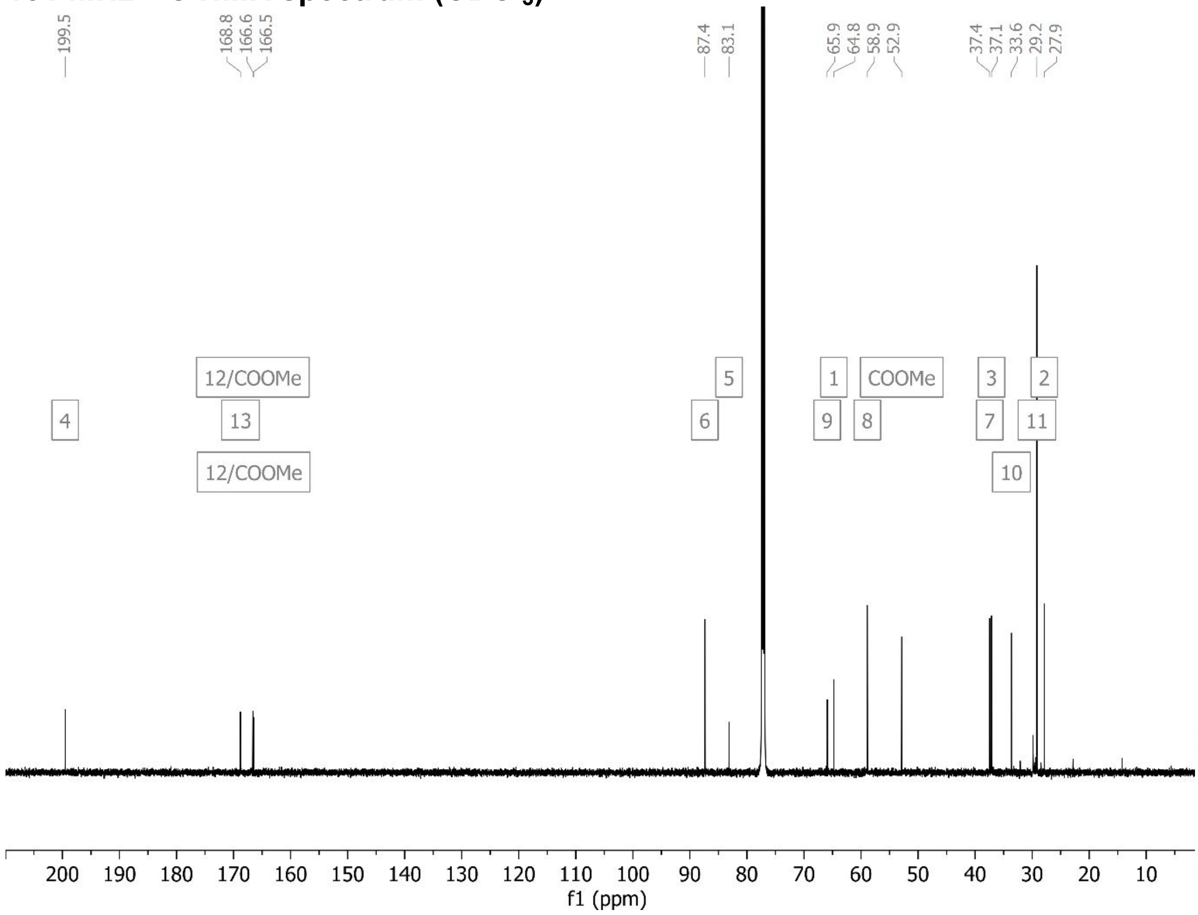

4. Studies on the epimerisation of γ-butenolides

A short screen showed that no isomerisation of γ-butenolide **23'** occurred under acidic or basic conditions (**Table S1**).

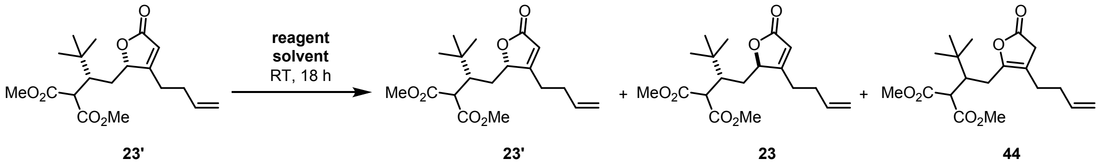

Entry	Reagent (equiv.)	Solvent	Outcome
1	DBU (0.25)	$CDCl_3$	No change
2	NEt_3 (0.25)	$CDCl_3$	No change
3	TFA (0.25)	$CDCl_3$	No change
4	tBuOK (0.25)	tBuOH	No change

Table S1. Results of the epimerisation screen of γ-butenolide **23'**. *Reagents and conditions:* **23'** (0.05 mmol, 4:1 d.r.), reagent, solvent, RT, 18 h.

These results prompted us to investigate a silylation-desilylation approach. A summary of the results obtained upon desilylation of **43** are shown below.

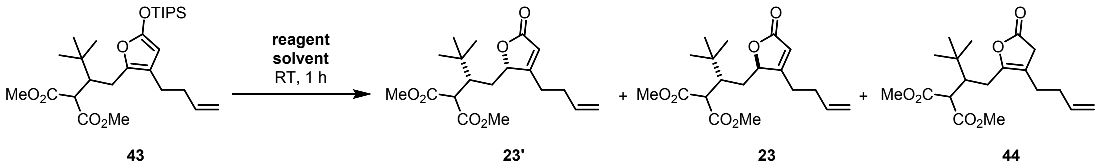

Entry	Reagent (equiv.)	Solvent	Yield of 23+23'/%[a]	d.r. (23:23')[b]	Yield of 44/%[a]
1	TFA (50)	CH_2Cl_2	54[c]	1:6	32[c]
2[d]	TBAF (5), AcOH (10)	CH_2Cl_2	–	–	11[c,e]
3	2 M aq. HCl (50)	THF	41	3:1	29
4	2 M aq. HCl (50)	MeOH	8	3:1	30
5	2 M aq. HCl (50)	tBuOH	20	3:1	33
6	AcOH	–	57	3:1	6
7[f]	AcOH	–	87[c]	3:1	<5

Table S2 Results of the desilylation of silyloxyfuran **43**. *Reagents and conditions:* **43** (0.05 mmol), reagent, solvent, RT, 1 h. [a] Calculated yield by ^1H NMR using 1,3,5-trimethoxybenzene as internal standard; [b] Diastereoisomeric ratio measured by ^1H NMR of the reaction crude; [c] Isolated yield; [d] Reaction performed at 0 °C for 10 min; [e] Extensive decomposition observed by ^1H NMR; [f] Reaction performed on 7.54 mmol of furan **43**.

5. X-ray crystallographic data

Single crystals of compound **35** were obtained by interlayer diffusion of hexanes into EtOAc solution at room temperature over 2 weeks.

Low temperature (J. Appl. Crystallogr., 1986, 19, 105) single crystal X-ray diffraction data were collected using a (Rigaku) Oxford Diffraction SuperNova diffractometer. Raw frame data were reduced using CrysAlisPro and the structures were solved using 'Superflip' [Palatinus, L.; Chapuis, G. J. Appl. Crystallogr. 2007, 40, 786-790] before refinement with CRYSTALS [(J. Appl. Crystallogr., 2003, 36, 1487; Parois, P.; Cooper, R. I.; Thompson, A. L. Chem. Cent. J. 2015, 9, 30] as per the SI (CIF). Full refinement details are given in the Supporting Information (CIF); Crystallographic data have been deposited with the Cambridge Crystallographic Data Centre (CCDC 2413862) and can be obtained via www.ccdc.cam.ac.uk/data_request/cif.

Figure S1. Crystal structure of compound **35** with the ellipsoids drawn at the 50% probability level.

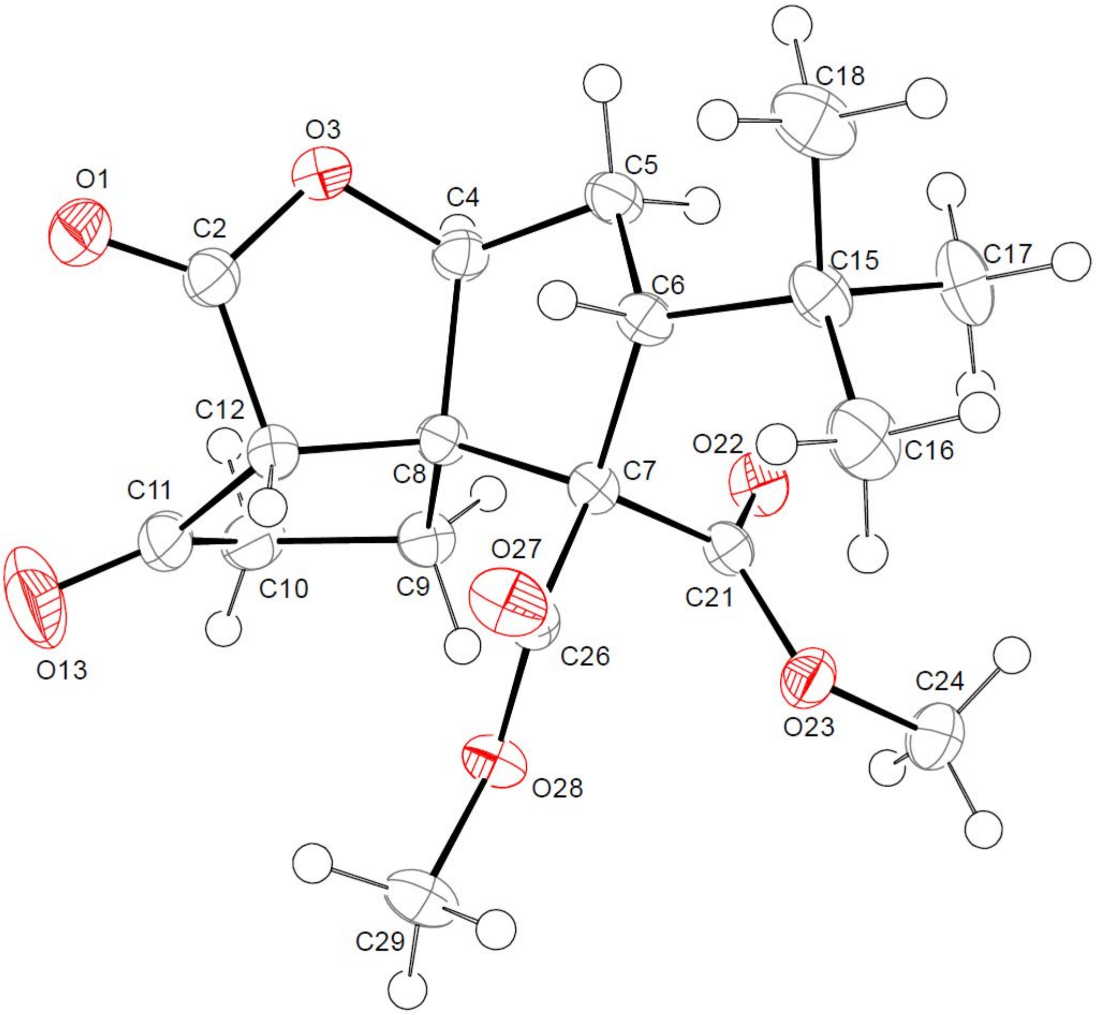

Table S3. Crystal data and structure refinement for 35.

CCDC code	2413862	
Empirical formula	$C_{18}H_{24}O_7$	
Formula weight	352.38	
Temperature	150 K	
Wavelength	1.54184 Å	
Crystal system / Space group	Monoclinic /	P 2_1/c
Unit cell dimensions	a = 11.44610(10) Å	a= 90°.
	b = 10.29590(10) Å	b= 92.0781(12)°.
	c = 15.0471(2) Å	g = 90°.
Volume	1772.10(3) $Å^3$	
Z	4	
Density (calculated)	1.321 Mg/m^3	
Absorption coefficient	0.849 mm^{-1}	
F(000)	752	
Crystal size	0.26 x 0.23 x 0.15 mm^3	
Theta range for data collection	3.864 to 76.425°	
Index ranges	-14<=h<=12, -12<=k<=12, -18<=l<=18	
Reflections collected	21497	
Independent reflections	3701 [R(int) = 0.025]	
Completeness to theta = 74.897°	99.9 %	
Absorption correction	Semi-empirical from equivalents	
Max. and min. transmission	0.88 and 0.78	
Refinement method	Full-matrix least-squares on F^2	
Data / restraints / parameters	3701 / 0 / 227	
Goodness-of-fit on F^2	1.0024	
Final R indices [I>2sigma(I)]	R1 = 0.0335, wR2 = 0.0875	
R indices (all data)	R1 = 0.0353, wR2 = 0.0892	
Extinction coefficient	20(4)	
Largest diff. peak and hole	0.35 and -0.25 e.$Å^{-3}$	

6. References

1. Ogiwara, Y.; Takahashi, K.; Kitazawa, T.; Sakai, N., *J. Org. Chem.* **2015,** *80* (6), 3101-3110.
2. Hosomi, A.; Sakurai, H., *J. Am. Chem. Soc.* **1977,** *99* (5), 1673-1675.
3. Yu, W.; Mei, Y.; Kang, Y.; Hua, Z.; Jin, Z., *Org. Lett.* **2004,** *6* (19), 3217-3219.
4. Priebe, H.; Hopf, H., *Angew. Chem., Int. Ed.* **1982,** *21* (4), 286-286.
5. Piers, E.; Skerlj, R. T., *Can. J. Chem.* **1994,** *72* (12), 2468-2482.
6. Lee, S.; Ryu, D. H.; Yun, J., *Adv. Synth. Catal.* **2021,** *363* (9), 2377-2381.
7. Krylov, I. B.; Terent'ev, A. O.; Timofeev, V. P.; Shelimov, B. N.; Novikov, R. A.; Merkulova, V. M.; Nikishin, G. I., *Adv. Synth. Catal.* **2014,** *356* (10), 2266-2280.
